# Temporal omics analysis in Syrian hamsters unravel cellular effector responses to moderate COVID-19

**DOI:** 10.1038/s41467-021-25030-7

**Published:** 2021-08-11

**Authors:** Geraldine Nouailles, Emanuel Wyler, Peter Pennitz, Dylan Postmus, Daria Vladimirova, Julia Kazmierski, Fabian Pott, Kristina Dietert, Michael Muelleder, Vadim Farztdinov, Benedikt Obermayer, Sandra-Maria Wienhold, Sandro Andreotti, Thomas Hoefler, Birgit Sawitzki, Christian Drosten, Leif E. Sander, Norbert Suttorp, Markus Ralser, Dieter Beule, Achim D. Gruber, Christine Goffinet, Markus Landthaler, Jakob Trimpert, Martin Witzenrath

**Affiliations:** 1 Charité – Universitätsmedizin Berlin, Corporate Member of Freie Universität Berlin and Humboldt-Universität zu Berlin, Division of Pulmonary Inflammation, Berlin, Germany; 2 Berlin Institute of Health (BIH), Berlin, Germany; 3 Berlin Institute for Medical Systems Biology (BIMSB), Max Delbrück Center for Molecular Medicine in the Helmholtz Association (MDC), Berlin, Germany; 4 Charité – Universitätsmedizin Berlin, Corporate Member of Freie Universität Berlin and Humboldt-Universität zu Berlin, Institute of Virology, Berlin, Germany; 5 Institute of Virology, Freie Universität Berlin, Berlin, Germany; 6 Institute of Veterinary Pathology, Freie Universität Berlin, Berlin, Germany; 7 Veterinary Centre for Resistance Research, Freie Universität Berlin, Berlin, Germany; 8 Charité – Universitätsmedizin Berlin, Corporate Member of Freie Universität Berlin and Humboldt-Universität zu Berlin, Core Facility – High-Throughput Mass Spectrometry, Berlin, Germany; 9 Berlin Institute of Health at Charité – Universitätsmedizin Berlin, Core Unit Bioinformatics, Berlin, Germany; 10 Bioinformatics Solution Center, Freie Universität Berlin, Berlin, Germany; 11 Charité – Universitätsmedizin Berlin, Corporate Member of Freie Universität Berlin and Humboldt-Universität zu Berlin, Institute of Medical Immunology, Berlin, Germany; 12 Charité – Universitätsmedizin Berlin, Corporate Member of Freie Universität Berlin and Humboldt-Universität zu Berlin, Department of Infectious Diseases and Respiratory Medicine, Berlin, Germany; 13 The Francis Crick Institute, Molecular Biology of Metabolism Laboratory, London, UK; 14 Charité – Universitätsmedizin Berlin, Corporate Member of Freie Universität Berlin and Humboldt-Universität zu Berlin, Department of Biochemistry, Berlin, Germany; 15 IRI Life Sciences, Institute for Biology, Humboldt-Universität zu Berlin, Berlin, Germany; 16 German Center for Lung Research (DZL), Berlin, Germany

**Keywords:** Mechanisms of disease, Adaptive immunity, Viral infection, Innate immunity, Viral infection

## Abstract

In COVID-19, immune responses are key in determining disease severity. However, cellular mechanisms at the onset of inflammatory lung injury in SARS-CoV-2 infection, particularly involving endothelial cells, remain ill-defined. Using Syrian hamsters as a model for moderate COVID-19, we conduct a detailed longitudinal analysis of systemic and pulmonary cellular responses, and corroborate it with datasets from COVID-19 patients. Monocyte-derived macrophages in lungs exert the earliest and strongest transcriptional response to infection, including induction of pro-inflammatory genes, while epithelial cells show weak alterations. Without evidence for productive infection, endothelial cells react, depending on cell subtypes, by strong and early expression of anti-viral, pro-inflammatory, and T cell recruiting genes. Recruitment of cytotoxic T cells as well as emergence of IgM antibodies precede viral clearance at day 5 post infection. Investigating SARS-CoV-2 infected Syrian hamsters thus identifies cell type-specific effector functions, providing detailed insights into pathomechanisms of COVID-19 and informing therapeutic strategies.

## Abstract (web summary)

The immune response is key in determining disease severity of COVID19. Here Nouailles et al., apply bulk proteomics and scRNA-Seq of lung and blood samples of SARS-CoV-2 infected Syrian hamsters and provide a temporal atlas of the systemic and pulmonary cellular responses.

## Introduction

The enduring severe acute respiratory syndrome coronavirus 2 (SARS-CoV-2) pandemic has emphasized the urgent need for experimental models to rapidly identify pathomechanisms and therapeutic targets of corona virus disease 2019 (COVID-19).

COVID-19 causes a wide range of disease manifestations, spanning from asymptomatic infections to acute respiratory distress syndrome (ARDS) and fatal multi-organ dysfunction^1^. Disease severity is influenced by age, sex, and specific comorbidities, making it evident that host-specific factors influence the course of the disease and require further investigation. While blood of COVID-19 patients is accessible to detailed longitudinal investigation irrespective of disease severity, and bronchoalveolar lavage (BAL) can be safely performed in intubated patients, pulmonary tissue responses remain inaccessible in mild and moderate COVID-19 courses, since lung tissue is only available upon autopsy from patients with fatal disease. Hence, experimental models of COVID-19 are needed, which reflect the complexity of human responses to SARS-CoV-2 infections, including the spatiotemporal dynamics of airway and alveolar infection, local pulmonary immune responses, the activation of systemic inflammatory, complement and coagulation cascades, the impairment of endothelial barrier function, and also mechanisms of resilience, resolution, and repair.

The hamster family (Cricetinae) is of particular interest for experimental modeling of COVID-19, as we and others have observed that animals without genetic modifications can be infected with SARS-CoV-2 and develop phenotypes ranging from mild to lethal COVID-19, depending on age and species^2–6^. Notably, immune cell influx into the lungs, bronchointerstitial pneumonia and diffuse alveolar damage in hamsters bear resemblance to COVID-19 in human patients^7–9^.

Since its initial description as animal model for SARS-CoV^10^, the Syrian hamster has been used to study different aspects of SARS-CoV and Middle East respiratory syndrome (MERS) coronavirus infection^11–13^. Consequently, it now serves as a versatile non-transgenic rodent model to study SARS-CoV-2 infection and therapeutic interventions such as antiviral treatments, immunomodulatory therapies, and vaccines^3,7,14^. The disease observed in hamster species primarily affects the lower respiratory tract, which more closely resembles the common courses of human disease as opposed to clinically severely affected transgenic mice, in many of which infection of the central nervous system (CNS) is the predominant manifestation of the disease^15,16^.

Despite the advantages of hamster models for investigating COVID-19 pathogenesis, unavailability of molecular tools and reagents for hamsters limits investigations of immuno-pathomechanisms, leaving unanswered how closely SARS-CoV-2 evoked disease in hamsters models human COVID-19. We therefore in-depth evaluated SARS-CoV-2-infected Syrian hamsters (*Mesocricetus auratus*), elucidating the innate and adaptive steps of immunity and pathogenesis by pairing single-cell RNA sequencing (scRNA-Seq) data from lung cells and white blood cells (WBC), histopathology and quantitative proteomics analysis of lungs and blood following nasal SARS-CoV-2-infection of *Mesocricetus auratus*. We compared our findings with own data from scRNA-Seq and proteomics analyses from human biosamples of COVID-19 patients. This enabled in-depth investigations on central COVID-19 pathomechanisms in compartments inaccessible in humans, particularly in moderate disease.

In this work, we show that (i) monocyte-derived macrophages in lungs are exerting the earliest and strongest transcriptional response to infection, (ii) epithelial cells show weak alterations, (iii) early in the infection, endothelial cells strongly express anti-viral, pro-inflammatory, and T cell recruiting genes without evidence for productive infection, and (iv) recruitment of cytotoxic T cells, as well as emergence of IgM antibodies, precedes viral clearance at day 5 post infection.

## Results

### SARS-CoV-2 induces self-resolving moderate pneumonia and robust pulmonary immune cell recruitment in Syrian hamsters

After infection with SARS-CoV-2 (Supplementary Fig. 1), clinical disease manifested in Syrian hamsters with moderate transient weight loss analogous to previous reports (refs. ^2,3,6^, Supplementary Fig. 2a). High viral loads were detected in respiratory tracts at 2, 3, and 5 days post infection (dpi). At 14 dpi, only minimal viral RNA load remained, and no replication-competent virus was detected in the respiratory tract (Supplementary Fig. 2b–d).

Similar to previous observations^2,9^, histopathology identified necrosuppurative bronchitis and bronchointerstitial pneumonia at 2 and 3 dpi, characterized by intrabronchial and intraalveolar infiltration by neutrophils and macrophages as well as severe, diffuse alveolar damage. Numbers and density of infiltrating immune cells, hyperplasia of bronchial and alveolar epithelial cells as well as alveolar and interstitial edema and endothelialitis peaked at 5 dpi. By 14 dpi, cellular influx into alveolar spaces was largely resolved, with fewer neutrophils, macrophages, and lymphocytes observed within the interalveolar septa, while marked hyperplasia of alveolar epithelial cells remained (Supplementary Fig. 2e–n). Again, consistent with previous reports^2,9^ no evidence of thrombotic events were observed.

**Fig. 1:**
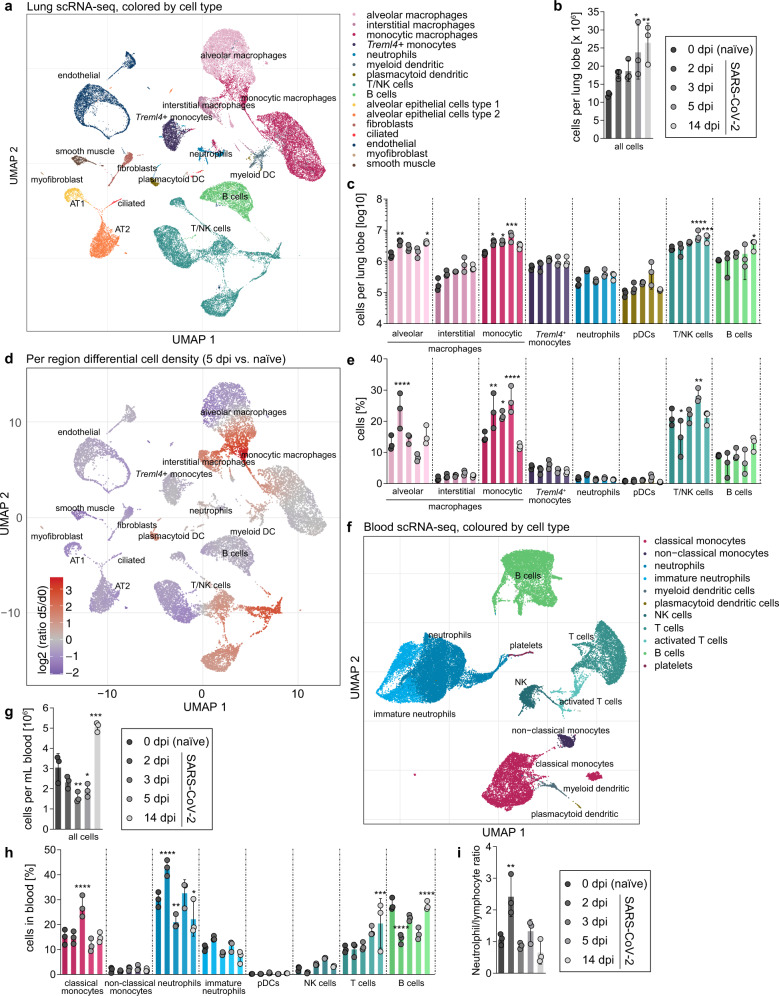
Single-cell dynamics in lungs and blood of SARS-CoV-2 infected Syrian hamsters. a Uniform manifold approximation and projection (UMAP) plot of identified cell populations in Syrian hamster lungs. Colors representing individual cell types are depicted in legend. **b** Cell count of isolated cells per lung lobe over time (2, 3, 5, and 14 days post infection (dpi)) and control group (naive, “d0”). **c** Count of hematopoietic cells per lung lobe in naive hamsters and over time pi. **d** Changes in cellular density of lung cells in UMAP projection. Coloration indicates log2 fold change between control group and 5 dpi. **e** Percentage of hematopoietic cells per lung lobe in naive hamsters and over time pi. **f** UMAP plot of identified cell populations in blood samples. **g** Cell count of isolated cells per mL blood in naive hamsters and over time pi. **h** Percentage of identified cell populations in blood samples over time pi and naive animals. **i** Neutrophil–lymphocyte ratio in blood samples over time pi and naive animals. **a**, **d** and **f** Clusters defined by Louvain clustering, *n* = 3 per time point. **b**, **c**, **e**, **g**, **h** and **i** Bar plots are plotted per cell type in the order: naive, 2 dpi, 3 dpi, 5 dpi, and 14 dpi (colors fade from dark to light). Data display means ± SD. *n* = 3 per time point. Ordinary one-way ANOVA, Dunnett’s (**b**, **g**, **i**) and Šídák’s multiple comparisons (**c**, **e**, **h**) test versus corresponding 0 dpi (naive). ^∗^*p* < 0.05, ^∗∗^*p* < 0.01, ^∗∗∗^*p* < 0.001, ^∗∗∗∗^*p* < 0.0001. AT1 and AT2: alveolar epithelial cell type 1 and 2, DC: dendritic cells, NK, natural killer cells. Exact *p*-values in order of appearance: **b** ^∗^*p* = 0.0255; ^∗∗^*p* = 0.0078 **c** alveolar: ^∗∗^*p* = 0.0041; ^∗^*p* = 0.0102, monocytic: ^∗^*p* = 0.0213; ^∗^*p* = 0.0226; ^∗∗∗∗^*p* < 0.0001; T/NK: ^∗∗∗∗^*p* < 0.0001; ^∗∗∗^*p* = 0.0002; B cells: ^∗^*p* = 0.0106 **e** alveolar: ^∗∗∗∗^*p* < 0.0001; monocytic: ^∗∗^*p* = 0.0010; ^∗^*p* = 0.0138; ^∗∗∗∗^*p* < 0.0001; T/NK: ^∗^*p* = 0.0225; ^∗∗^*p* = 0.0099 **g** ^∗∗^*p* = 0.0033; ^∗^*p* = 0.0174; ^∗∗∗^*p* = 0.0004 **h** classical monocytes: ^∗∗∗∗^*p* < 0.0001; neutrophils: ^∗∗∗∗^*p* < 0.0001; ^∗∗^*p* = 0.0040; ^∗^*p* = 0.0257; T cells: ^∗∗∗^*p* = 0.0004; B cells: ^∗∗∗∗^*p* < 0.0001; ^∗∗∗∗^*p* < 0.0001 **i** ^∗∗^*p* = 0.0024.

To obtain higher resolution of pulmonary responses, we performed scRNA-Seq. Cell type clusters detected in lungs corresponding to leukocyte-subset-signatures included alveolar, interstitial, and monocytic macrophages, *Treml4*^+^-monocytes, neutrophils, dendritic cells, B, T, and natural killer (NK) cells. We further identified resident cell types, including alveolar epithelial cells type 1 (AT1) and 2 (AT2), ciliated epithelial, endothelial, and smooth muscle cells, and fibroblasts (Figs. 1a, S3a). By integrating scRNA-Seq-derived cell frequencies with manually counted cell numbers over time, we mapped dynamics of infection-induced pulmonary leukocyte recruitment compared to uninfected animals (Fig. 1b, c). The influx of monocyte-derived macrophages peaked at 5 dpi. NK and T lymphocyte recruitment to lungs was first detected at 5 dpi and peaked at day 14 (Fig. 1c). Peak of lung inflammation on 5 dpi (Supplementary Fig. 2l) coincided with the highest proportion of inflammatory macrophages (monocytic macrophage cluster) and proliferating cytotoxic cells (T/NK cell cluster) among lung cells (Supplementary Fig. 3b).

Notably, despite pronounced neutrophilic bronchitis (Supplementary Fig. 2g), overall neutrophil frequencies remained low and changes were non-significant. In line with histopathology, the peak of neutrophil recruitment was at 2 dpi, when neutrophil proportions presented ~3% of lung cells (Fig. 1e). In contrast, monocytic macrophages population at day 5 peaked at ~25% of lung cells (Figs. 1d, e, S3b). Relative numbers of pulmonary tissue cell subsets fluctuated mildly, declining proportionally as inflammatory cell influx rose (Supplementary Fig. 3c, d). At 14 dpi, increased numbers of AT2 matched histopathology observations of epithelial hyperplasia, indicating tissue repair (Supplementary Figs. 2i, k, 3c).

Analogously, we analyzed scRNA-Seq data from WBC populations to study systemic responses evoked by pulmonary SARS-CoV-2 infection. Detected cell populations included neutrophils, monocytes, dendritic, NK, B, and T cells and various subpopulations thereof (Figs. 1f, S3e). Infected hamsters displayed significant leukopenia at 3 and 5 dpi. By 14 dpi this trend was inverted and peripheral blood leukocyte numbers were significantly higher than in naive animals (Figs. 1g, S3f). Increased proportions of neutrophils were found at 2 dpi and increasing proportions of T cells at 14 dpi (Figs. 1h, S3b). Notably, calculated neutrophil–lymphocyte ratios only transiently increased at 2 dpi to a minor extent, matching observations in humans with non-severe as opposed to severe COVID-19^17,18^ (Fig. 1i). Overall, scRNA-Seq cell profiling defined kinetics of immune cell trafficking in greater detail.

### Bulk transcriptomics, proteomics, and single-cell RNA sequencing reveal activation of anti-SARS-CoV-2 pro-inflammatory immunity in hamsters

After evaluating immune cell dynamics, we performed bulk RNA-sequencing of lungs and blood and matched proteomics of lungs and serum to gain insights into alterations of SARS-CoV-2-induced immune responses.

Gene ontology (GO) enrichment analysis of the infection-triggered most differentially expressed genes in lungs and blood provided expected GO terms such as defense response to virus, innate immune response, and cell activation (Supplementary Fig. 4a, b). Pulmonary gene sets related to type 1 interferon (IFN) signaling correlated with the presence of viral RNA, thus vanished by 14 dpi. Similarly, pulmonary gene sets related to response to interferon-gamma (Ifng) were highest at 5 dpi (Supplementary Fig. 4c). In blood, type 1 IFN signaling and response to IFN-γ gene sets were highest at 2 and 3 dpi (Supplementary Fig. 4d), perhaps reflecting recruitment of specific cells from blood to lungs. Overall, bulk RNA-seq identified an anti-viral immune response that was effectively resolved when the virus was cleared.

**Fig. 2:**
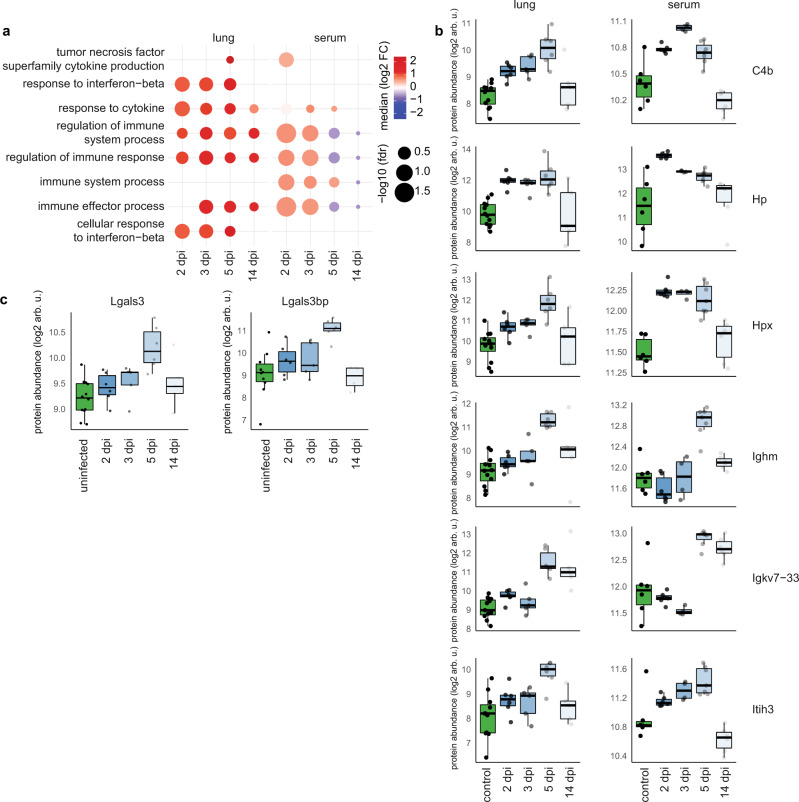
Proteomics analysis recapitulates transcriptomics and human COVID-19 patient data. **a** Temporal evolution of gene ontology/biological process terms connected with immune system response in lung tissue (left part) and in serum (right part), for the indicated time points compared to samples from uninfected control animals. Enriched terms were filtered for terms mentioning immune, interferon, neutrophil, T cell and B cell and attained false discovery rates (fdr) below 0.2 at least at one-day post infection (dpi) in lungs or serum. The redundancy of terms was then reduced using REVIGO. Size of dots correspond to the inverse of the false discovery rate, color corresponds to median log2(fold change (FC)) of proteins, contributing to the term. **b** Expression values for differentially regulated proteins in hamster serum (control versus infected at 3 dpi, *p* < 0.01) and lung (control vs. infected at 5 dpi, *p* < 0.01) that correlate with disease severity in human plasma. Controls from different days are plotted together. Lung sample group sizes: control: *n* = 12, 2 dpi: *n* = 6, 3 dpi: *n* = 5, 5 dpi: *n* = 6, 14 dpi: *n* = 5. Serum sample group sizes: control: *n* = 6, 2 dpi: *n* = 6, 3 dpi: *n* = 4, 5 dpi: *n* = 7, 14 dpi: *n* = 6. All non-missing values are shown. **c** Expression values for the differentially expressed (control vs. infected at 5 dpi, *p* < 0.01) proteins Lgals3 and Lgals3bp (only detected in lung samples). Individual data points are shown in shades of gray. Lung sample group sizes: control: *n* = 12, 2 dpi: *n* = 6, 3 dpi: *n* = 5, 5 dpi: *n* = 6, 14 dpi: *n* = 5. **b**, **c** Box plots, the middle line in the boxplot displays the median, the box indicates the first and third quartile, whiskers the 1.5 interquartile range (IQR). arb.u.: arbitrary units.

The proteome host response was in line with the sequencing data, with differentially expressed serum proteins peaking at 3 dpi and lung proteins at 5 dpi (Supplementary Fig. 4e). Agreement between bulk RNA sequencing and proteomics was consistent in lungs at 5 dpi (*r* = 0.9) (Supplementary Fig. 4f). The principal component analysis (PCA) is detailed in the supplementary note, showing that the maximal response was observed at 5 dpi (supplementary note PCA on bulk transcriptomic data and PCA on bulk proteomics data). Functional terms connected with immune response, such as innate and adaptive immunity, activation of complement system, humoral immune response, and regulation of immune system processes were most enriched (Figs. 2a, S4g). In lungs, response to interferon-beta peaked at 3 dpi and stayed high until 5 dpi (Fig. 2a). Most processes were resolved by 14 dpi.

We next aimed at comparing our data to published datasets from COVID-19 patients. In hamster serum, 37 differentially expressed proteins were identified (α = 0.01, providing FDR below 6%), 17 compared to control and 31 proteins compared to 14 dpi when most effects are resolved. Of 31 proteins, 20 have been reported in human COVID-19 studies, 7 (Actg1, Apoa1, Apoc1, Gsn, Hp, Itih3, Lbp) of which correlate with disease severity^19^, all showing the same direction (Supplementary Fig. 4h).

In hamster lungs, at peak response (5 dpi) we identified 150 differentially expressed proteins. 13 differentially expressed proteins have been reported to be regulated in human plasma^20^ with 9 showing the same trend (Supplementary Table 2). Of these, 6 proteins (C4b, Hp, Hpx, Ighm, Igkv7-33, Itih3) are also changed in hamster serum (Fig. 2b). Although a comparison to moderate disease in human lung tissue is not possible, 22 proteins are reported to be regulated in human BAL fluid of critically ill patients^21^. The few proteins showing opposite regulation to COVID-19 patients were confirmed by bulk sequencing. Five out of 7 conflicting responses, namely C4b, Hpx, Rbp4, Cfd, and Agt in hamster serum compared to human plasma as well as 8 out of 12 for hamster lung tissue compared to human BAL were confirmed.

**Fig. 3:**
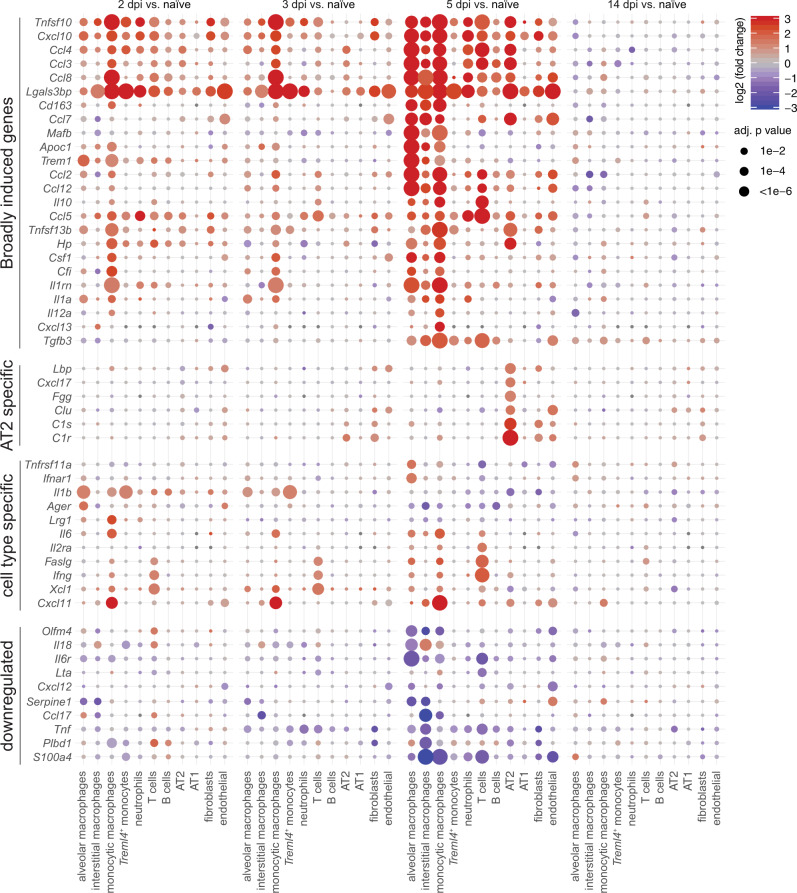
Induction of inflammatory mediators are strongest and earliest in myeloid cells. Dotplots of differentially expressed cytokines and inflammatory mediators in lungs. Shown are genes that display a significant absolute log2-transformed fold change of at least one in at least one comparison, and are grouped into four categories indicated on the left. Coloration and point size indicate log2-transformed fold changes (FC) and *p-*values, respectively, of genes at 2 days post infection (dpi) relative to control groups (naive). Adjusted (adj) *p-*values were calculated by DEseq2 using Benjamini–Hochberg corrections of two-sided Wald test *p*-values. Genes are ordered by unsupervised clustering, cell type as in Fig. 1.

Next, we refined transcriptome analyses with scRNA-Seq and related identified bulk GO terms to cell types, concentrating on inflammatory mediators (Fig. 3). Indeed, various pro-inflammatory chemokines were expressed by lung cells and showed distinct cellular and temporal expression patterns. Classical pro-inflammatory cytokines, e.g., *Il1a* and *Il1b* transcripts were elevated only early at 2 and 3 dpi in alveolar and monocytic macrophages, and alveolar macrophages and *Treml4*^+^ monocytes, respectively. By 5 dpi, AT2 cells showed a unique range of upregulated inflammatory mediators such as *Cxcl17*, *Lipopolysaccharide Binding Protein (Lbp)*, *fibrinogen gamma gene (Fgg)*, and *clusterin (Clu)*. At the same time point, we found, among others, downregulation of the *Il6 receptor* (also known as CD126) and *S100a4*. Transiently decreased expression of these two inflammatory mediators might be part of the efficient yet self-regulating inflammatory response in this disease model. *Galectin 3-binding protein* (*Lgals3bp*) gene stood out as being upregulated in many cell types from 2 dpi to 5 dpi (Fig. 3). Notably, we likewise measured increased levels of Lgals3bp protein in lungs (Fig. 2c), which was shown to be regulated also in plasma of COVID-19 patients and correlated with severity^19^.

Taken together, we identified clear changes in transcriptome, proteome and pro-inflammatory signatures on single-cell level in response to SARS-CoV-2 infection, displaying highly active immune responses that to large extents were also described in COVID-19 patients.

### Migratory myeloid cells dominate pulmonary transcriptional response to SARS-CoV-2 infection in Syrian hamsters and COVID-19 patients

**Fig. 4:**
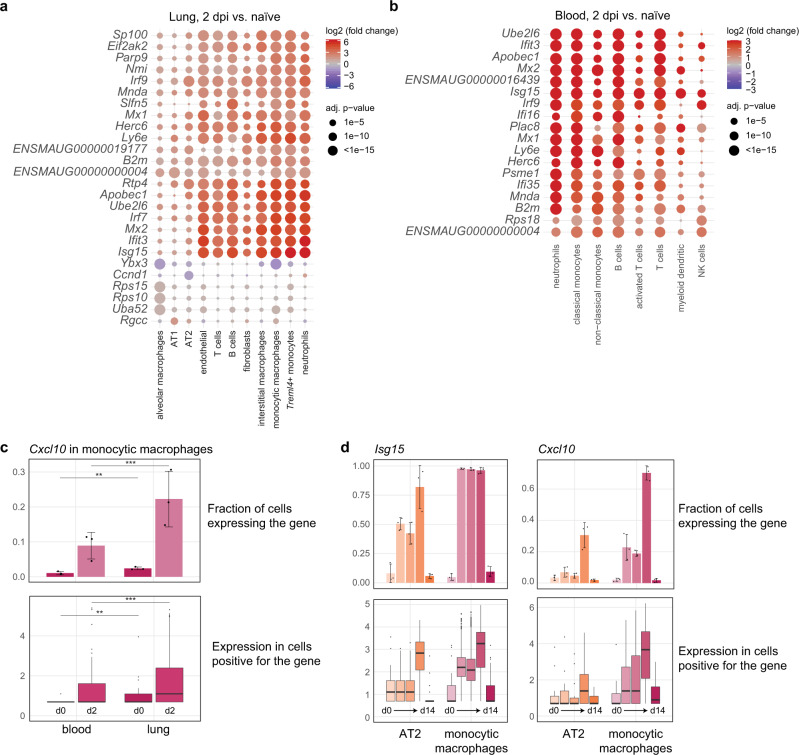
Transcriptional response to SARS-CoV-2 infection is strong in myeloid and moderate in epithelial cells. **a**, **b** Dotplot of differentially expressed genes in lungs (**a**) and blood (**b**). Shown are genes that are in at least one cell type among the top four most changing genes as ranked by adjusted *p*-value. Example: top 4 most changing genes in alveolar macrophages are *Ybx3*, *Rps15*, *Rps10*, and *Uba52*. Coloration and point size indicate log2-transformed fold changes (FC) and *p*-values, respectively, of genes at 2 days post infection (dpi) relative to control groups (naive). Adjusted (adj.) *p*-values were calculated by DEseq2 using Benjamini–Hochberg corrections of two-sided Wald test *p*-values. Cell types and genes are ordered by unsupervised clustering. **c** Top, fraction of monocytic macrophages expressing *Cxcl10* in blood and lungs from naive animals and 2 dpi. Bottom, boxplots of *Cxcl10* gene expression values of cells positive for *Cxcl10* expression. Significance levels were calculated using two-sided generalized linear mixed-effects models for bar plots and a two-sided Wilcoxon rank-sum test on all cells (i.e., not only the ones expressing the gene) for boxplots. ^∗^*p* < 0.05; ^∗∗^*p* < 0.01; ^∗∗∗^*p* < 0.0001. See “Methods” for details. **d** As in (**c**), but only in lungs, all time points and both alveolar epithelial cells type 2 (AT2) and monocytic macrophages. Displayed are values for *Isg15* (left) and *Cxcl10* (right). For all bar plots, data display means ± SD, *n* = 3 per time point. For all boxplots, cell gene expression data derived from *n* = 3 animals per time point. The middle line in the boxplot displays the median, the box indicates the first and third quartile, whiskers the 1.5 interquartile range (IQR). Outliers beyond are marked by single dots. AT1 and AT2: alveolar epithelial cell type 1 and 2, DC: dendritic cells, NK, natural killer cells; d0: day 0 = naive, d14: 14 dpi. Exact *p*-values in (**c**): upper panel: ^∗∗^*p* = 0.0022; ^∗∗∗^*p* < 0.0001; lower panel: ^∗∗^*p* = 0.0015; ^∗∗∗*p*^ < 0.0001.

To pinpoint individual roles of identified cells in anti-SARS-CoV-2 immunity, we analyzed the 15–20 most differently expressed genes in each cell subset. In early stages of infection at 2 dpi, robust, local transcriptome changes were observed primarily in lung monocytic and interstitial macrophages, neutrophils, and endothelial cells, whereas AT1 and AT2 epithelial cells and alveolar macrophages showed comparably little change in mRNA expression (Fig. 4a). A common set of anti-viral effector genes was found upregulated in many cell types^22^. These include e.g., *interferon-stimulated gene 15* (*Isg15*), *MX dynamin like GTPase (Mx)1, Mx2, Interferon-induced protein with tetratricopeptide repeats 3* (*Ifit3)*, and *Sp100*, as well as transcription factors, such as *Interferon regulatory factor* (*Irf) 7* and *Irf9* (Fig. 4a, Supplementary Fig. 5a). Blood transcriptome analysis recapitulated this early transcriptional activity at 2 dpi (Fig. 4b, Supplementary Fig. 5b), but declined by 5 dpi, whereas the signature persisted in the lungs until 14 dpi (Supplementary Fig. 5a, b).

Genes that differed most between classical blood monocytes and their counterparts in lungs encoded chemokines and activation markers, including *CXC chemokine ligand 10* (*Cxcl10)*, *Slamf9*, *Il18bp*, *Ifitm2*, *Ccl8*, *Ccl4*, and *Ccl5* (Fig. 4c, Supplementary Fig. 5c), indicating that activation and acquisition of effector function occurred in lungs. Although AT2 cells are a main target of SARS-CoV-2 in lungs^23^, they displayed weaker and later transcriptional changes upon infection compared to monocytic macrophages (Figs. 4a, d, S5a, d). Notably, at 14 dpi differential transcriptional responses related to defense resolved in blood and lung cells. Instead, we observed upregulation of cell cycle proliferation genes in AT2 cells including *Marker of Proliferation Ki-67* (*Mki67*), *Ubiquitin-conjugating enzyme E2 C* (*Ube2c*), *Aurora B kinase* (*Aurkb*), and *Stathmin* (*Stmn1*) (Supplementary Fig. 5a). This transcriptome profile indicated initiation of a repair program by AT2 cells, proliferating to replace damaged AT1 cells^24^. Finally, we put our hamster lung scRNA-Seq data in context with BAL scRNA-Seq data from patients with moderate-to-severe COVID-19^25^ and healthy controls^26^. As in hamster data, we observed stronger transcriptional responses in macrophages compared to epithelial cells (Supplementary Fig. 5e). Furthermore, the upregulated gene program containing e.g., *CXCL10*, *CCL2*, or *CCL8* was substantially overlapping (Supplementary Fig. 5f).

To test whether hamster tissue responses are representative of infected human epithelial cells, we next referred to our scRNA-Seq dataset derived from nasopharyngeal swabs of 19 COVID-19 patients and 5 healthy controls^27^. Here again, human and hamster epithelial cells derived from infected individuals and animals, respectively, showed a similar, moderate induction of most inflammatory mediators (Supplementary Fig. 6a, b). As notable difference, strong induction of neutrophil-recruiting chemokines targeting CXC chemokine receptor (CXCR) 2, such as *CXCL1*, *CXCL3*, *CXCL6*, and *CXCL8*, were found only in human basal and secretory cells with severe COVID-19 but were absent in moderately-ill Syrian hamsters (Supplementary Fig. 6a, Fig. 3). Aside from the epithelial inductions of neutrophil-attractant transcripts unique to severe COVID-19, SARS-CoV-2 infected hamsters and patients displayed strikingly similar pro-inflammatory immune profiles specifically in migratory myeloid cells.

### Early activation of TLR/NF-kB-dependent transcription of pro-inflammatory cytokines in monocytic macrophages by SARS-CoV-2 infection

**Fig. 5:**
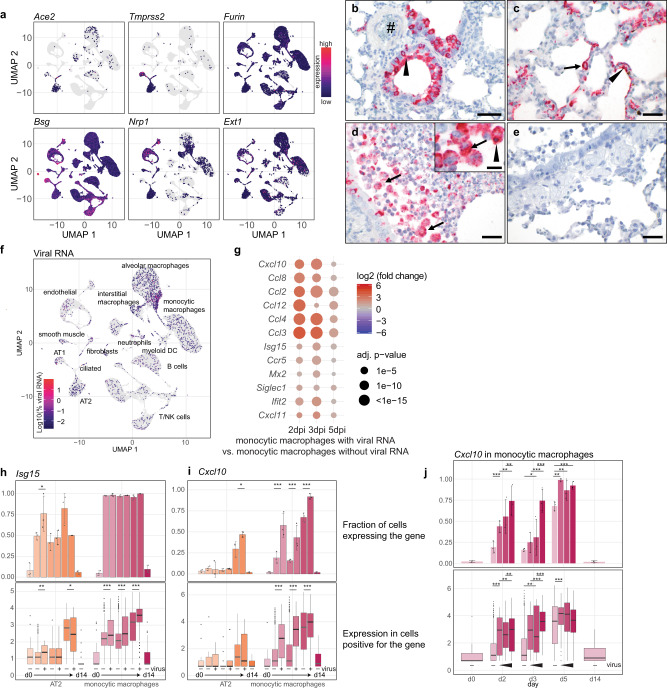
Virus RNA in monocytic macrophages leads to dose-dependent activation of pro-inflammatory cytokines by TLR signaling. **a** Feature plots of entry factor expression in Uniform manifold approximation and projection (UMAP) projection. Coloration indicates expression values of indicated genes. **b**–**e** Detection of viral RNA by in situ-hybridization. Labeled are supposed endothelium (**b**, hash), bronchial epithelial cells (**b**, arrowhead), AT1 (**c**, arrowhead) and AT2 (**c**, arrow). (**d**, inset), macrophages containing viral RNA and cell debris (arrowhead), and an example of high levels of viral RNA without cell debris in the inset (arrow). For **b**–**e** red signals viral RNA and blue hemalaun counterstain. Time points: **b,** **c** from 2 dpi, **d** from 3 dpi, **e** staining control. Bars: **b**, **d**, **e** = 50 µm, **c** = 100 µm, Inset in **d** = 20 µm. Micrographs representative of *n* = 6 per time point pi. **f** Cells in the UMAP projection are colored by amount of viral RNA (log10 transformed percentage of viral RNA per cell), along with overview of identified cell types in lungs. **g** Dotplot of cytokine expression in monocytic macrophages containing viral RNA compared to those without viral RNA. Coloration and point size indicate log2 fold change and adjusted (adj.) *p-*value for each time point 2, 3, and 5 dpi. Adjusted (adj.) *p*-values were calculated by DEseq2 using Benjamini–Hochberg corrections of two-sided Wald test *p*-values. **h**, **i** Bar- and boxplots of *Isg15* (h) and *Cxcl10* (i) gene expression in AT2 and monocytic macrophages along, comparing cells containing viral RNA to those without for 2, 3, and 5 dpi. Barplot shows percentage of cells positive for respective gene. Boxplots show gene expression levels in cells positive for respective gene, **j** Bar- and boxplots of *Cxcl10* in monocytic macrophages and fraction of *Cxcl10* positive cells for each time point pi and naive animals, with cells grouped by increasing virus levels for 2, 3, and 5 dpi. **h**–**j** Barplots, data display means ± SD, *n* = 3 animals per time point. Significance levels calculated using two-sided generalized linear mixed-effects models. For box plots, the middle line in the boxplot displays the median, the box indicates the first and third quartile, whiskers the 1.5 interquartile range (IQR), cell gene expression data derived from *n* = 3 animals per time point two-sided Wilcoxon rank-sum test on all cells (i.e., not only the ones expressing the gene) for boxplots. ^∗^*p* < 0.05; ^∗∗^*p* < 0.01; ^∗∗∗^*p* < 0.0001. See “Methods” for details. AT1 and AT2: alveolar epithelial cell type 1 and 2, DC: dendritic cells, NK, natural killer cells; d0: day 0 = naive, d14: 14 dpi. Exact *p*-values in order of appearance in (**h**) upper panel: ^∗^*p* = 0.0338; lower panel: ^∗∗^*p* = 0.0093; ^∗^*p* = 0.014; ^∗∗∗^*p* < 0.0001; ^∗∗∗^*p* < 0.0001; ^∗∗∗^*p* < 0.0001; **i** upper panel: ^∗^*p* = 0.0270; ^∗∗∗^*p* < 0.0001; ^∗∗∗^*p* < 0.0001; ^∗∗∗^*p* < 0.0001; lower panel: ^∗∗∗^*p* < 0.0001*;* ^∗∗∗^*p* < 0.0001*;* ^∗∗∗^*p* < 0.0001; **j** upper panel: ^∗∗∗^*p* < 0.0001; ^∗∗^*p* = 0.0004; ^∗∗^*p* = 0.0022; ^∗^*p* = 0.0131; ^∗∗∗*p*^ < 0.0001; ^∗∗∗^*p* < 0.0001; ^∗∗^*p* = 0.0003; ^∗∗^*p* = 0.0033; ^∗∗∗^*p* < 0.0001; lower panel: ^∗∗∗^*p* < 0.0001; ^∗∗^*p* = 0.0015; ^∗∗^*p* = 0.0064; ^∗∗^*p* = 0.0034; ^∗∗∗^*p* < 0.0001; ^∗∗∗^*p* < 0.0001; ^∗∗∗*p*^ < 0.0001.

Next, we asked whether observed cellular transcriptional responses to SARS-CoV-2 infection were influenced by the presence of virus in individual cell types. First, we determined fractions of cells expressing SARS-CoV-2 entry receptors, *Angiotensin-converting enzyme 2* (*Ace2) and transmembrane serine protease 2* (*Tmprss2)*, putative alternative receptors, *Basigin (Bsg)* and *Furin*, and cofactors, such as neuropilins (*Nrp1*), and heparan sulfate (*exostosin-1*, *Ext1)*)^28–30^ in hamster lungs (Fig. 5a, Supplementary Table 3). Ciliated epithelial cells most frequently expressed *Ace2* (~4–22%), as did a smaller proportion of AT2 cells (~3–5%) (Supplementary Table 3). By in situ-hybridization, we visualized SARS-CoV-2 RNA in bronchial epithelial cells (Fig. 5b), and AT1 (Fig. 5c, arrowhead) and AT2 (Fig. 5c, arrow) cells, whereas endothelial cells were consistently devoid of virus (Fig. 5b, hash). Importantly, viral RNA was detected in high numbers of intrabronchial and intraalveolar macrophages (Fig. 5d, arrows) at early time points. A fraction of macrophages contained high loads of virus without cell debris, pointing toward uptake of cell-free virus (Fig. 5d, inset). For comparison, a control staining section of alveoli is shown (Fig. 5e).

ScRNA-Seq data suggested that most viral RNA content was found in monocytic macrophages, and not in epithelial cells (Fig. 5f, Supplementary Table 3). For epithelial and endothelial cells, frequencies of virus-positive cells were highest at 3 dpi, declining by 5 dpi to become absent at 14 dpi, indicating removal of virus-containing cells (Supplementary Table 3). In contrast, alveolar macrophages showed highest viral loads at 5 dpi (~25%), paralleling decline of virus-positive tissue cells, thus pointing toward potential increase in phagocytic activities (Supplementary Table 3). We attempted to identify viral replication from our scRNA-Seq data without obtaining significant results (analysis details and results depicted in supplementary note assessing viral replication from sequencing data).

To further investigate how cell-specific gene expression is modulated by cell-associated viral RNA, we tested the correlation between gene expression and viral load in monocytic macrophages. We first compared gene expression levels in monocytic macrophages that did (vRNA^+^), or did not (vRNA^−^) contain viral RNA (Figs. 5g, S7a). This revealed a set of genes that only at early disease stages (2 dpi, 3 dpi), were present at higher levels in vRNA^+^ monocytic macrophages (Supplementary Fig. 7a). Gene ontology and KEGG pathway analysis showed that this gene set was enriched for Toll-like receptor (TLR) signaling (Supplementary Fig. 7b). Specifically, this gene set contained a range of pro-inflammatory cytokines such as *Cxcl10* or *Ccl2* (Fig. 5g), which are activated by the NF-kB pathway downstream of TLRs^31^. On the other side, expression levels of NF-kB independent ISGs such as *Isg15* or *Mx2*, induced by interferons or cytosolic RNA sensors^32^, were only slightly more elevated in vRNA^+^ compared to vRNA^–^ monocytic macrophages (Fig. 5g). Therefore, we investigated representative genes, *Isg15* and *Cxcl10*, in more detail (Fig. 5h, i). *Isg15* expression was found in about 2/3 of AT2 and all monocytic macrophages at 2 dpi. *Isg15* levels in gene-positive cells were higher at 2 dpi in vRNA^+^ AT2 cells and at 2, 3, and 5 dpi vRNA^+^ monocytic macrophages compared to corresponding vRNA^–^ cells (Fig. 5h). In comparison, *Cxcl10* was nearly absent in AT2 cells at 2 dpi and 3 dpi. *Cxcl10*-positive cell fractions and gene expression levels were significantly higher in vRNA^+^ monocytic macrophages compared to vRNA^–^ cells at 2, 3, and 5 dpi (Fig. 5i). We further analyzed dose-dependency of this transcriptional response to virus in monocytic macrophages. Cells were binned in three groups of equal size with increasing content of viral RNA. We found that at earlier time points (2 and 3 dpi), but not at 5 dpi, cells with higher amounts of viral RNA-signal, also expressed more *Cxcl10* (Fig. 5j).

Overall, this indicated that sensing of viral RNA activated monocytic macrophages in a dose-dependent manner, leading to increase of NF-kB-regulated pro-inflammatory chemokines. At 5 dpi, broad inflammation likely masked direct viral RNA-triggered responses by activating expression of pro-inflammatory genes in vRNA^+^ and vRNA^–^ cells equally. In contrast, AT2 cells showed less activation of both NF-kB-dependent and -independent transcriptional responses as compared to monocytic macrophages.

### Endothelial cells participate in anti-viral and pro-inflammatory responses

Having observed vast similarities of human and Syrian hamster immune responses in moderate SARS-CoV-2 infection on transcriptomic and proteomic levels, we next turned our attention to dissection of molecular mechanisms in lung tissue compartments that have so far not been assessed longitudinally in moderate COVID-19 patients, since invasive tissue sampling is hardly possible.

**Fig. 6:**
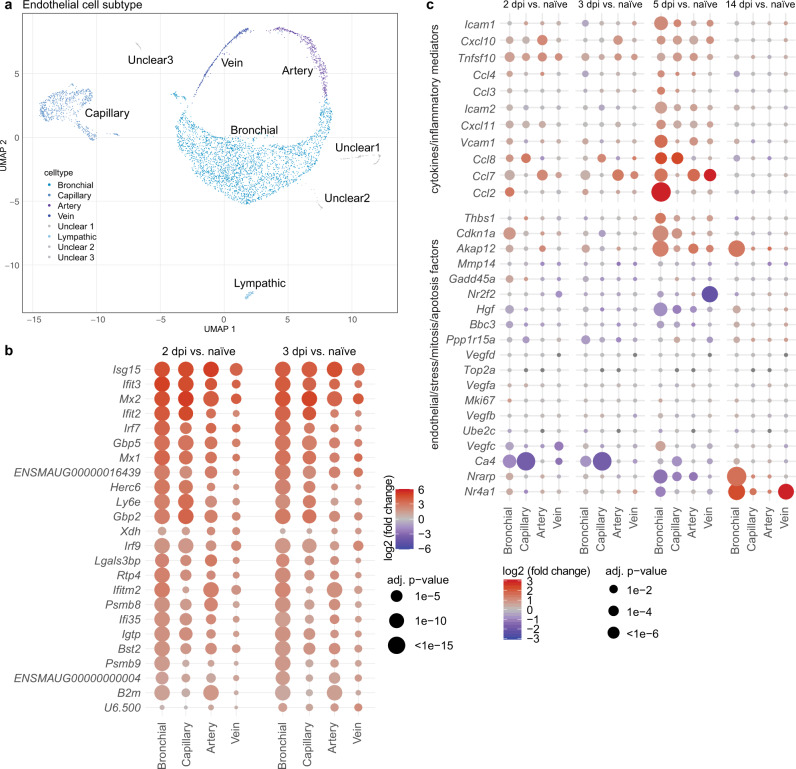
Endothelial cells show subtype and time specific activation of cytokines. **a** Uniform manifold approximation and projection (UMAP) plot of lung endothelial cell subpopulations. Clusters defined by Louvain clustering, *n* = 3 per time point. Colors representing individual cell types are depicted in legend. **b** Dotplot of differentially expressed genes in lung endothelial cell subpopulations over time. Shown are genes that are in at least one cell type among the top 10 most changing genes as ranked by adjusted (adj.) *p*-value. Adjusted (adj.) *p*-values were calculated by DEseq2 using Benjamini–Hochberg corrections of two-sided Wald test *p*-values. **c** Dotplot of differentially expressed genes from two sets (upper set: cytokine/inflammatory mediators; lower set: endothelial/stress/mitosis/apoptosis factors) as indicated in lung endothelial cell subpopulations over time pi. For both **b**, **c** coloration and point size indicate log2-transformed fold changes and *p-*values, respectively, of genes at 2 days post infection (dpi) relative to control groups (naive). Adjusted (adj.) *p-*values were calculated by DEseq2 using Benjamini–Hochberg corrections of two-sided Wald test *p-*values.

Endothelial cells likely participate in COVID-19 pathogenesis^33,34^, but little is known about dynamics of their responses to inflammation in vivo. Subclustering of cells of endothelial origin identified endothelial cells of lymphatic and bronchial vasculature, pulmonary arteries, capillaries, and veins with unique features (Fig. 6a, Supplementary Fig. 8a, b). Interestingly, bronchial endothelial cells, pulmonary artery, and capillary endothelial cells all displayed strong and early anti-viral gene expression profiles at 2 dpi (Fig. 6b). Pulmonary arterial endothelial cells responded most rapidly to infection, with high expression of *Cxcl10*, *Tnfsf10*, and *Ccl7* by 2 dpi (Fig. 6c). Responses of bronchial vasculature, pulmonary capillary and pulmonary vein endothelial cells were similar but delayed, peaking at 5 dpi. In addition to distinct temporal dynamics of endothelial activation in different tissues, we observed a spatial regulation of expression of monocyte and effector T cells attractants. Pulmonary artery and vein endothelial cells preferentially transcribed *Ccl7*, a chemoattractant binding multiple CC receptors, including CCR1-3, CCR5, and CCR10^35^. Pulmonary capillary endothelial cells, however, preferentially expressed the pleiotropic *Ccl8*, binding at least CCR2, CCR3 and CCR5^36^, while bronchial vasculature endothelial cells were characterized by *Ccl2* (Fig. 6c). ICAM-1 and VCAM-1 upregulation, occurs following inflammatory stimuli to allow for leukocyte transmigration^37^, and was highest in bronchial endothelial cells and pulmonary artery cells at 5 dpi, corresponding to influx of T cells (Fig. 6c). Overall lung endothelial cells shared an anti-viral gene profile but revealed distinct patterns of chemokines targeting primarily monocytes and Th1 cells. Unlike cells of epithelial origin, endothelial cells failed to show evidence of proliferation and cell cycle activity that could have indicated their participation in tissue repair processes during the study period (*DNA topoisomerase 2-alpha* (*Top2a*), *Mki67*, *Ube2c*) (Fig. 6c).

### Type 1 effector T cells are efficiently recruited to lungs in SARS-CoV2 infection

**Fig. 7:**
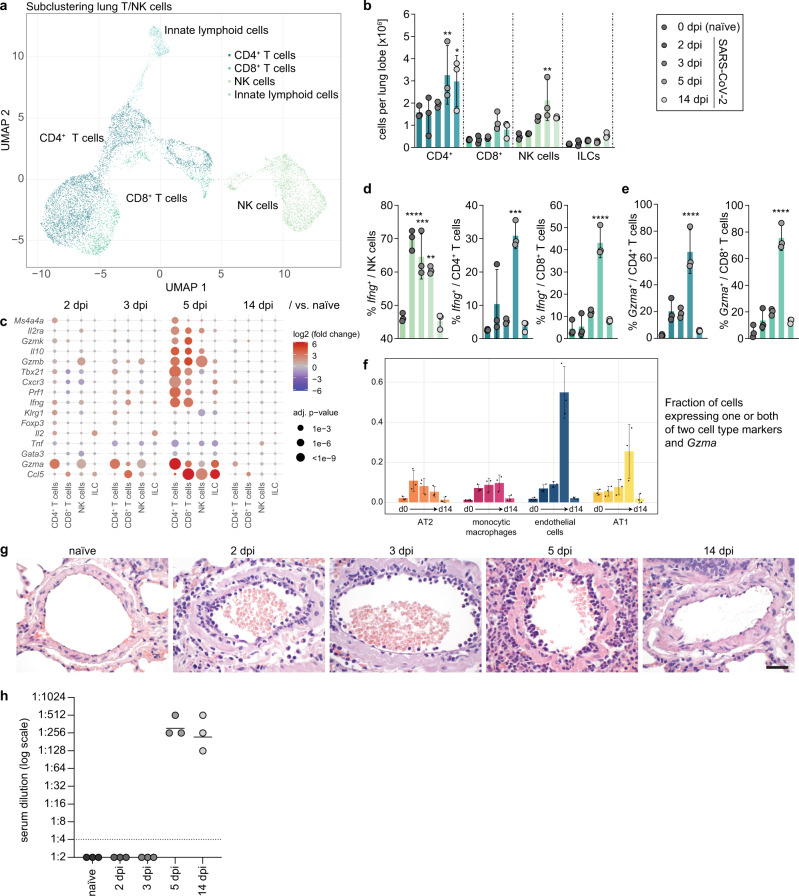
Syrian hamsters exhibit a strong cytotoxic T/NK cell response at day 5 post infection. **a** Uniform manifold approximation and projection (UMAP) plot of lung T/NK cell subclustering indicating cell subpopulations. Clusters defined by Louvain clustering, *n* = 3 per time point. Colors representing individual cell types are depicted in legend. **b** Count of lung T cell subpopulations and NK cells per lung lobe. **c** Dotplot of master regulators of T cell differentiation and effector genes. Coloration and point size indicate log2 fold change and adjusted (adj.) *p-*value, respectively. Adjusted (adj.) *p-*values were calculated by DEseq2 using Benjamini–Hochberg corrections of two-sided Wald test *p-*values. Data from *n* = 3 animals per time point. **d** Fraction of *Ifng*^+^ cells in CD4^+^ T, CD8^+^ T and NK cells at 2, 3, 5, and 14 days post infection (dpi) and naive. **e** Fraction of *Gzma*^+^ cells in CD8^+^ T cells at 2, 3, 5, and 14 dpi and naive. **f** Fraction of AT2 cells, monocytic macrophages, endothelial cells, and AT1 cells containing both at least one cell type marker gene (AT2: *Sftpa1*, *Sftpc*—monocytic macrophages: *Fcn1*, *Saa3*—endothelial cells: *Cldn5*, *Plvap*—AT1: *Ager*, *Aqp5*) together with *Gzma*. Data display means ± SD, *n* = 3 animals per time point. **g** Histopathology of blood vessels at different time points, scale bar for all time points: 50 µm. Micrographs represent *n* = 6 animals per time point pi. **h** Serum neutralization titers of SARS-CoV-2 infected hamsters at 5 and 14 dpi. Serum titers of naive, 2 and 3 dpi did not neutralize up to detection limit 1:4 (dotted line). Data display *n* = 3 animals with mean per time point. **b**, **d**, **e** Bar plots are plotted per cell type in the order: naive, 2 dpi, 3 dpi, 5 dpi, and 14 dpi. (colors fade from dark to light). Data display means ± SD. *n* = 3 animals per time point. Ordinary one-way ANOVA, Šídák’s multiple comparisons test versus corresponding 0 dpi (naive). *P*-values: ^∗^*p* < 0.05, ^∗∗^*p* < 0.01, ^∗∗∗^*p* < 0.001, ^∗∗∗∗^*p* < 0.0001. AT1 and AT2: alveolar epithelial cell type 1 and 2, NK: natural killer cells, ILC: Innate lymphoid cells. d0: day 0 = naive, d14: 14 dpi. Exact *p*-values in order of appearance: **b** CD4: ^∗∗^*p* = 0.0036; ^∗^*p* = 0.0261; NK: **p = 0.0046 **c** IfnγNK: ^∗∗∗∗^*p* < 0.0001; ^∗∗∗^*p* = 0.0003; ^∗∗^*p* = 0.0024; IfnγCD4: ^∗∗∗^*p* = 0.0002; IfnγCD8: ^∗∗∗∗^*p* < 0.0001; GzmaCD4: ^∗∗∗∗^*p* < 0.0001; GzmaCD8: ^∗∗∗∗^*p* < 0.0001.

Our initial cellular analysis of scRNA-Seq data from lung samples had grouped T and NK cells in one set of connected clusters, and we had observed their significant increase in lungs at 5 and 14 dpi (Fig. 1). We hypothesized that cytotoxic immunity might be linked to viral clearance observed at 5 dpi, and elimination by 14 dpi (Supplementary Fig. 2). Therefore, we subclustered NK and T cells to identify 4 subpopulations based on *Cd3e*, *Cd4*, *Cd8a*, and *Natural Killer Cell Granule Protein 7* (*Nkg7)* gene expression (Fig. 7a, Supplementary Fig. 9a), CD4^+^ T cells (*Cd3e*^*+*^*Cd4*^*+*^), CD8^+^ T cells (*Cd3e*^*+*^*Cd8a*^*+*^), NK cells (*Cd3e*^*−*^*Nkg7*^*+*^) and innate lymphoid-(ILC) like cells (*Cd3e*^−^*Cd4*^*−*^*Cd8a*^*−*^*Nkg7*^*−*^). CD4^+^ T, CD8^+^ T and NK cell numbers increased with infection time and peaked at 5 dpi (Fig. 7b). SARS-CoV-2 infection initiated type 1 immunity and cytotoxic effector mechanisms in lungs (Fig. 7c, Supplementary Fig. 9b). The fraction of NK cells expressing interferon gamma (*Ifng*) increased significantly at 2 dpi and peaked at 3 dpi (Fig. 7d, left). In contrast, *Ifng*^+^ effector T cells (*Ifng*^+^ CD4^+^, and CD8^+^ T cells) peaked at 5 dpi (Fig. 7d, right). By 14 dpi, both *Ifng*^+^ NK and T cell responses had declined to naive levels (Fig. 7d).

NK cells and CD8^+^ T cells expressed high levels of cytotoxic genes, but upregulation of *Gzma* was highest in CD4^+^ T cells (Fig. 7c, Supplementary Fig. 9b). Cytotoxic effector function of T cells was evident, 60% of all CD4^+^ T and 70% of all CD8^+^ T cells expressed *Gzma* at 5 dpi (Fig. 7e). Furthermore, we detected cells carrying both a specific cell type marker (for AT1/AT2/endothelial cells, or monocytic macrophages), and simultaneously the cytotoxic cell marker *Gzma*. These possible doublets were absent in naive animals and appeared particularly and reproducibly for endothelial and AT1 cells at the peak of effector T cell recruitment 5 dpi (Fig. 7f), indicating possibly either killer-target interaction or T cell transmigration. The latter notion was supported by the strong immigration of lymphocytes in the endothelium observed in histopathology (Fig. 7g).

Induction of high-affinity neutralizing antibodies is the primary aim of most vaccination strategies against viruses. In the here described primary infection with SARS-CoV-2, neutralizing antibodies were evident by 5 dpi and declined mildly until 14 dpi (Fig. 7h). Matching course of µ heavy chain protein levels measured by proteomics (Ighm, Fig. 2b). Peak of T cells and neutralizing IgM antibodies corresponded with vanishing virus, indicating successful adaptive effector programs.

## Discussion

Detailed understanding of COVID-19 pathophysiology is imperative for the development of therapies to reduce numbers of patients developing lung injury. Notably, recent patient-centered research on COVID-19 was compromised by three blind spots: (i) biomaterial is usually sampled after hospital admission, therefore the early phase of infection and host response has rarely been investigated. (ii) the BAL procedure to access the alveolar compartment is too dangerous for non-intubated patients with COVID-19 pneumonia, so that alveolar host responses can hardly be investigated in mild and moderate COVID-19. (iii) Lung tissue can exclusively be harvested after death in COVID-19 patients, precluding from investigations of non-myeloid alveolar or endothelial host responses in early disease, and enabling for analysis of later disease stadium only in case of fatal outcome. Thus, we performed in-depth analysis of the full course of moderate, self-limited disease that develops in SARS-CoV-2 infected Syrian hamsters. In this model, lungs are fully accessible, providing for detailed analysis of myeloid and non-myeloid compartments including vascular endothelium. Yet, while the immune response is qualitatively similar between human and hamsters in central aspects, the viral dose applied in experimental infections is likely to be higher than in natural aerosol infection. Consequently, infection kinetics, viral decay rate, and immune responses are accelerated^38,39^. In line, we observed the pulmonary peak of viral load, inflammation, and cellular response between 3 and 5 dpi, whereas at 14 dpi the infection was resolved and mechanisms of tissue regeneration were induced. This course of disease confirms observations in other animal models including non-human primates.

Syrian hamsters developed rapid, but moderate, neutrophil recruitment predominantly to bronchi and lung parenchyma, which resolved by 3 dpi. We also observed little of the typical neutrophil-dependent alveolar damage. Although single-cell RNA-sequencing analysis likely underestimates the numbers of this fragile cell type^40^, these findings suggest a minor role for neutrophils. Nonetheless, the neutrophil response is noteworthy, as many other respiratory viruses, e.g., Influenza A Virus and MERS Virus, initiate little or no neutrophil trafficking in rodents^41^. In fact, blood N/L ratios in COVID-19 patients were reported as markers for disease severity, and neutrophil extracellular traps, as well as reactive oxygen species, are suspected to contribute to adverse vascular events^42,43^. Confirming our classification of hamsters as a moderate disease model, N/L ratios were only mildly elevated at 2 dpi, and gene signatures of dysfunctional immunosuppressive neutrophils were observed in severe COVID-19 patients^44^, but not in Syrian hamsters.

In line with COVID-19 patient reports, alveolar macrophage numbers did not decrease in moderate disease settings^25^, and monocytic macrophages were the largest cell population recruited to the lungs, with notable recruitment from 2 dpi on, and peak presence at 5 dpi^27,44^. Monocyte trafficking to lungs was initiated by local expression of CCR2 ligands, and endogenous chemokine expression served as a feedforward loop. Consequently, macrophages were the predominant inflammatory cell type in alveolar spaces as identified by histopathology. Moreover, macrophages presented the earliest and strongest transcriptional response to the infection, primarily responding to intracellular viral RNA with pro-inflammatory cytokines such as CXCL10, CCL2, and others. Notably, the monocytic macrophage pro-inflammatory expression profile was rather productive than dysregulated as it tended more toward effector T cell recruiting chemokines targeting CXCR3 and CCR5, and less toward pro-inflammatory cytokine expression. This matches observations on moderate versus severe courses of COVID-19 in patients and stands in contrast to findings in animal models with severe disease progression such as K18-hACE2 mice^16,25^. It seems unlikely that intracellular viral RNA found in macrophages is the result of active infection of these cells, as they have been demonstrated to be largely resistant to infection ex vivo^45^. scRNA-Seq data obtained from African green monkeys infected with SARS-CoV-2 likewise did not support virus replication in macrophages^46^. Instead, virus uptake may derive from complement receptor and Fc receptor-mediated phagocytosis of complement and antibody-labeled virus, as suggested by neutralizing antibody titers at 5 dpi^47^. Further, we observed early increase in complement factors and IgM µ chain by proteomics analysis in lung tissue. Taken together, the Syrian hamster model endorses the hypothesis that monocyte-derived macrophages are a primary source of the strong pro-inflammatory response observed in COVID-19, yet highlights that their presence not necessarily results in fatal outcome^48^.

Presumably, in the lungs SARS-CoV-2 primarily infects AT2 cells^23^. Of note, only few of them were infected and they reacted with only marginal transcriptional responses, which is probably explained by the recent observation in human lungs that less than 10% of AT2 cells express the crucial entry receptor ACE2^45^. Moreover, coronaviruses are endowed with a multitude of mechanisms that block immunological cascades downstream of interferon signaling and cytoplasmic RNA sensing^49^. Only a small subset of in vitro highly infected cells express pro-inflammatory genes^50,51^. Thus, despite being the primary target for viral replication, epithelial cells were not accountable for early systemic propagation of anti-viral or pro-inflammatory signatures.

Endothelial barrier dysfunction, resulting from endothelial cell stress or death, evokes lung edema and thus contributes to lung failure in severe COVID-19^34,52–54^. However, mechanisms driving endothelial barrier failure in COVID-19 are not well understood, as the endothelial compartment is not accessible in living humans suffering from COVID-19. Autopsy studies reported presence of viral particles in human endothelial cells^34^, but infection of endothelial cells by SARS-CoV has been questioned^55^. In SARS-CoV-2 infected non-human primates viral infection of endothelial cells was not observed^46^. Here, we found that endothelial cells showed a rapid and strong induction of anti-viral response genes, but considering the absence of histopathological evidence for intraendothelial virus, we speculate that virus-positive endothelial cells found by scRNA-Seq were not infected, but were rather an artifact originating from contact with ambient virus or its RNA^2,9^. Concomitant cellular and molecular inflammatory responses in blood suggested that systemic responses were additive to direct, local endothelial cell activation. Notably, this observation is in line with our recent findings that virus-free plasma of COVID-19 patients induced significant endothelial gap formation and loss of junctional VE-cadherin in human endothelial monolayers and lung tissue^33^. Endothelialitis, as observed in autopsies of deceased COVID-19 patients, was also found in infected hamsters^2,9^ and corresponded to their transcriptional pro-inflammatory chemokine responses^34^. Histopathological evidence of pronounced lymphocyte trafficking via capillary endothelial cells also correlated with endothelial *Ccl8* expression.

Lymphocyte recruitment in response to CXCR3 and CCR5 targeting chemokines resulted in the presence of CD4^+^ and CD8^+^ T cells with cytotoxic expression pattern in lungs starting at 3 dpi. Most importantly, viral clearance coincided with appearance of effector T and NK cells stressing their relevance for resolution of SARS-CoV-2 infection and highlighting their importance in vaccination strategies. Studies from other coronaviruses suggest that type 1 immunity is the primary mechanism controlling the infection^56,57^. In severe COVID-19, blood CD4^+^ T, CD8^+^ T, and NK cells expressed markers of exhaustion^58^, a finding not mirrored in moderately sick Syrian hamsters. In contrast, T and NK responses were effective and self-resolving. This matches observations of broad T cell antigen-specificity in the majority of resolved cases independent of mild or severe infection^59^. In our study, we found SARS-CoV-2 neutralizing antibodies at 5 dpi, likely of IgM type, as early appearance and corresponding elevated Ighm protein levels suggested. Seroconversion in COVID-19 patients occurred 7–14 days post diagnosis with IgG titers appearing at later time points^60,61^.

At 14 dpi, infectious virus was no longer detected in hamster lungs, and most transcriptional activity had returned to basal levels. Upregulation of mitotic markers in AT2 cells may reflect regeneration mechanisms after clearing the infection.

The pulmonary capillary microvascular niche in lungs supports alveolar epithelial repair mechanisms following injury, e.g., by secretion of MMP14, VEGF, thrombospondin-1 (THBS1)^62^. Analysis of pulmonary endothelial cell subclusters revealed that bronchial, pulmonary capillary and pulmonary vein endothelial cells showed increased expression of *Thbs1* at 5, but not 14 dpi. Similarly, no increase in *Mmp14* or *Vegf* expression was detected at 14 dpi. In murine influenza characterized by lung injury and pronounced alveolar damage, a pulmonary population of proliferating endothelial cells is present at 14 dpi^63^ that was absent in SARS-CoV-2 infected Syrian hamsters, indicating that the alveolar endothelial and epithelial damage remained moderate in our model. Most notably, lung endothelial cells showed an anti-fibrotic gene signature at 14 dpi (*Nr4a1*^64^, *Akap12*^65^, *Nrarp* (downstream of Notch-signaling))^66^, indicating regeneration rather than repair of lung tissue, thereby matching histopathology findings.

Taken together, we provide evidence that Syrian hamsters recapitulate the course of moderate human SARS-CoV-2 infection. Hamsters displayed nearly prototypic antiviral immune responses starting with rapid, yet self-restricted neutrophilic response, along with a fast and strong monocytic innate immune response following activation after virus uptake, augmenting local anti-viral responses and pro-inflammatory CC chemokine production recruiting a potent type 1 T cell response that probably contributed to elimination of pulmonary residing virus via cytotoxic effector mechanisms. Neutralizing antibodies of IgM type aided in preventing viral spread and fostered cellular virus uptake. Viral infection and inflammatory response in and by lung epithelium is not predominant. Upon successful elimination of virus, alveolar epithelial repair mechanisms started, along with endothelial suppression of fibrotic programs, thus enabling pulmonary regeneration in convalesced hamsters.

Hence, Syrian hamsters represent a highly suitable model to study the pathophysiology of moderate COVID-19, virus-directed and immunomodulatory therapies, and potentially vaccines. SARS-CoV-2 infected Syrian hamsters mount immune responses consistent with COVID-19 patients and enable for detailed investigations on the kinetics and role of specific cell populations, highlighting the dominant contribution of monocytic macrophages, endothelial cells, and T cells to inflammatory responses and resolution of SARS-CoV-2 infection.

Although we present several lines of evidence suggesting a comparable course of disease in Syrian hamsters and moderate COVID-19 in humans, the extent to which results from this animal model can be translated to human patients is limited. This limitation particularly arises from the unavailability of human samples from time points post infection, yet pre symptom onset, as well as lung tissue samples from living human subjects. Due to the lack of an available cell atlas for our model system, we used a combination of marker expression and label transfer from available *Mus musculus* and human datasets for manual curation of lung and blood cell atlases for Syrian hamster. Owing to the limited amount of molecular tools and reagents available for hamsters, such as antibodies, it is not possible to confirm key findings from our transcriptomic analysis e.g., by immunostaining of tissue sections or by ELISAs for cytokines in the blood.

## Methods

### Ethics statement and animal husbandry

All experiments involving animals were approved by institutional and governmental authorities (Freie Universität Berlin and LaGeSo Landesamt für Gesundheit und Soziales Berlin, Germany, permit number G 0086/20). Female and male Syrian hamsters (*Mesocricetus auratus*; breed RjHan:AURA, Janvier Labs, France) were housed in biosafety level 3 (BSL-3) conditions in individually ventilated cages with enrichment. Food and water was provided *ad libidum*. Daily cage temperature and relative humidity measurements ranged from 22–24 **°**C and 40–55%, respectively. Animals were acclimatized for a minimum of 7 days prior to infection.

### Virus stocks

SARS-CoV-2 isolate (BetaCoV/Germany/BavPat1/2020)^67^ was kindly provided by Daniela Niemeyer and Christian Drosten, Charité Berlin, Germany. Virus stocks were propagated under BSL-3 conditions in Vero E6 cells (ATCC CRL-1586). All hamsters described here received virus from the same batch.

### Animal infection

At 10–12 weeks of age hamsters were intranasally infected with 1 × 10^5^ pfu SARS-CoV-2 under anesthesia (0.15 mg/kg medetomidine, 2 mg/kg midazolam and 2.5 mg/kg butorphanol) by applying 60 µL MEM with 1 × 10^5^ pfu SARS-CoV-2 or plain cell culture medium for mock-infected animals. Anesthesia was antagonized with 0.15 mg/kg atipamezole immediately following intranasal application^2^. Clinical signs and weight were monitored daily. Animals with >15% body weight loss over 48 h were euthanized in accordance with animal-use protocols. Euthanasia was performed by cervical dislocation under the anesthesia described above^2^. 1 mL peripheral blood was collected in EDTA-coated syringes. The left lung lobe was collected for histopathology, the right caudal lobe for single-cell analysis, the right cranial lobe for virological assessments and the right medial for bulk RNA as well as proteomics analysis. Experimental design and analysis are summarized in the Supplementary material (Supplementary Fig. 1a). Numbers of animals (*n*) analyzed per read-out and sample type are depicted in Supplementary Fig. 1b. Data for clinical, virological, and transcriptome analysis were derived from two independent experiments (E1 and E2), per experiment *n* = 12 hamsters were infected as described above, per experiment 3 animals from these groups were sacrificed at 2, 3, 5, and 14 dpi to collect samples. For transcriptome analysis (*n* = 3 per time-point) as well as *n* = 3 naive hamsters were used. For the presentation of clinical and virological data, subjects from E1 and E2 were combined (*n* = 6 per time point). For proteome analysis, samples from the same hamsters that were used for transcriptome analysis were employed, additionally, samples from 1 to 4 hamsters and time-point controls (mock-infected, *n* = 3–6 per time point) from an independent third experiment (E3) where animals underwent the exact same infection and treatment were used for proteome analysis to achieve a larger sample size.

### Viral burden assessment

Virus titers were determined by serial dilutions of lung homogenates (50 mg) plated on Vero E6 cells, cells were fixed in 10% formalin, stained with crystal violet (0.75% aqueous solution) and plaques were counted by eye in appropriate dilutions^2^.

For RNA extractions and quantitative RT-PCR, RNA from oropharyngeal swabs, lung tissue, and whole blood was isolated with the innuPREP Virus DNA/RNA Kit (Analytic Jena) according to the manufacturer’s instructions. One-step RT-qPCR reaction with the NEB Luna Universal Probe One-Step RT-qPCR (New England Biolabs) and the 2019-nCoV RT-qPCR primers and probe E_Sarbeco^68^ quantified viral RNA on an Applied Biosystems OneStepPlus qPCR cycler (Thermo Fisher).^2^. Viral RNA copies were calculated per 1 × 10^5^ hamster *Rpl18* transcripts. Primers and probes are listed in the Supplementary Information (Supplementary Table 1).

### Measurement of neutralizing antibodies titer

Serum neutralization tests were performed by two fold serial dilutions (1:4 to 1:512) of complement inactivated (56 **°**C, 2 h) hamster serum plated on sub-confluent monolayers of Vero E6 cells. 50 pfu SARS-CoV-2 were added per well and incubated for 72 h at 37 **°**C, fixed with 10% formalin for 24 h and stained with crystal violet (0.75% aqueous solution). Serum neutralization was considered effective in wells that did not show any cytopathic effect, the highest effective dilution was counted.

### Histopathology and in situ-hybridization of SARS-CoV-2 RNA

For histopathology and in situ-hybridization (ISH), lungs were processed as described^2^. Left lung lobes were immersion-fixed in 10% formalin, pH 7.0, for 48 h, embedded in paraffin, and cut into 2 µm sections. Hematoxylin and eosin (HE) staining and in situ-hybridizations were performed as described^7^ using the ViewRNA™ ISH Tissue Assay Kit (Invitrogen) following the manufacturer’s instructions with minor adjustments. SARS-CoV-2 RNA was localized with probes detecting N gene sequences (NCBI database NC_045512.2, nucleotides 28,274–9533, assay ID: VPNKRHM). An irrelevant probe for detection of pneumolysin was used to control for sequence-specific binding^4^. Amplifier and label probe hybridizations were performed following the manufacturer’s instructions using fast red as chromogen with hemalaun counterstain. Tissues were histopathologically evaluated by board-certified veterinary pathologists (KD, ADG) in a blinded fashion following standardized recommendations^9^, including pneumonia-specific scoring parameters^41^ as described for SARS-CoV-2 infection in hamsters^2^.

### Single cell isolation from whole blood and hamster lungs

Protocols were adapted for BSL-3 facility regulations. For isolation of cells from whole blood, 250 µL blood were lysed in red blood cell lysis buffer (BioLegend), washed and centrifuged according to the manufacturer’s instructions. Resulting RBC-free pellets were resuspended in low-BSA buffer (1× PBS, 0.04% BSA), filtered with 40 µm FloMi filters (Merck) and counted by hemocytometer in trypan blue.

For isolation of single cells caudal lung lobe was removed and placed in storage medium (1× PBS, 0.5% BSA) until further processing. Storage and isolation media contained 2 µg/mL ActinomycinD. Tissues and cells were centrifuged at 350 × *g* for 6 min at 4 °C. Lung lobes were mechanically disassociated with tweezers for 2 min in enzymatic digestion medium containing 3.4 mg/mL Collagenase Cls II (Merck) and 1 mg/mL DNase I (PanReac AppliChem) in 2 mL Dispase medium (Corning) per lung lobe followed by 30 min incubation at 37 °C and 5% CO_2_. After dissociation of digested lung tissue, cell suspensions were pressed through 70 µm cell strainers with plungers. Red blood cells were lysed (BioLegend), washed with an excess of PBS/BSA and resuspended in low-BSA buffer (1× PBS, 0.04% BSA), and filtered with 40 µm low-volume FloMi filters (Merck). Cells were counted in trypan blue.

### Single cell RNA sequencing

Barcoding, cDNA Library generation, and sequencing; filtered cells were adjusted to a final concentration of ~1000 cells/μL in 1× PBS with 0.04% BSA and subjected to partitioning into Gel-Beads-in-Emulsions (GEMs) aiming to recover 6000 single cells per hamster and organ by following the instructions for the Chromium Next GEM Single Cell 3′ GEM, Library & Gel Bead Kit v3.1 (10× Genomics). Resulting single-cell libraries were quantified using Qubit (ThermoFisher) and quality-controlled using the Bioanalyzer system (Agilent). Sequencing was performed on a Novaseq 6000 device (Illumina), with SP or S1 flow cells (read1: 28 nucleotides, read2: 64 nucleotides).

### Bulk RNA sequencing

For lung RNA Bulk Sequencing the medial lung lobe was removed and stored in RNA Later Solution for a maximum of 24 h at 4 °C (ThermoFisher). Lung tissue was homogenized using the TissueLyser II system (Qiagen) and homogenates stored in Trizol reagent (Zymo research). For WBC RNA Bulk Sequencing, white blood cells were isolated as described for scSeq followed by lysis in Trizol reagent. RNA extractions were performed according to the Direct-zol RNA Miniprep protocol (Zymoresearch). Bulk RNA sequencing libraries constructed using the Nebnext Ultra II Directional RNA Library Prep Kit (New England Biolabs), and sequenced on a Nextseq 500 device with read length 76.

### Proteomics sample preprocessing

Lung tissue and serum were added to lysis and inactivation buffer (RIPA) and boiled for 10 min at 95 °C before storage at −80 °C. Samples were thawed on ice, volume was adjusted to 50 µl with water and 25 µl of 50 U benzonase, 50 mM ABC, 2 mM MgCl_2_ added before incubation for 30 min at 37 **°**C. Lysates were processed on a Biomek i7 workstation using the SP3 protocol as previously described with single-step reduction and alkylation^69^. Samples were used for LC-MS/MS analysis without additional conditioning or clean-up.

### Liquid chromatography−mass spectrometry analysis (LC−MS)

High-throughput analysis of serum and lung tissue; Peptide separation has been accomplished in a 5-min water to acetonitrile gradient on an Agilent Infinity II HPLC coupled to a Sciex Triple TOF 6600 mass spectrometer (IonDrive TurboV Source) operating in ScanningSWATH mode with minor changes in the liquid chromatography method^70^. As follows: 5 µg of peptides were loaded and resolved in a linear gradient from 1 to 35% buffer B in 4.5 min before increasing to 40% B in 0.5 min and washing for 0.2 min with 80% buffer B before equilibration for 2.2 min with initial conditions (buffer A: 0.1% formic acid, buffer B: 100% ACN, 0.1% formic acid). For library generation by gas phase fractionation (GPF), 6 single 1 µg injections of pooled serum samples were analyzed by online nanoflow liquid chromatography tandem mass spectrometry on an Ultimate3000 Thermo Scientific Q Exactive Plus Orbitrap, LC-MS instrument (Thermo Fisher Scientific, Waltham, USA). The peptides were concentrated for 3 min on a trap column (PepMap C18, 5 mm × 300 μm × 5 μm, 100 Ǻ, Thermo Fisher Scientific) with a buffer containing 2:98 (v/v) acetonitrile/water containing 0.1% (v/v) trifluoroacetic acid at a flow rate of 20 μl/min. They were separated by a 250 mm LC column (Acclaim PepMap C18, 2 μm; 100 Å; 75 µm, Thermo Fisher Scientific). The mobile phase (A) was 0.1% (v/v) formic acid in water, and (B) 80% acetonitrile in 0.1% (v/v) formic acid. In 155 min total acquisition time gradient B increased in 90 min to 25%, and in 30 min to 40% with a flow rate of 300 nl/min. The MS instrument was operated in the data independent mode as followed: the Orbitrap worked in centroid mode with 4 *m/z* DIA spectra (4 *m/z* precursor isolation windows at 17,500 resolution, AGC target 1e6, maximum inject time 60 ms, 27 NCE). An overlapping window pattern from narrow mass ranges using window placements (i.e., 395–505, 495–605, 595–705, 695–805, 795–805, 895–905 *m/z*) was set. Two precursor spectra, a wide spectrum (395–505 *m/z* at 35,000 resolution) and a narrow spectrum matching the range using an AGC target of 1e6 and a maximum inject time of 60 ms were interspersed every 25 MS/MS spectra at resolution of 17,500. Typical mass spectrometric conditions were as follows: spray voltage, 2.1 kV; no sheath and auxiliary gas flow; heated capillary temperature: 275 °C; normalized HCD collision energy 27%. As lock mass acted the background ion *m/z* 445.1200.

### Computational proteomics

For *Mesocricetus auratus* serum samples, a project specific library was generated by gas-phase fractionation, whereas the lung tissue library was constructed using standard settings in library free mode with DIA-NN (version 1.7.12)^71^. Proteins were annotated either using a protein database generated by translation of the Ensembl 99 annotation of the *M. auratus* genome sequence, or the Uniprot reference proteome (UP000189706). The latter was not used for statistical or functional analysis but is available through PRIDE (PXD025164). The libraries were automatically refined based on the project dataset at 0.01 global *q*-value (using the Generate spectral library option in DIA-NN) as previously described^19^. The output was filtered at 0.01 false discovery rate (FDR) at the peptide level.

### Materials proteomics

Hydrophobic Sera-Mag magnetic carboxylate modified particles (44152105050250 Fisher Scientific), hydrophilic Sera-Mag magnetic carboxylate modified particles (24152105050250 Fisher Scientific), Twintec skirted low bind plates (0030129512 Eppendorf), TCEP (646547 Sigma Aldrich), SDS (A7249.1000 Applichem), CAA (22788 Merck/Millipore), ammonium bicarbonat (/871.2 Roth), 100% ACN (955-212 Fisher Scientific), 80% ethanol (1.00983.2500 Millipore), 230 µl Biomek Tips (B85903 Beckmann Coulter), Eppendorf 500 µl deep well plates (30501101 Eppendorf), Waters Acquity UPLC 700 µl plates (186005837 Waters GmbH) Sequencing grade modified Trypsin (V5117 Promega), Pierce Quantitative Fluorometric Peptide Assay (number 23290), formic acid (85178 Thermo Scientific), water (1.15333.2500 Merck), protease inhibitor cocktail complete mini (Roche 04693124001), benzonase nuclease (Sigma Aldrich E1014-25KU).

### Proteomics data pre-processing

Four serum samples showed low quality and were removed. Peptides with excessive missing values (>30% per group) were excluded from analysis. Batch correction was applied. The peptide matrix was filtered using factor Proteotypic keeping only peptides belonging to one protein group. To obtain a quantitative protein data matrix, the log2-intensities of peptides belonging to one protein group were summarised by maxLFQ method^72^ into protein log intensity.

### Proteomics statistical analysis

Statistical analysis of proteomics data was carried out using internally developed R scripts. Linear modeling was based on the R package LIMMA^73^. Following model was applied to the sets of lung/serum samples (log(p) is log2 transformed expression of a protein): log(p) ~ 0 + Class(Day) + Gender

Here, categorical factor Class(Day) has 8 levels:

Infected(D02), Infected(D03), Infected(D05), Infected(D14),

Control(D02), Control(D03), Control(D05), Control(D14)

Categorical factor Gender has two levels: male, female.

The following contrasts were evaluated to trace time dependence of response to viral infection (Note that Contrast5 addresses the average difference between infected and recovered animals and Contrast6 addresses the difference between infected and control animals on average):

Contrast1: Infected(D02) – Control(D02)

Contrast2: Infected(D03) – Control(D03)

Contrast3: Infected(D05) – Control(D05)

Contrast4: Infected(D14) – Control(D14)

Contrast5: [Infected(D02) + Infected(D03) + Infected(D05)]/3 [Control(D02) + Control(D03) + Control(D05)]/3 − [Infected(D14) − Control(D14)]

Contrast6: [Infected(D02) + Infected(D03) + Infected(D05)]/3 − [Control(D02) + Control(D03) + Control(D05)]/3

In serum set of samples there was only one control group at 3 dpi and it was used to build contrasts replacing control groups at other dpi’s.

For finding regulated features following criteria were applied:

Significance level alpha was set to guarantee false discovery rate below 10% at the response maximum (5 dpi) in both sample types. We found that alpha = 0.01 was delivering regulated proteins with Benjamini–Hochberg FDR below 8% in lung tissue and below 6% in serum and used it for feature selection.

The log fold change criterion was applied to guarantee that the measured signal is above the average noise level. As such we have taken mean residual standard deviation of linear model: log2(T) = mean residual SD of linear modeling (T = 1.45 in lung and T = 1.37 in serum).

### Functional analysis of proteomics data

Functional analysis was carried out using gprofiler2 R package^74^. For selecting the most (de)regulated GO terms we applied filter: 2≤ term size ≤200. Redundancy of terms was then reduced using REVIGO^75^. Default REVIGO settings were applied. Analyses for each Contrast 1–6 and then all in parallel were carried out with Benjamini–Hochberg FDR threshold 0.2. Organism for search was specified as mauratus—*Mesocricetus auratus* (Syrian hamster). Statistical domain scope was set to custom, list of all identified proteins was provided as background.

### Statistical analyses of clinical hamster data

GraphPad Prism 9.1.2 software was used for statistical analysis of clinical data. The statistical details of all analyzed experiments are given in the respective figure legends.

### Annotation of the *M. auratus* genome

The *M. auratus* genome (MesAur1.0) sequence and annotation (gtf file, version 99) was downloaded from Ensembl. We noticed that 3′-UTRs in this annotation were frequently too short to capture all transcriptome reads and particularly the 3′ end reads in single-cell RNA-sequencing, so we extended all 3′-UTRs for coding genes by 1000 bp. The *Ifit2* gene was extended by 2000 bp. For key genes analyzed in this manuscript, we verified that this extension did not lead to overlaps with downstream genes. The details of this approach are depicted in the supplementary note under Elongation of 3′-UTRs in the Ensembl 99 MesAur 1.0 annotation.

The Ensemble annotation was extended by mapping ENSEMBL gene ids without annotated gene names to entrez identifiers and to the homolog associated gene names using biomaRt^76^. Wherever existing, we extracted the gene name from the NCBI’s All_Mammalia.gene_info (download from ftp://ftp.ncbi.nlm.nih.gov/gene/DATA/GENE_INFO/Mammalia/) table, capturing 1067 gene names. Otherwise, we used available homolog associated gene names yielding 1193 additional entries.

### Analysis of bulk RNA-sequencing data

Reads were aligned to the genome using hisat2^77^ and quantified using quasR^78^. We then performed gene set enrichment analysis with tmod^79^ and Hallmark, Reactome and GO BP gene sets from MSigDB v7.0^80^, ranking genes by the product of the sign of the log2 fold change and log10 adjusted *p*-value and converting hamster gene names to human using the biomaRt mouse-to-human mapping.

### Analysis of single-cell RNA-sequencing data

Data analysis was done in R^81^, using Seurat^82^ and packages from tidyverse^83^, and glmer^84^. All used code with annotation is available through Github at https://github.com/Berlin-Hamster-Single-Cell-Consortium.

Raw single-cell sequencing data were processed using CellRanger 3.1.0 (10× Genomics) with standard parameters, based on a combined MesAur1.0/SARS-CoV-2 (GenBank entry MN908947) reference. Raw feature barcode matrices from the CellRanger output were read into Seurat using the Read10X function and a Seurat object created using the CreateSeuratObject function. Cells with more than 7% mitochondrial reads, based on the percentage feature expression of the mitochondrial genes *Cox1*, *Cytb*, *Nd1*, *Nd2*, *Nd4*, *Nd5*, *Nd6* were excluded (reads from other mitochondrial genes were not detected in the data). Furthermore, cells with less than 1000 (lung) or 500 (blood) detected genes were also excluded from downstream analysis. Sample sets (all lung, or all blood, or blood/lung combined from the individual time points) were then integrated using the SCTransform workflow, as illustrated on the Seurat website^85^. Briefly, the Seurat object was split by the hamster that the data points originated from and separately transformed using SCTransform to normalise and scale the data. To prevent batch specific/animal specific effects from obscuring results, these split objects were integrated using the SelectIntegrationFeatures, PrepSCTIntegration, FindIntegrationAnchors, and IntegrateData functions in succession. PCA and UMAP dimensional reduction analyses respectively were performed on the integrated object, using 30 dimensions for the UMAP as the SCT workflow reportedly shows more robust results with higher dimensionality. Cells were subjected to Louvain clustering using the FindNeighbours and FindClusters (with a resolution parameter of 0.8 for lung samples and 0.5 for the blood samples) functions.

As there are currently no publicly available datasets derived from our model system that could be used for fully automated cell type assignment, we used a combination of marker expression and label transfer from available *Mus musculus* and human datasets. To annotate clusters in the lung scRNAseq data, we used Seurat’s TransferData workflow^85^ and two different reference datasets: Tabula Muris^86^ and the Human Lung Cell Atlas^87^. Integration was performed using matching gene names between mouse and hamster, with gene names in the human data converted to mouse using biomaRt. We then used the predicted cell type of the majority of cells in each cluster as well as cell type marker genes from the literature and public databases to guide cluster annotation^88,89^. The following populations were confirmed: Alveolar macrophages (*Siglecf*^+^, *Marco*^+^)^90^, interstitial macrophages (*C1qb*^+^)^91^, monocytic macrophages (*Ccr2*^+^, *Ccr5*^+^, *Arg1*^+^)^92,93^, *Treml4*^*+*^-monocytes (*Treml4*^+^)^94^, neutrophils (*S100a8*^+^, *Cxcr2*^+^, *Camp*^+^)^44,95^, myeloid dendritic cells (mDC) (*Flt3*^+^, *H2-Ab1*^hi^, *Irf8*^lo^, *Tcf4*^lo^) and plasmacytoid dendritic cells (pDC) (*Flt3*^+^, *H2-Ab1*^hi^, *Irf8*^hi^, *Tcf4*^hi^)^86,96–98^, T/NK/cells (*Cd3e*^+^, *Cd4*^+^ or *Cd8a*^+^, *Gzma*^+^, *Nkg7*^+^)^86,95,99–101^, B cells (*Cd79b*^*+*^, *Ms4a1*^*+*^)^44,102^, alveolar epithelial cells type 1 (*Rtkn2*^*+*^)^91^, endothelial cells (*Pecam1*^*+*^)^90^, ciliated epithelial cells (*Foxj1*^*+*^)^102^, alveolar epithelial cells type 2 (*Lamp3*^*+*^)^102^, smooth muscle cells (*Tagln*^*+*^, *Acta2*^*+*^)^91,103^, fibroblasts (*Dcn*^*+*^)^91^ as well as myofibroblasts (*Dcn*^+^, *Tagln*^hi^, *Acta2*^hi^).

For the analysis of single-cell sequencing data for blood samples, we performed initial clustering and identified, cluster marker genes using the FindAllMarkers function. Clusters expressing high levels of erythrocyte marker genes *Snca*, *Fam46c*, and *Alas2* were identified as erythrocyte contamination^104–106^. Cells in these clusters, as well as a cluster of most likely dead cells, marked by the expression of mitochondrial genes, were removed and the data was re-integrated using the workflow described above. Cell type annotations were assigned to the identified cluster using the follow marker genes: Classical (inflammatory) monocytes (*Ccr2*^+^, *Cx3cr1*^lo^, *Adgre1*^+^); non-classical (residential) monocytes (*Ccr2*^-^, *Cx3cr1*^hi^, *Adgre1*^+^)^107–109^; mature neutrophils (*Cxcr2*^+^, *S100a8*^+^, *Camp*^lo^, *Retn*^lo^, *Ltf*^lo^) and immature neutrophils (*Cxcr2*^+^, *S100a8*^+^, *Camp*^hi^, *Retn*^hi^, *Ltf*^hi^)^44,95,110,111^; myeloid dendritic cells (*Flt3*^+^, *H2-Ab1*^hi^, *Irf8*^lo^, *Tcf4*^lo^) and plasmacytoid dendritic cells (*Flt3*^+^, *H2-Ab1*^hi^, *Irf8*^hi^, *Tcf4*^hi^)^86,96–98^; T cells (*Cd3e*^+^, *Cd4*^+^ or *Cd8a*^+^) and activated T cells (*Cd3e*^+^, *Cd4*^+^ and/or *Cd8a*^+^, *Gzma*^+^)^86,95,99,101^, natural killer (NK) cells (*Cd3e*^-^, *Nkg7*^+^)^95,100^; B cells (*Cd79a*^+^, *Ms4a1*^+^)^44,102,112^ and platelets (*Gng11*^+^, *Ppbp*^+^)^22,113^. While cluster 17 showed no expression of *Ccr2*, the levels of *Adgre1* and *Cd14* were considerable and it was considered as comprising classical monocytes for the purposes of this study^114^.

Previously published scRNAseq data of bronchoalveolar lavages originating from COVID-19 patients data from Liao et al.^25^ was processed using the same (Seurat) workflow in R. We kept cells with less than 10% mitochondrial reads, less than 50,000 UMIs and less than 6000 genes and used IntegrateData to combine different samples. We then again used the Human Lung Cell Atlas reference and TransferData to annotate clusters.

Differential cell density was calculated as previously described^115^ by plotting the log2 ratio of two separate 2D kernel density estimators interpolated on the UMAP coordinates of each cell.

Gene ontology and KEGG pathway analysis was performed using the STRING database at string-db.org^116^.

### Reporting summary

Further information on research design is available in the Nature Research Reporting Summary linked to this article.

## Supplementary information

Supplementary Information

Reporting summary

## Data Availability

Raw and processed data is available at the NCBI gene expression omnibus, entry “GSE162208”. The mass spectrometry proteomics data have been deposited to the ProteomeXchange Consortium via the PRIDE partner repository^117^ with the dataset identifier “PXD025164”. Publicly available datasets that were used in this manuscript can be found at “GSE145926”^25^, combined with on sample “GSM3660650”^26^, as well as “doi: 10.6084/m9.figshare.12436517 [https://figshare.com/articles/dataset/COVID-19_severity_correlates_with_airway_epithelium-immune_cell_interactions_identified_by_single-cell_analysis/12436517]”^27^. Source data are provided with this paper.

## Source data

Source data

## Code availability

The code used for data analysis is available at github.com, https://github.com/Berlin-Hamster-Single-Cell-Consortium and via 10.5281/zenodo.4983546^118^ the code is citable. Further Supplementary data is available at http://www.mdc-berlin.de/singlecell-SARSCoV2.
