# Supplementary material for: Temporal omics analysis in Syrian hamsters unravel cellular effector responses to moderate COVID-19
Source: Nat Commun. 2021 Aug 11;12:4869. doi: 10.1038/s41467-021-25030-7 (PMC8357947; doi:10.1038/s41467-021-25030-7)
Supplement: Supplementary file 1 — Supplementary Information [file 41467_2021_25030_MOESM1_ESM.pdf]

Supplementary Information

Temporal omics analysis in Syrian hamsters unravel cellular effector responses to moderate COVID-19

Supplementary Figures, Tables and Notes

Geraldine Nouailles*, Emanuel Wyler*, Peter Pennitz, Dylan Postmus, Daria Vladimirova, Julia Kazmierski, Fabian Pott, Kristina Dietert, Michael Muelleder, Vadim Farztdinov, Benedikt Obermayer, Sandra-Maria Wienhold, Sandro Andreotti, Thomas Hoefler, Birgit Sawitzki, Christian Drosten, Leif E. Sander, Norbert Suttorp, Markus Ralser, Dieter Beule, Achim D. Gruber, Christine Goffinet, Markus Landthaler, Jakob Trimpert* and Martin Witzenrath*

* Corresponding authors:
Correspondence to Geraldine Nouailles (geraldine.nouailles@charite.de), Emanuel Wyler (emanuel.wyler@mdc-berlin.de), Jakob Trimpert (jakob.trimpert@fu-berlin.de), Martin Witzenrath (martin.witzenrath@charite.de)

a

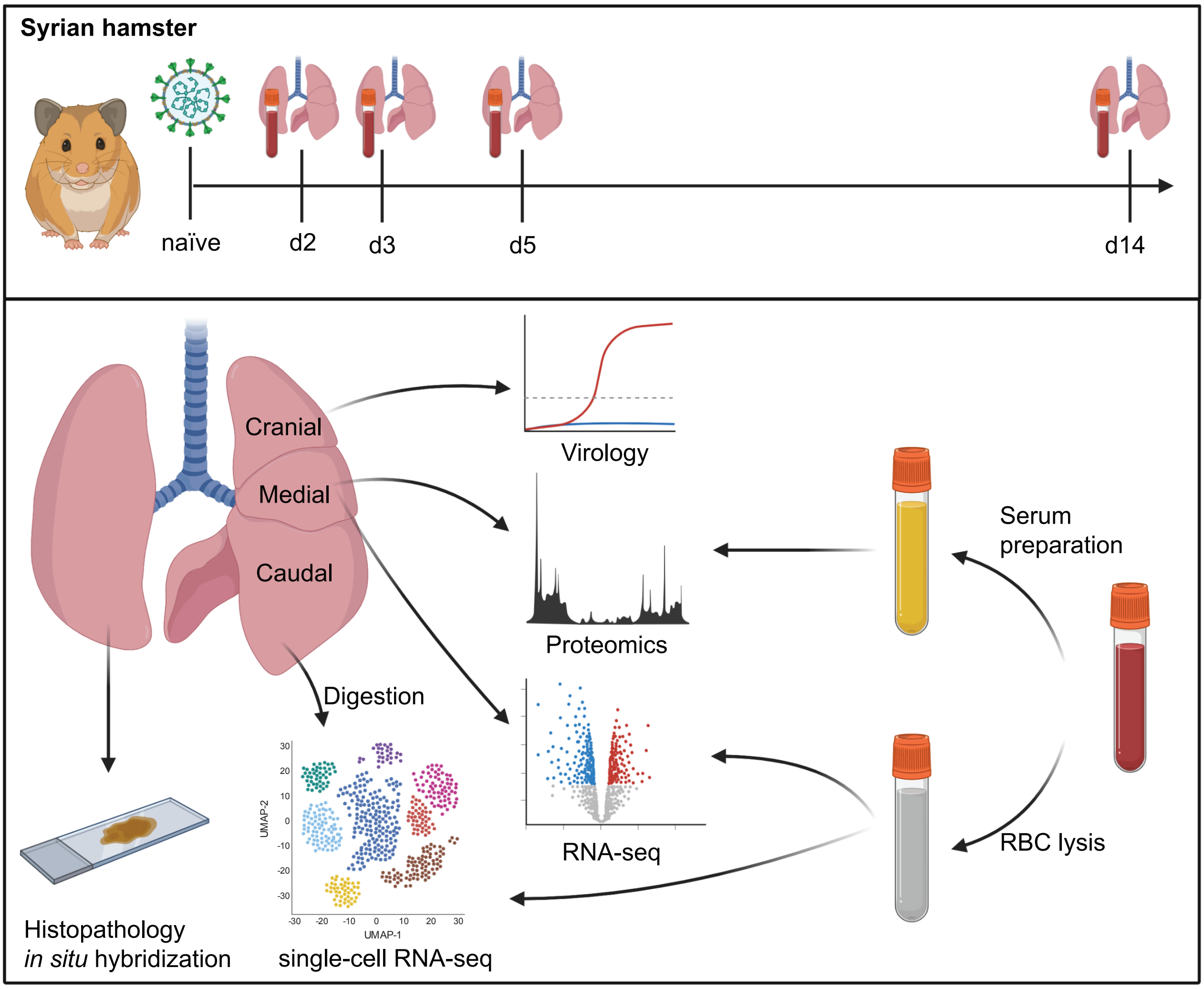

b

lung samples		infected				time-point controls - mock infected			
	**0 dpi (naive)**	**2 dpi**	**3 dpi**	**5 dpi**	**14 dpi**	**2 dpi**	**3 dpi**	**5 dpi**	**14 dpi**
**clinics/virology**		6	6	6	6	0	0	0	0
**transcriptomics**	3	3	3	3	3	0	0	0	0
**proteome**		6	5	6	5	3	3	3	3

serum/blood		infected				time-point controls - mock infected			
	**0 dpi (naive)**	**2 dpi**	**3 dpi**	**5 dpi**	**14 dpi**	**2 dpi**	**3 dpi**	**5 dpi**	**14 dpi**
**clinics/virology**		6	6	6	6	0	0	0	0
**transcriptomics**	3	3	3	3	3	0	0	0	0
**proteome**		6	4	7	6		6		

Supplementary Fig. 1: Experimental Design and animal numbers per analysis and time point. (a), Depicted experimental design of the study (generated with BioRender.com). (b), animal numbers per analysis type and analysis time point in the study. d2: day 2 = 2 days post infection (dpi), RNA-Seq: bulk RNA sequencing analysis, single-cell RNA Seq: single-cell RNA sequencing analysis; RBC: red blood cells.

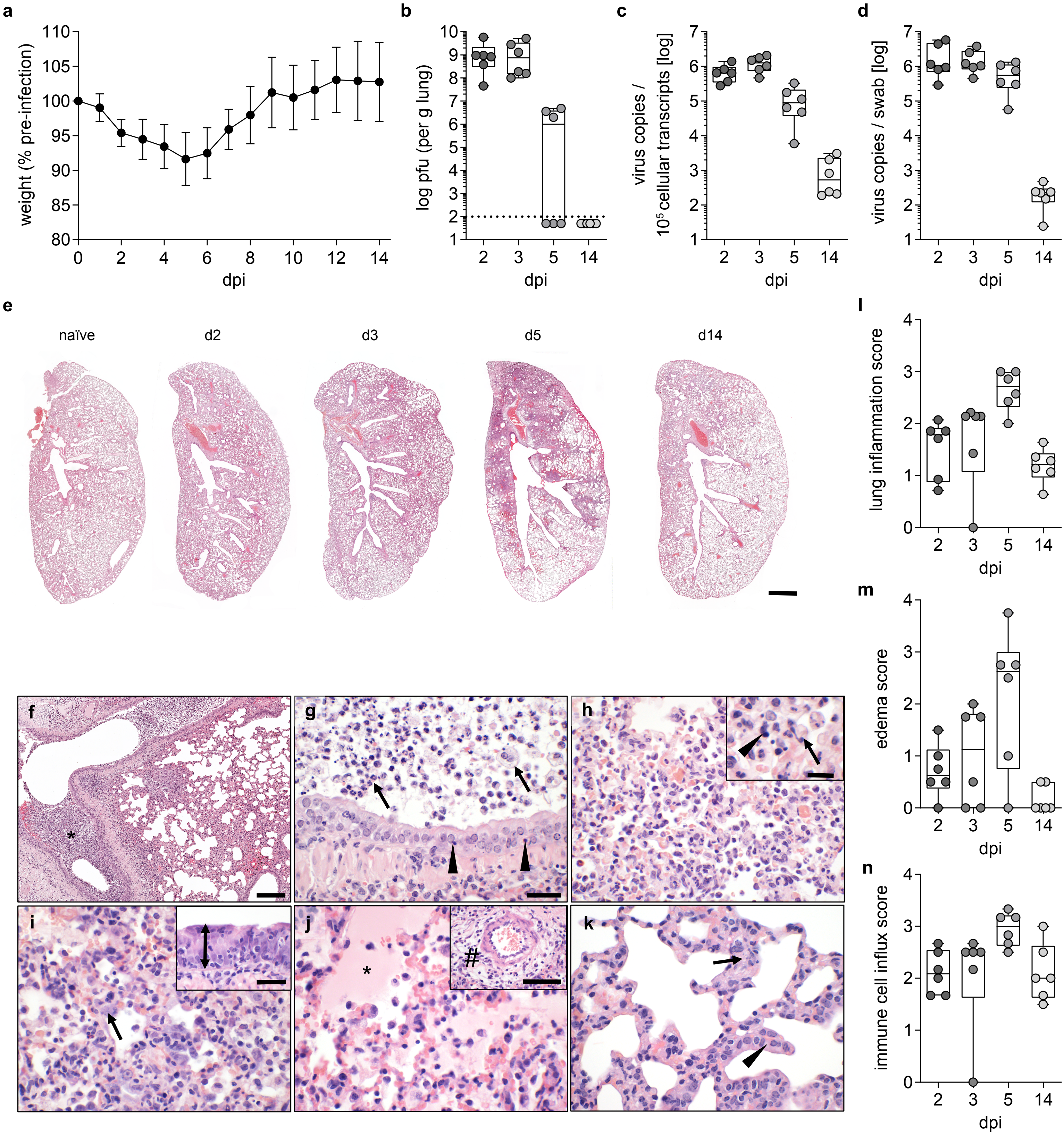

Supplementary Fig. 2: Course of SARS-CoV-2 infection in Syrian hamsters. (a), Relative body weight curve of hamsters recorded up to 14 days post infection (dpi). n = 6 per analysis time point summarized for body weight monitoring over time until analysis time point (n = 24 up to 2dpi, n = 18 up to 3 dpi, n = 12 up to 5 dpi, n = 6 up to 14 dpi).(b), Box plots of virus titers in lung homogenates titrated on Vero E6 cells. Dotted line represents limit of detection. (c), SARS-CoV-2 genomic RNA copies in lung tissue per 105 cellular *Rpl18* transcripts and (d), RNA copies per bucco-pharyngeal swab. (e), whole cross sectional scans of left lung lobes of naïve (left) or SARS-CoV-2 infected hamsters at 2 to 14 dpi. Bar: 1 mm. (f-k), Histopathology at higher magnifications (representative images of haematoxylin and eosin stains) identified suppurative bronchitis at 2 dpi (f, asterisks) with intraluminal neutrophils (g, arrows) and early bronchointerstitial pneumonia with necrosis of bronchial epithelial cells at 3 dpi (g, arrowheads). At 3 dpi, high numbers of neutrophils and macrophages in alveolar lumina of peripheral lung parenchyma (h; inset: neutrophils, arrowhead and macrophages, arrow). At 5 dpi (i), stronger immune cell influxes accompanied by hyperplasia of alveolar (arrows) and bronchial epithelial cells (inset, double headed arrow). Alveolar (j, asterisk) and perivascular edema (j inset, hash) at 5 dpi. (k) At 14 dpi, inflammation still present albeit weaker, hyperplasia of alveolar epithelial cells, clearance of alveolar edema and re-ventilation of alveolar lumina. Scale bars: f = 200 µm, g – k = 50 µm, inset h = 20 µm, inset i = 50 µm, inset j = 100 µm. Micrographs represent n = 6 animals per time point pi. (l - n) Box plots of histopathology scores: parameters were classified (0 absent – 4 severe) and summarized as published 1, 2 with modifications: (l), lung inflammation score including: severity of pulmonary inflammation, bronchitis, bronchial necrosis, alveolar necrosis, hyperplasia of alveolar epithelial cells type II, bronchial epithelial hyperplasia and endothelitis. (m), edema score: alveolar and perivascular edema. (n), immune cell influx: neutrophils, macrophages and lymphocytes. (a) Mean ± standard deviation (SD), (b – d) and (l – n), Box plots, the middle line in the boxplot displays the median, the box indicates the first and third quartile, whiskers the minimum to maximum., n = 6 per time point.

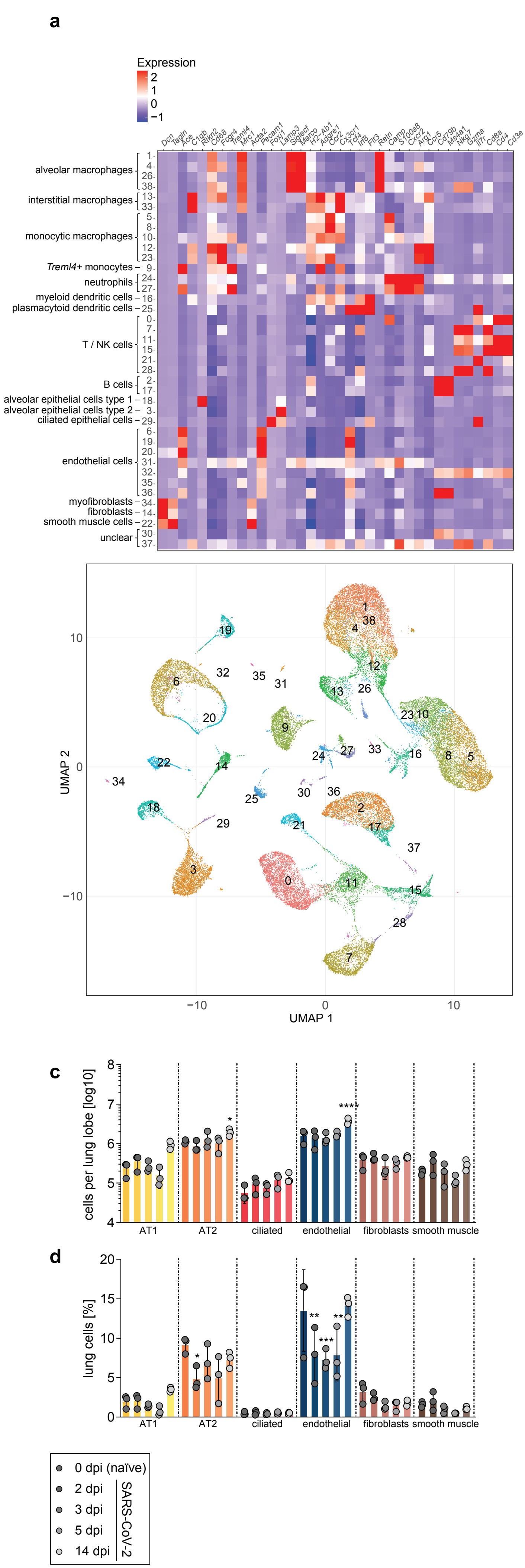

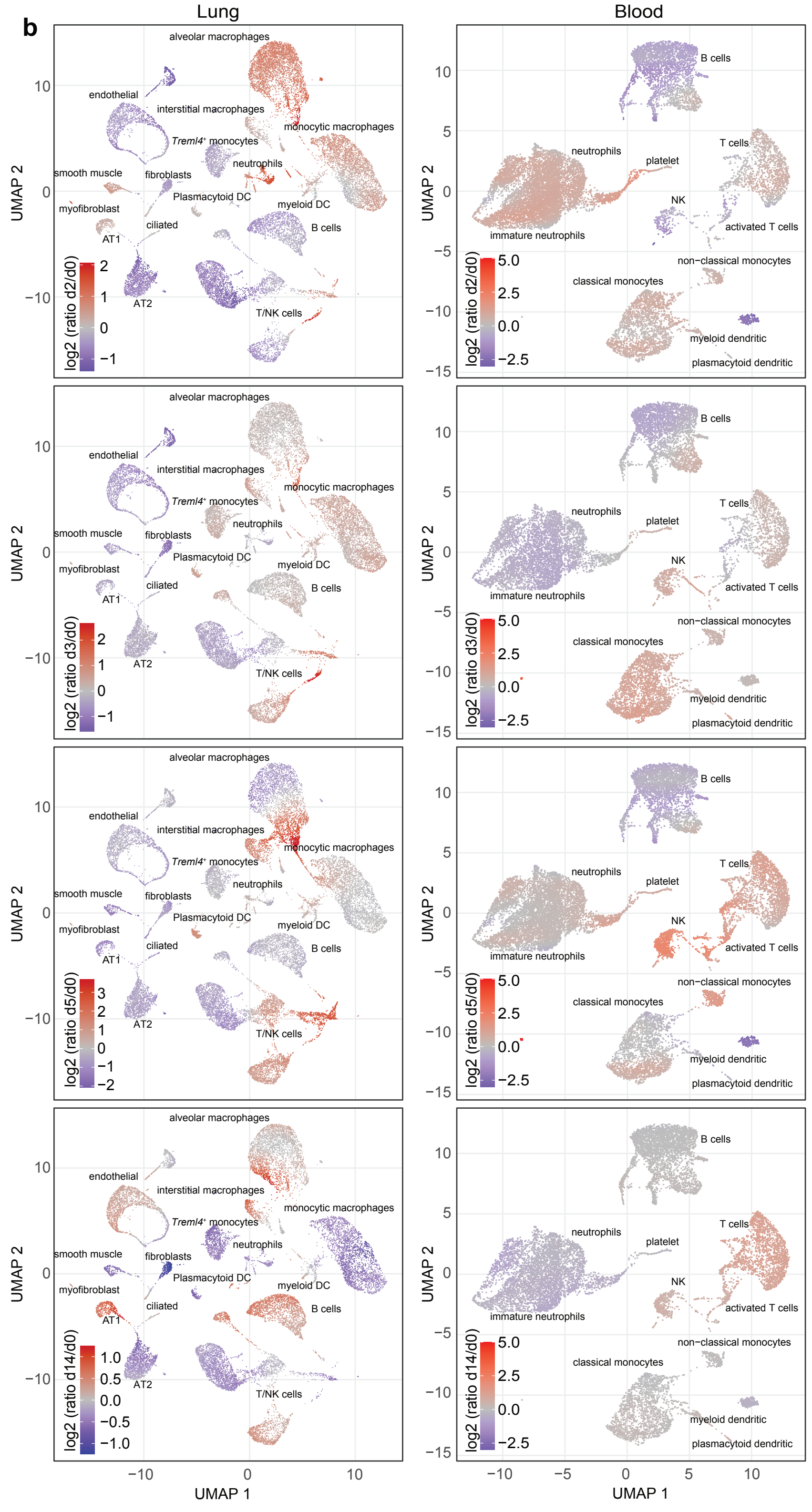

Supplementary Fig. 3: Transient leukopenia induced by SARS-CoV-2 infection in blood of hamsters. (a), Heatmap of cell type-marker gene expression in identified clusters for lung cells, based on classical cell type-markers, top. Normalized average gene expression levels for cells in a cluster are indicated by coloration. Uniform manifold approximation and projection (UMAP) indicates identified clusters and their numbering, bottom. (b), Changes in cellular component density in UMAP projection over the infection time course. Coloration indicates the log2 fold change for each time point (2, 3, 5 and 14 days post infection (dpi)), relative to control groups (naïve). (c and d), Number and percentage of cellular components for each time point pi per lung lobe. *, $p < 0.05$; **, $p < 0.01$; ***, $p < 0.001$; ****, $p < 0.0001$. AT1 and AT2: alveolar epithelial cell type 1 and 2, DC: dendritic cells, NK, natural killer cells; d0: day 0 = naïve, d14: 14 dpi. Exact *p*-values in order of appearance: (c) AT2: *, $p = 0.0334$; endothelial: ****, $p < 0.0001$; (d) AT2: *, $p = 0.0418$; endothelial: **, $p = 0.0016$; ***, $p = 0.0004$; **, $p = 0.0017$.

e

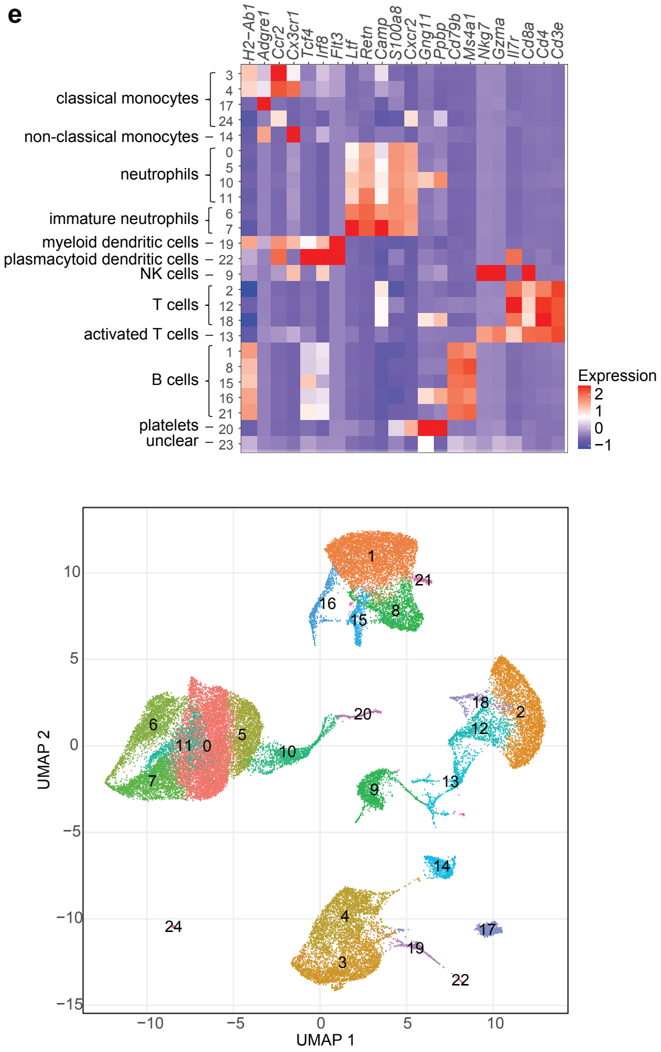

f

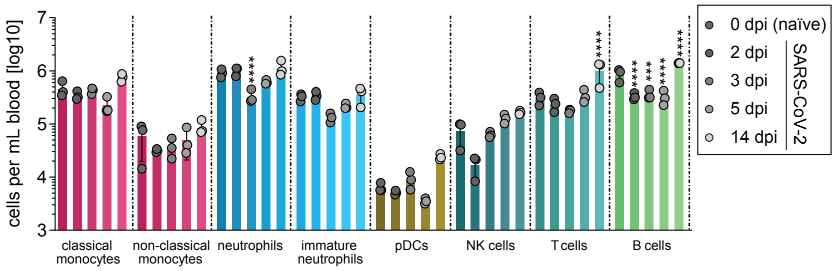

Supplementary Fig. 3 (continued): Transient leukopenia induced by SARS-CoV-2 infection in blood of hamsters. (e), Heatmap of cell type-marker gene expression in identified clusters for blood cells, based on classical cell type-markers. Normalized average gene expression levels for cells in a cluster are indicated by coloration. (f), Number of cells for each time point pi per mL blood. (a, b and e), Clusters defined by Louvain clustering, n = 3 per time point. (c, d and f), Bar plots are plotted per cell type in the order: naïve, 2 dpi, 3 dpi, 5, dpi and 14 dpi. (Colors fade from dark to light). Data display means ± SD. n = 3 animals per time point. Ordinary one-way ANOVA, Šídák's multiple comparisons test versus corresponding 0 dpi (naïve). *, $p < 0.05$; **, $p < 0.01$; ***, $p < 0.001$; ****, $p < 0.0001$. AT1 and AT2: alveolar epithelial cell type 1 and 2, DC: dendritic cells, NK, natural killer cells; d0: day 0 = naïve, d14: 14 dpi.
Exact *p*-values in order of appearance: (f) neutrophils: ****, $p < 0.0001$; T cells: ****, $p < 0.0001$; B cells: ****, $p < 0.0001$; ***, $p = 0.0001$; ****, $p < 0.0001$.

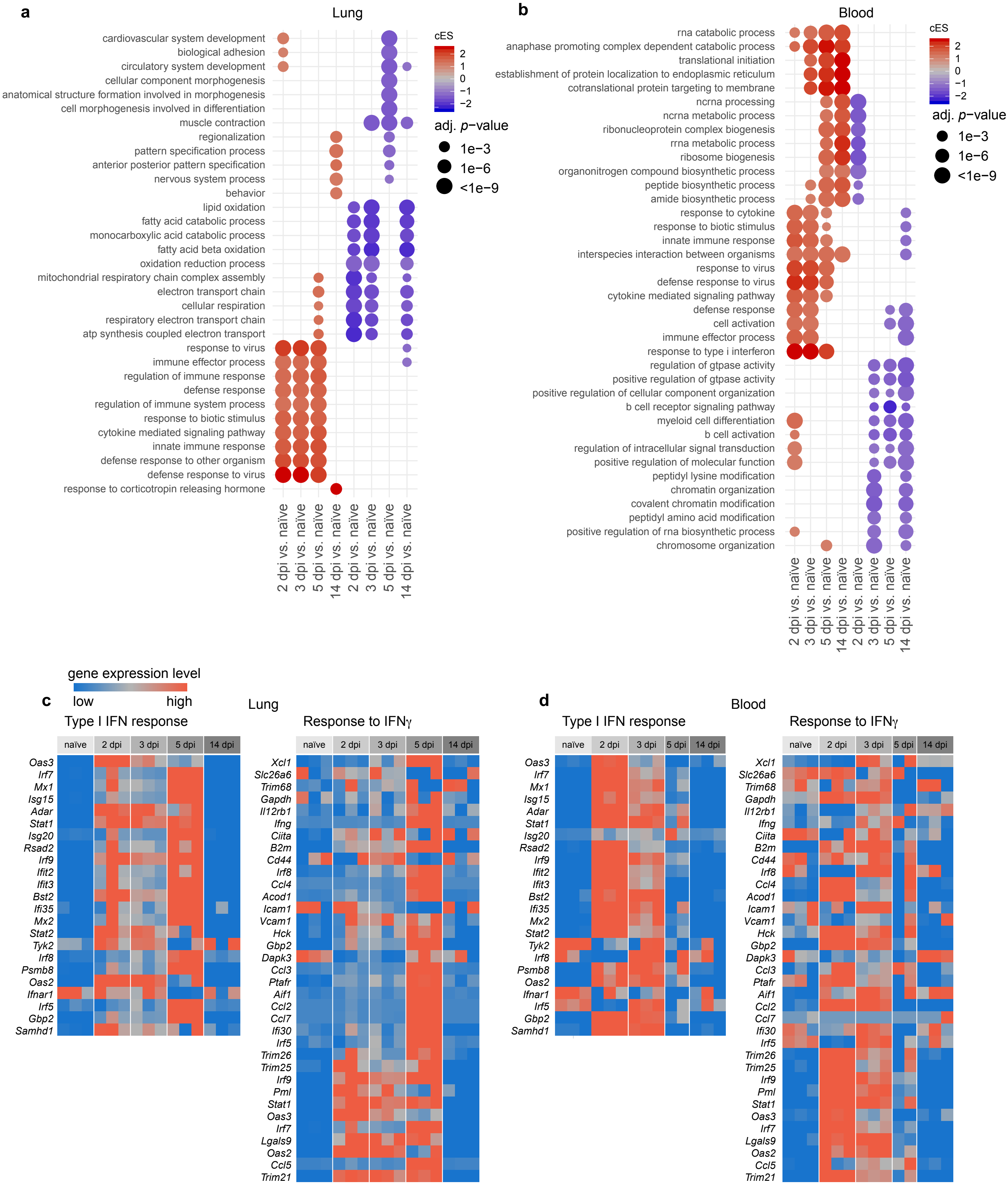

Supplementary Fig. 4: Interferon signaling in lungs and blood induced in hamsters after SARS-CoV-2 infection. (a), Dotplot of enriched terms from hallmark, reactome and gene ontology (biological process) gene sets in lung bulk RNA-sequencing samples over the infection time course compared to naïve animals. Coloration and point size indicate effect size (cES) and adjusted (adj.) *p*-value for each time point (2, 3, 5 and 14 days post infection, (dpi)), relative to control groups (naïve), as calculated by tmod using Benjamini-Hochberg correction on a two-sided CERNO test. n = 3 per time point. (b), as in (a) but for blood samples. naïve, 2, 3, and 14 dpi: n = 3, 5 dpi: n = 2. (c), Heatmap of interferon type I and interferon gamma related genes as in 3 in lungs over the infection time course. Normalized gene expression levels (z-scores) are indicated by coloration for each animal. (d), as in (c), but for blood samples. n = 2 for blood samples of day 5 pi, n = 3 for all other time points.

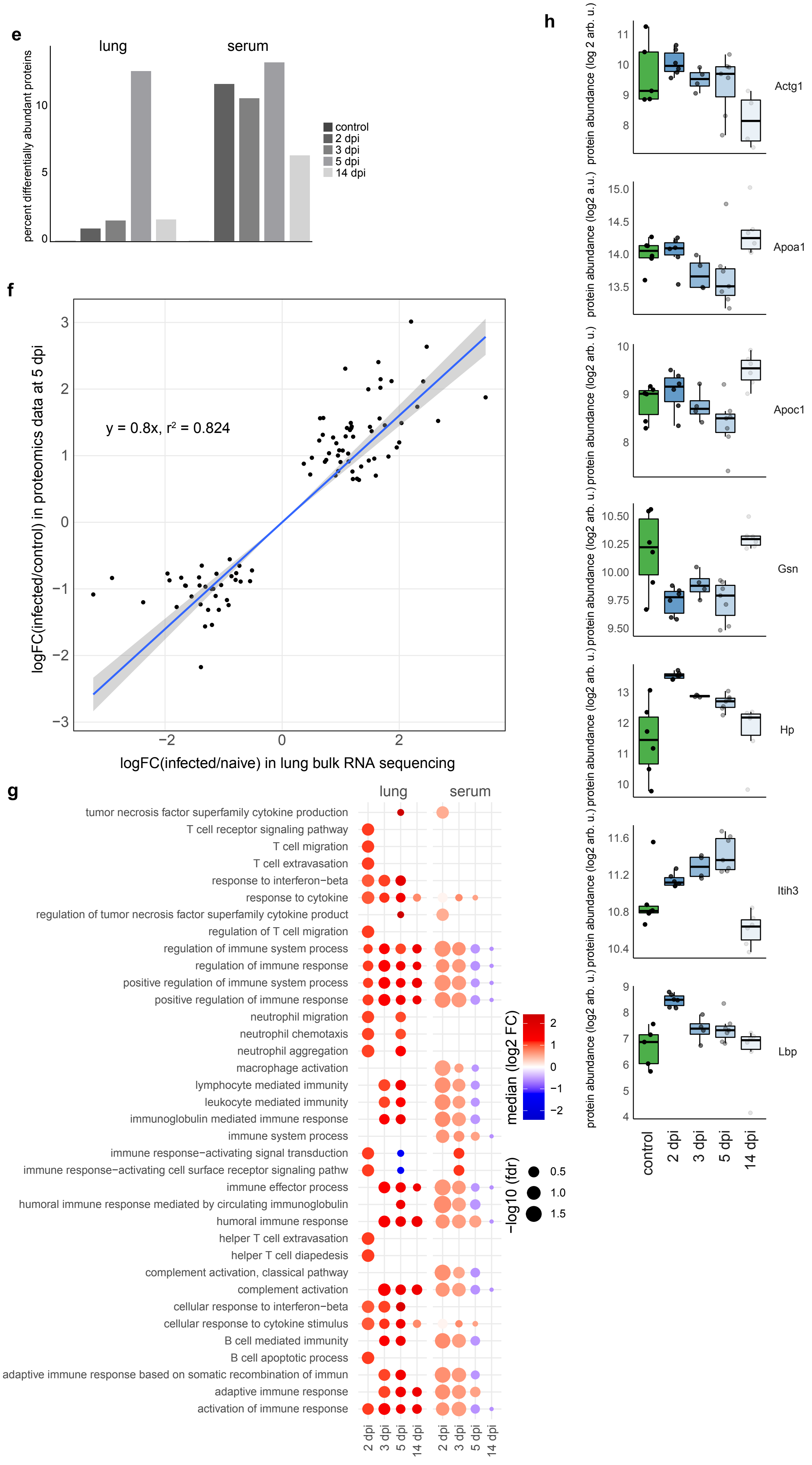

Supplementary Fig. 4 (continued): Interferon signaling in lungs and blood induced in hamsters after SARS-CoV-2 infection. (e) Magnitude of host response over time as fraction (in %) of differentially regulated proteins (*p*-values < 0.01). (f), Correspondence of differentially expressed genes in proteomic and bulk RNA sequencing data was explored by linear regression (intersection at 0) of log transformed fold changes of infected vs control (5 dpi, *p*-value < 0.01). Linear fit (blue line) showed almost ideal consistency between regulations (slope = 0.8) and a correlation coefficient r = 0.9 ($r^2 = 0.82$). Standard error of the estimate is shown as the light grey stripe surrounding the blue line. (g) Temporal evolution of gene ontology/biological process terms connected with immune system response in lung tissue (left part) and in serum (right part), for the indicated time points compared to samples from uninfected animals. Enriched terms were filtered for terms mentioning “immune”, “complement”, “cytokine”, “interferon”, “neutrophil”, “macrophage”, “T cell” and “B cell” and attained false discovery rate (fdr) below 0.2 at least at one dpi in lung or serum. Size of dots correspond to the inverse of the fdr, color corresponds to median $\log_2$(fold change (FC)) of proteins, contributing to the term. (h) As in Fig. 2b but only serum proteins previously reported in human studies. Box plots, the middle line in the boxplot displays the median, the box indicates the first and third quartile, whiskers the 1.5 interquartile range (IQR). Serum sample group sizes: control: n = 6, 2dpi: n = 6, 3dpi: n = 4, 5dpi: n = 7, 14 dpi: n = 6. All non-missing values are shown. Arb. u.: arbitrary units.

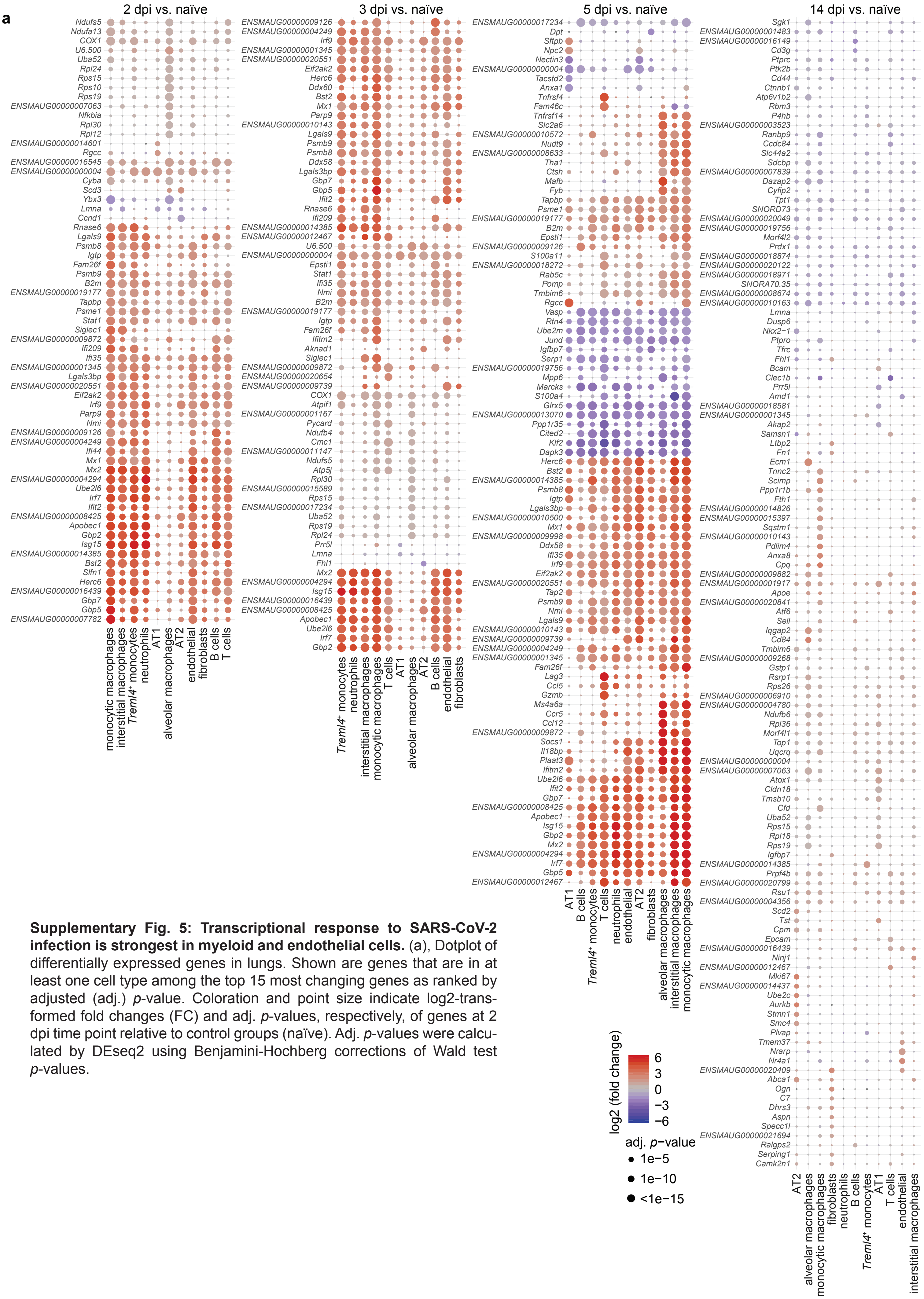

Supplementary Fig. 5: Transcriptional response to SARS-CoV-2 infection is strongest in myeloid and endothelial cells. (a), Dotplot of differentially expressed genes in lungs. Shown are genes that are in at least one cell type among the top 15 most changing genes as ranked by adjusted (adj.) *p*-value. Coloration and point size indicate log2-transformed fold changes (FC) and adj. *p*-values, respectively, of genes at 2 dpi time point relative to control groups (naïve). Adj. *p*-values were calculated by DEseq2 using Benjamini-Hochberg corrections of Wald test *p*-values.

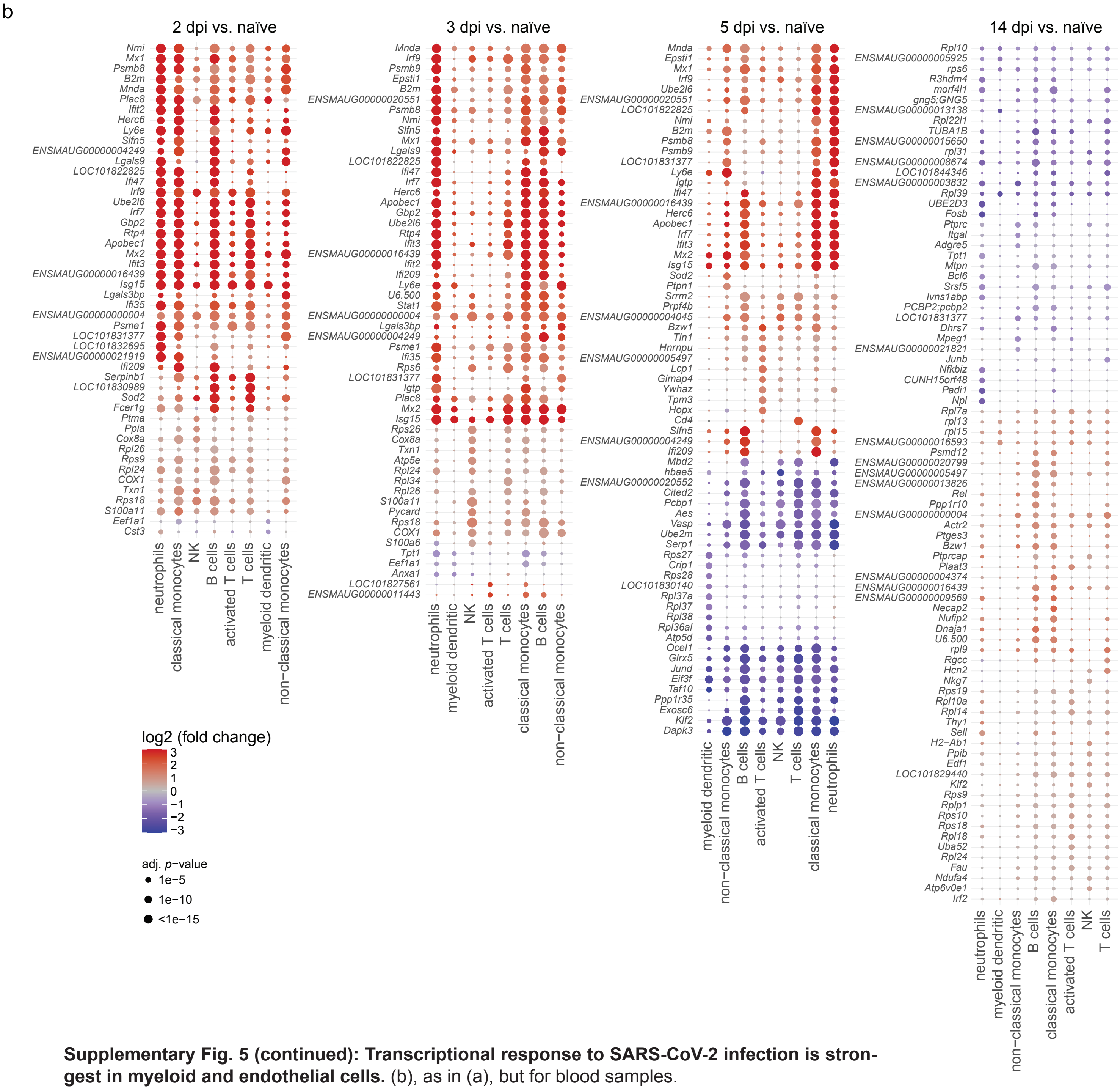

Supplementary Fig. 5 (continued): Transcriptional response to SARS-CoV-2 infection is strongest in myeloid and endothelial cells. (b), as in (a), but for blood samples.

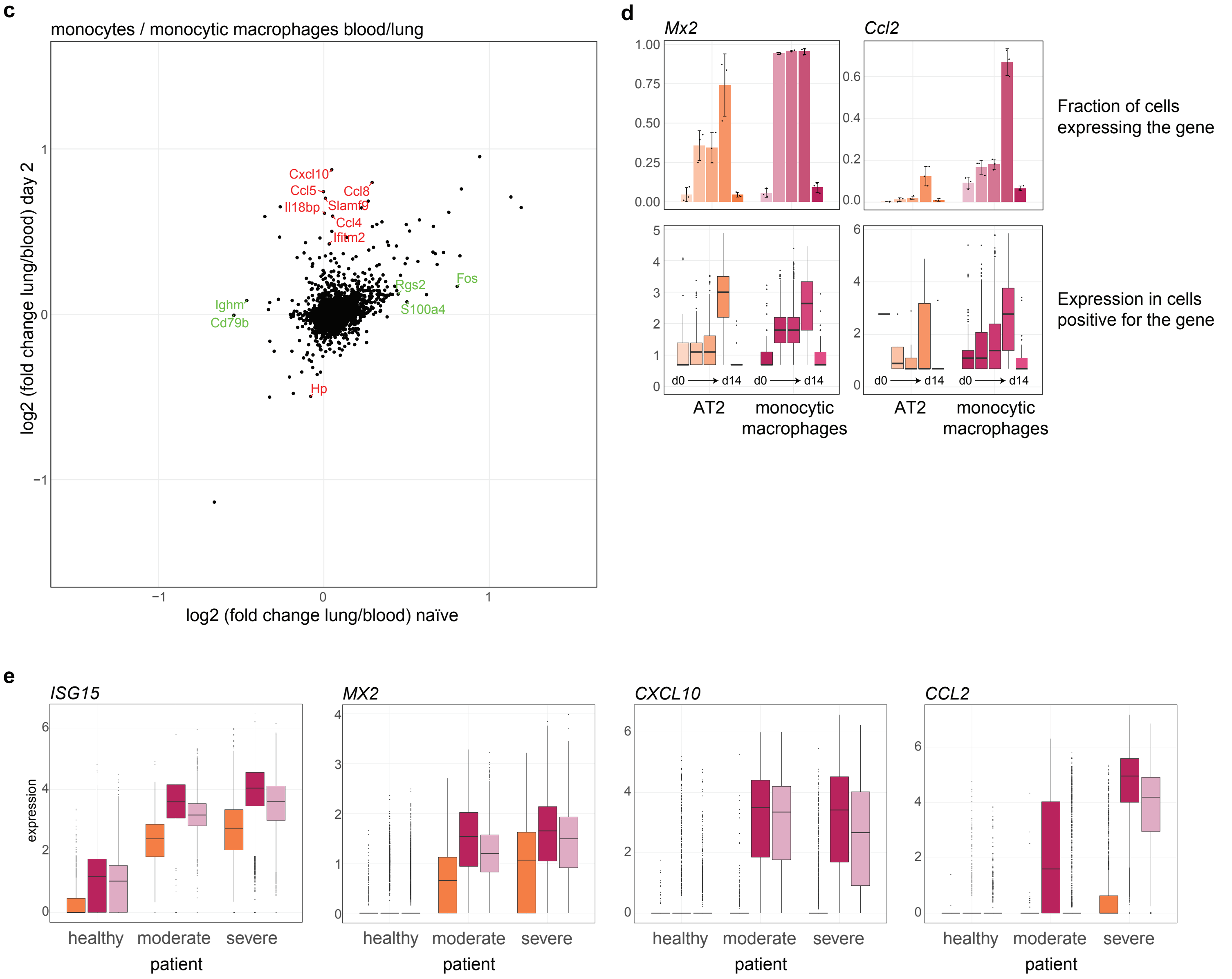

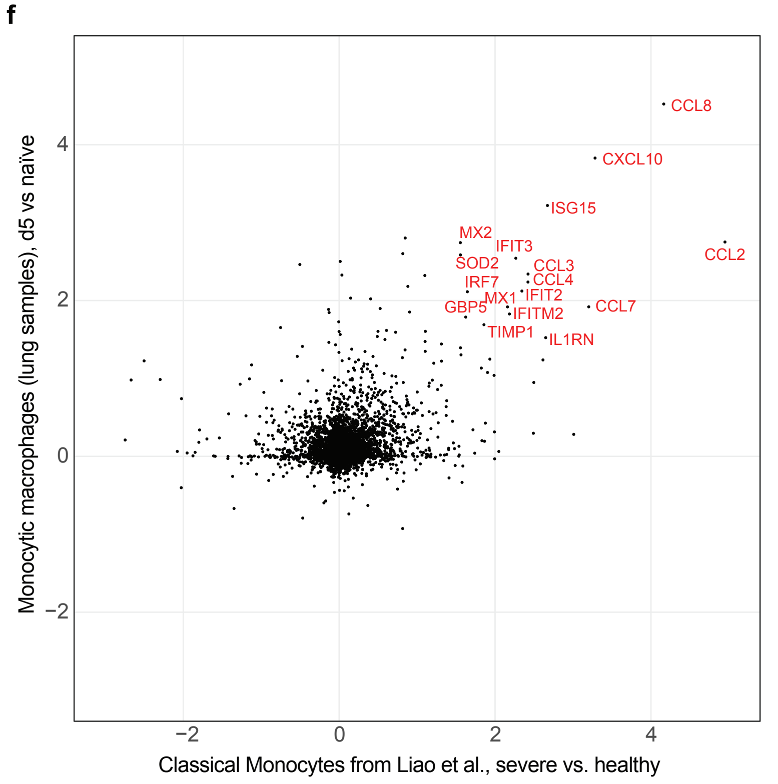

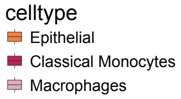

Supplementary Fig. 5 (continued): Transcriptional response to SARS-CoV-2 infection is strongest in myeloid and endothelial cells. (c), Scatter plot showing differential expression in monocytes / monocytic macrophages of lungs compared to blood. Plotted are log(2) transformed fold changes (FC) from naïve (horizontal axis) and 2 day infected (vertical axis) animals. Outliers deregulated only in lungs are shown in red, outliers deregulated only in the blood in green. (d), Top, fraction of AT2 and monocytic macrophages from all time points in lung samples expressing *Mx2* and *Cxcl10*. Bottom, boxplots of gene expression values of the cells positive for *Mx2* (left) and *Cxcl10* (right). Data derived from n = 3 animals per time point. (e), Boxplots show expression of, from left to right, *ISG15*, *MX2*, *CXCL10* and *CCL2* in patient BAL data from 4 in the indicated cell types. Patient categories (healthy n = 4, moderate n = 3 and severe n = 6) according to the original publication. (f), Scatter plot of log2-transformed fold changes of gene expression values of classical monocytes in BAL data from 4 (severe COVID-19 patients compared to healthy individuals, horizontal axis), and monocytic macrophages from hamsters (5 dpi compared to naïve, vertical axis). For barplots, data display means ± SD. n = 3 per time point. For box plots, the middle line in the boxplot displays the median, the box indicates the first and third quartile, whiskers the 1.5 interquartile range (IQR). Outliers beyond are marked by single dots. AT2: alveolar epithelial cells type 2, d0: day 0 = naïve, d14: 14 dpi.

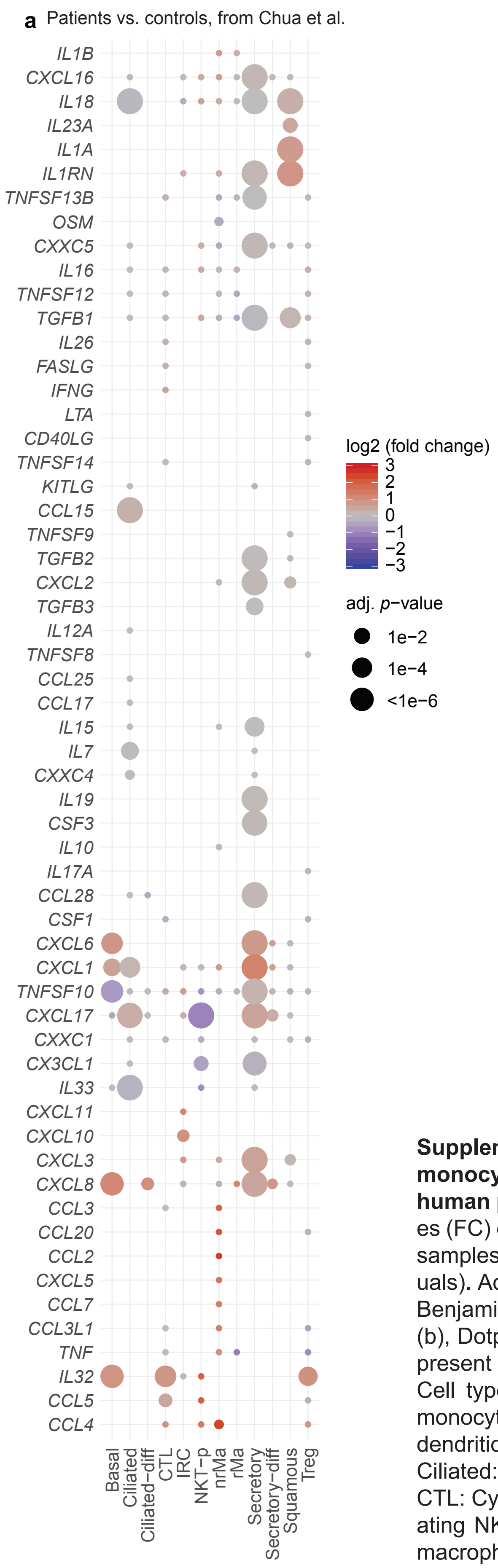

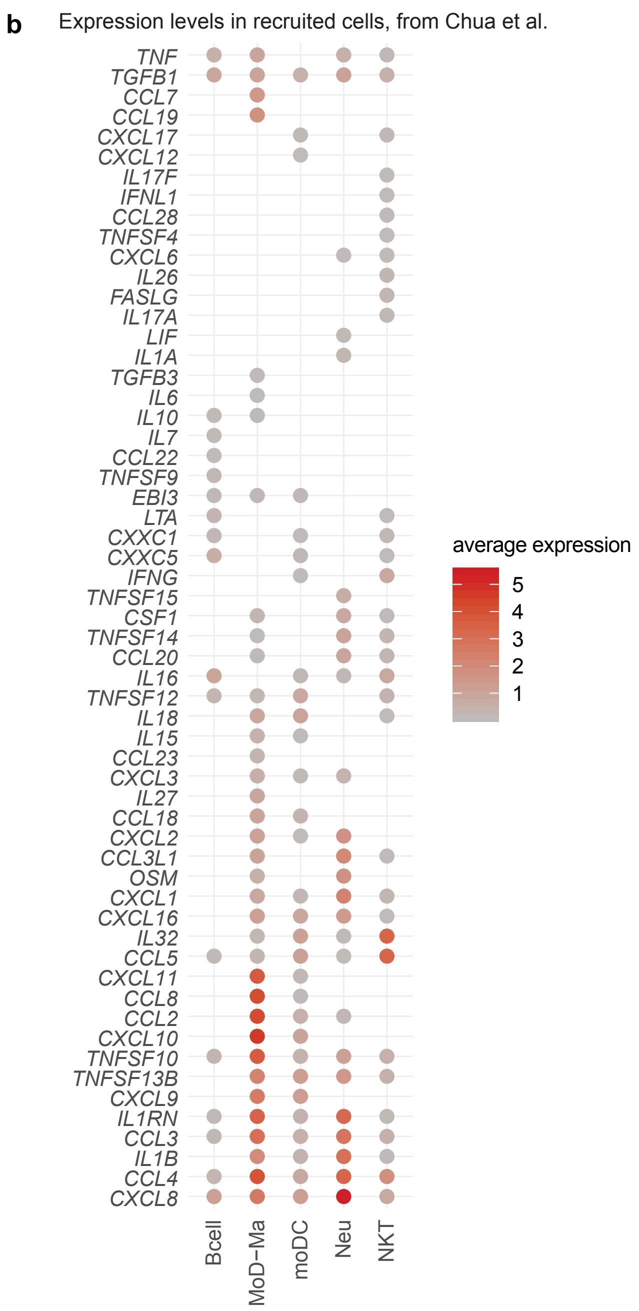

Supplementary Fig. 6: Strong transcriptional response in monocytic macrophages from hamsters is recapitulated in human patient data. (a), Dotplot of log2-transformed fold changes (FC) of gene expression values in cell types from upper airway samples from 5 (COVID-19 patients compared to healthy individuals). Adjusted (adj.) *p*-values were calculated by DEseq2 using Benjamini-Hochberg corrections of two-sided Wald test *p*-values. (b), Dotplot of average gene expression values in cell types only present in COVID-19 patients from upper airway samples from 5. Cell types are defined as following – Bcell: B cells; MoD-Ma: monocyte-derived macrophage; moDC: monocyte-derived dendritic cell; Neu: Neutrophil; NKT: NKT cell; Basal: basal cells; Ciliated: ciliated cells; Ciliated-diff: Differentiating ciliated cells; CTL: Cytotoxic T cell; IRC: IFNG responsive cell; NKT-p: Proliferating NKT cell; nrMa: Non-resident macrophage; rMa: Resident macrophage; Secretory: secretory cells; Secretory-diff: differentiating decretory cells; Squamous: squamous cells; Treg: Regulatory T cell.

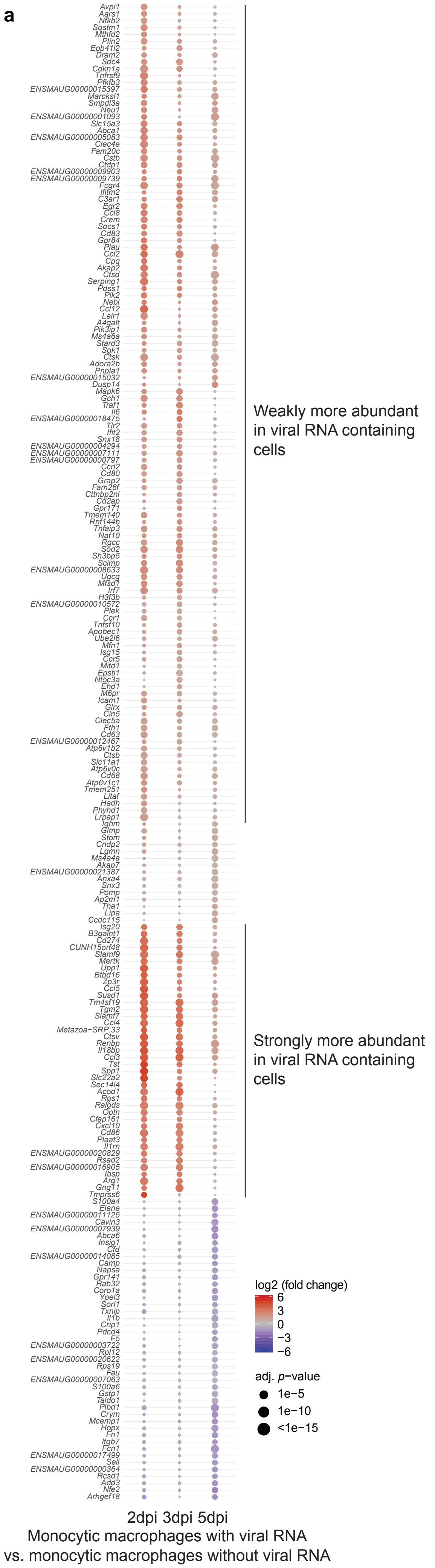

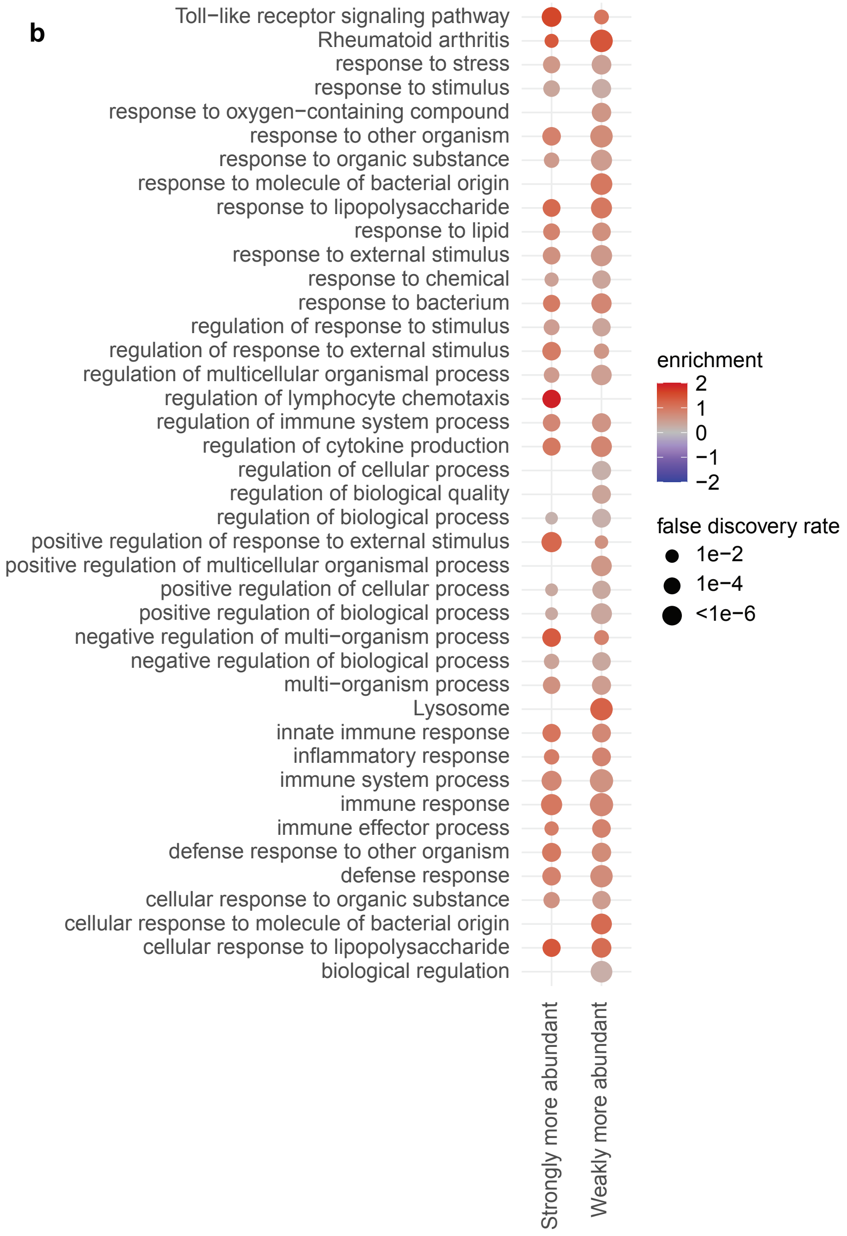

Supplementary Fig. 7: TLR signaling is activated in monocytic macrophages containing viral RNA. (a), Dotplot of differentially expressed inflammatory mediators of monocytic macrophages containing viral RNA compared to monocytic macrophages not containing viral RNA. Coloration and point size indicate log2 fold change and adjusted (adj.) *p*-value of genes at each time point (2, 3 and 5 days post infection (dpi)) of cells with viral RNA compared to those without. (b), GO and KEGG pathway term enrichment of the gene sets defined in (a). The enrichment is computed by a hypergeometric test and the false discovery rate using the Benjamini-Hochberg procedure.

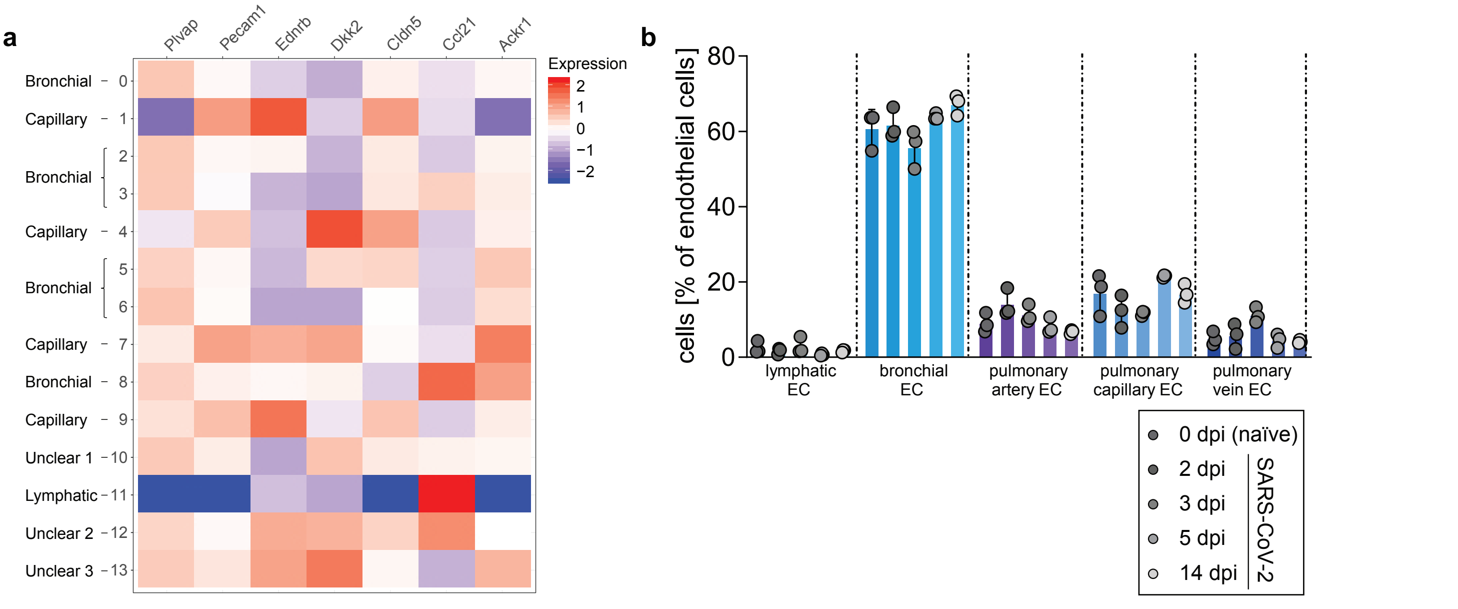

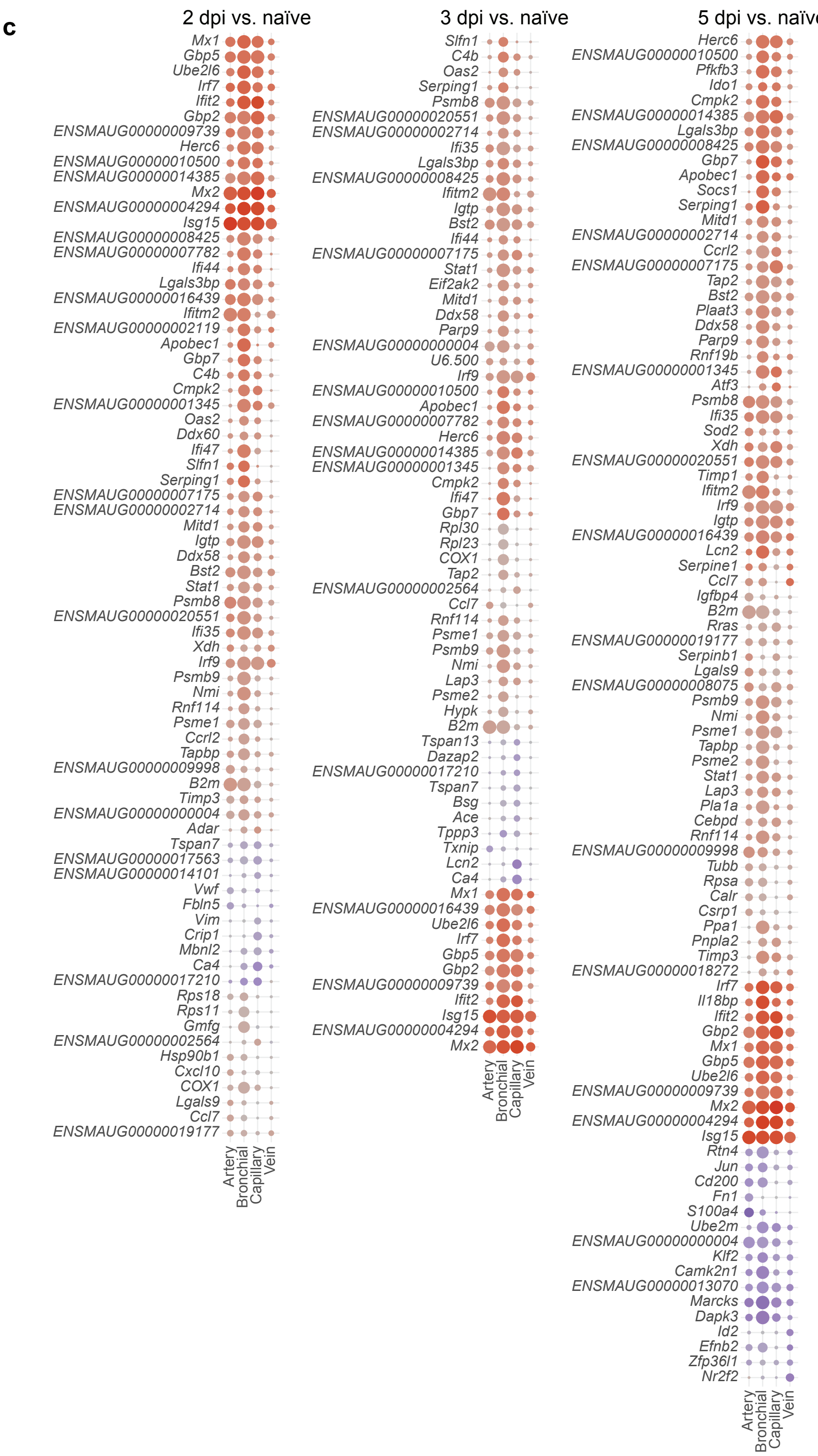

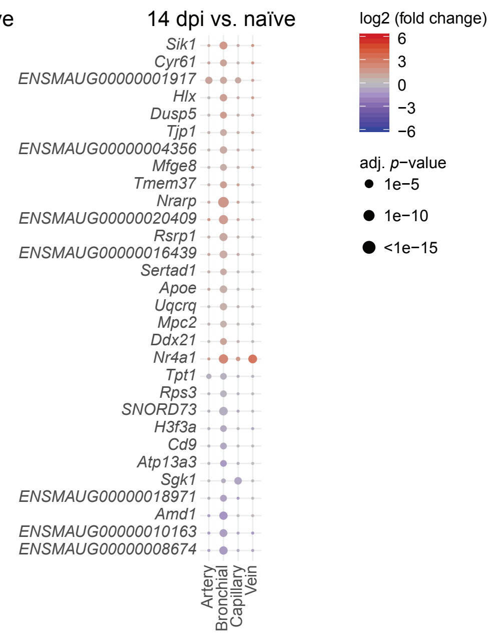

Supplementary Fig. 8: Transcriptional response of endothelial subtypes to SARS-CoV-2 infection. (a), Heatmap of cell type-marker gene expression in identified clusters for endothelial cell (EC) subtypes, based on classical cell type-markers. Normalized average gene expression levels for cells in a cluster are indicated by coloration. (b), Percentage of lung endothelial cell subpopulations for each time point (2, 3, 5 and 14 days post infection (dpi)) and control groups (naïve). Data display means and individual values ± SD, n = 3 per time point. Bar plots are plotted per cell type in the order: naïve, 2 dpi, 3 dpi, 5, dpi and 14 dpi. (colors fade from dark to light). (c) Dotplot of differentially expressed genes in endothelial cell subtype. Shown are genes that are in at least one cell type among the top 50 most changing genes as ranked by adjusted *p*-value (only events with adjusted *p*-value > 0.01). Coloration and point size indicate log2-transformed fold changes and *p*-values, respectively, of genes at 2 dpi time point relative to control groups (naïve). Adjusted (adj.) *p*-values were calculated by DEseq2 using Benjamini-Hochberg corrections of Wald test *p*-values.

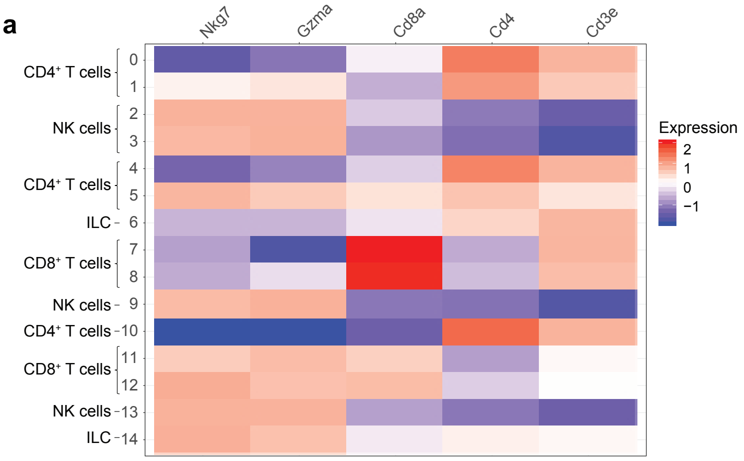

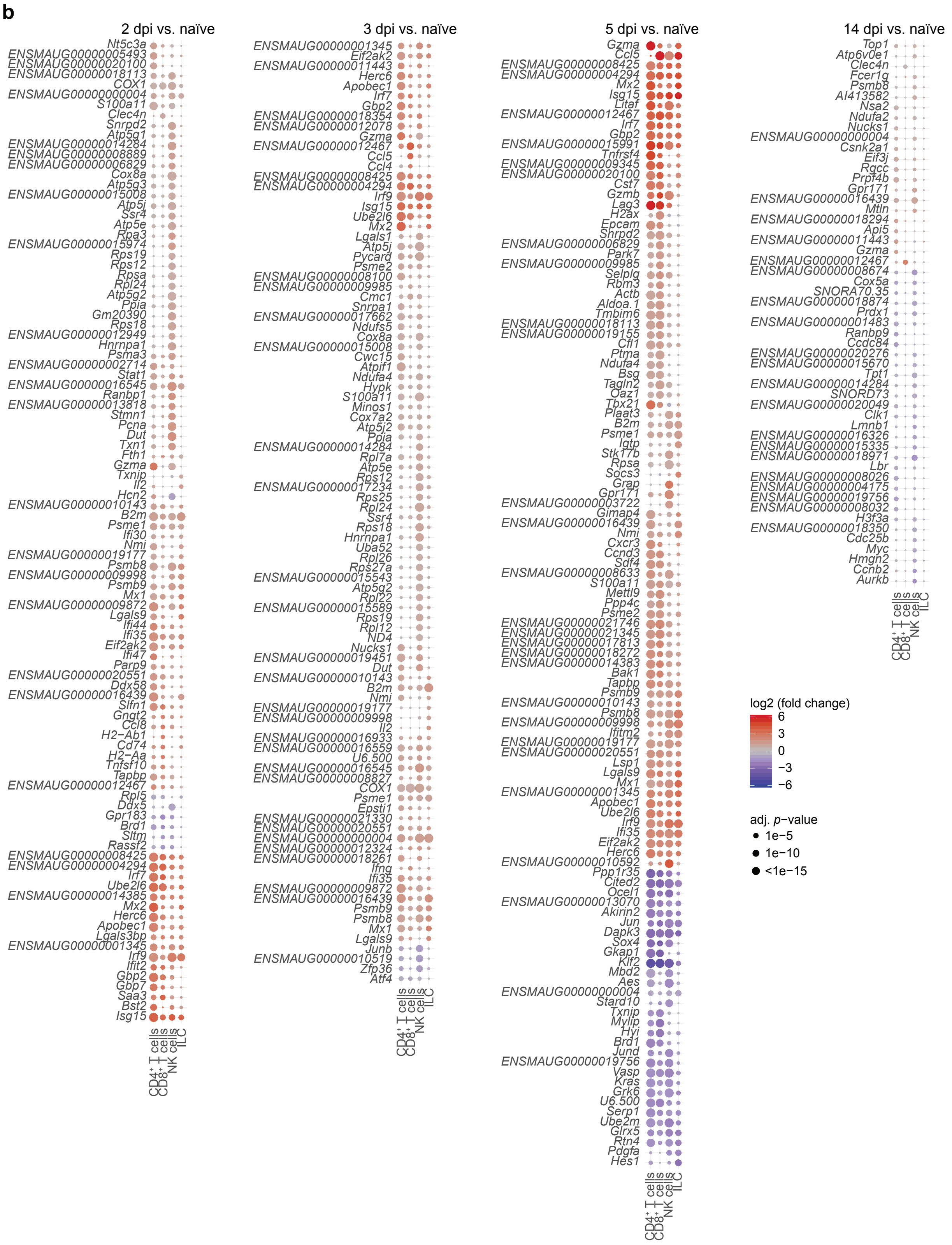

Supplementary Fig. 9: Transcriptional response of T and NK cells to SARS-CoV-2 infection. (a), Heatmap of cell type-marker gene expression in identified clusters for T and NK cell subtypes, based on classical cell type-markers. Normalized average gene expression levels for cells in a cluster are indicated by coloration. (b) Dotplot of differentially expressed genes in T and NK cell subtype. Shown are genes that are in at least one cell type among the top 50 most changing genes as ranked by adjusted (adj.) *p*-value (only events with adjusted *p*-value larger 0.01). Coloration and point size indicate log2-transformed fold changes and *p*-values, respectively. Adjusted *p*-values were calculated by DEseq2 using Benjamini-Hochberg corrections of Wald test *p*-values. NK: natural killer cells, ILCs: Innate lymphoid cells.

Supplementary Table 1: Oligonucleotides used in this study.

**Primer/probe**	**Sequence 5'–3'**
SARS-CoV-2 forward	ACAGGTACGTTAATAGTTAATAGCGT
SARS-CoV-2 reverse	ATATTGCAGCAGTACGCACACA
SARS-CoV-2 probe	FAM-ACACTAGCCATCCTTACTGCGCTTCG-BHQ
RPL-18 forward	GTTTATGAGTCGCACTAACCG
RPL-18 reverse	TGTTCTCTCGGCCAGGAA
RPL-18 probe	FAM-TCTGTCCCTGTCCCGGATGATC-BHQ

Supplementary Table 2. Identified proteins in respective proteome analysis

**Differentially regulated in lung and reported to be regulated in human BAL fluid (22 proteins)**		**Showing the same direction of change (10 proteins)**	**Differentially regulated proteins in lung that have been reported to be regulated in human plasma (13 proteins)**
Sept11	Ctsz	Sept11	C4b
Sod2	Grb2	Sod2	Ace
Hspa5	Eif6	Hspa5	S100a8
Tppp3	Gprc5a	Tppp3	Serpind1
Galm	Rps19	Galm	Ighm
Csrp1	Lap3	Csrp1	Hp
Dpysl2	Cd9	Dpysl2	B2m
Vat1	Samhd1	Vat1	Hpx
Igkv7-33	Aqp5	Igkv7-33	Itih3
Lgals3bp		Lgals3bp	Serping1
Cotl1			Igkv7-33
Pls3			Cfi
Psme1			Lgals3bp

**Showing the same trend (9 proteins)**	**Differentially expressed in serum (37 proteins)**		**Proteins reported in human COVID19 studies (20 proteins)**	
S100a8	Apoc1	Cfd	Apoc1	Actg1
Ighm	Cetp	Aldh1a1	C4b	
Hp	C4b	Pon1	Gsn	
B2m	Smco3		C6	
Itih3	Gsn	Apoc3	Apoa1	
Serping1	C6	Mbl1	Clu	
Igkv7-33	Apoa1	Clec3b	Serping1	
Cfi	Hbae5	Hpx	Hp	
Lgals3bp	Igkv7-33		Hpx	
	Clu	Hbb-y	Lbp	
	Serping1	Agt	Itih3	
	Hp	Calb1	Rbp4	
	Adipoq	Actg1	Ttr	
	Dusp2	Amy1	Cfd	
	Pltp	Aldob	Pon1	
	Cndp1	ENSMAUG00000021094	Apoc3	
	Lbp	ENSMAUG00000016284	Clec3b	
	Itih3	ENSMAUG00000001023	Igkv7-33	
	Rbp4	ENSMAUG00000004376	Agt	
	Ttr			

Supplementary Table 3. Frequencies (%) of *Ace2*, Tmprss2, *Furin*, *Bsg*, *Nrp1, Ext1* mRNA–positive and SARS-CoV-2 positive cells amongst indicated cell populations.

Gene	Day (d)	Alveolar epithelial cells type 1	Alveolar epithelial cells type 2	Ciliated epithelial cells	Endothelial cells	Fibroblasts	Alveolar macrophages	Monocytic macrophages	Interstitial macrophages
Ace2	0 dpi (naïve)	0.43±0.74	3.70±1.20	21.82±13.73	0.06±0.11	0.00	1.10±0.36	0.28±0.34	1.38±1.59
	2 dpi	0.98±1.70	3.79±1.00	4.44±7.70	0.00	0.00	1.09±0.58	0.30±0.19	0.50±0.86
	3 dpi	0.67±1.15	3.53±1.24	10.13±8.77	0.00	0.00	0.90±0.23	0.23±0.21	0.46±0.80
	5 dpi	0.00	4.83±1.52	0.00	0.00	0.00	0.14±0.25	0.25±0.16	1.10±1.28
	14 dpi	1.85±1.89	4.60±0.75	7.25±2.38	0.00	0.00	1.15±0.61	0.09±0.15	0.69±1.20
Tmprss2	0 dpi (naïve)	73.68±5.59	12.00±1.86	34.55±14.43	0.13±0.11	0.00	0.00	0.06±0.11	0.00
	2 dpi	44.91±8.72	5.76±0.66	27.29±14.75	0.11±0.19	0.00	0.07±0.12	0.24±0.26	0.00
	3 dpi	54.25±5.04	9.30±1.66	27.14±5.45	0.00	0.50±0.86	0.13±0.11	0.09±0.08	0.00
	5 dpi	56.19±18.23	11.98±2.16	6.58±7.93	0.00	0.00	0.14±0.25	0.08±0.07	0.00
	14 dpi	65.57±6.49	15.43±0.53	30.98±11.78	0.36±0.23	0.00	0.10±0.18	0.00	0.00
Furin	0 dpi (naïve)	30.63±1.02	16.55±3.07	29.29±9.74	18.95±1.20	10.50±5.86	10.21±2.47	25.95±1.46	18.75±6.56
	2 dpi	28.69±6.20	16.74±3.98	31.00±8.89	17.28±0.91	18.77±2.99	13.57±2.99	23.90±6.26	16.45±6.63
	3 dpi	34.97±4.62	22.97±2.42	26.24±9.55	19.09±0.99	16.61±8.21	12.58±1.10	28.69±0.55	19.72±5.95
	5 dpi	24.11±12.96	22.74±5.35	6.58±7.93	22.19±0.79	17.62±2.39	40.03±9.12	52.76±4.78	51.63±1.61
	14 dpi	30.71±5.03	20.67±1.69	42.16±11.14	19.31±0.91	14.13±5.82	12.32±4.06	26.70±5.07	25.16±8.76
Bsg	0 dpi (naïve)	100.0±0.00	93.93±1.35	96.97±5.25	94.36±3.50	96.07±2.40	95.10±1.37	76.55±7.16	83.81±3.96
	2 dpi	99.49±0.89	90.65±4.77	95.21±4.18	92.69±5.16	96.92±2.07	92.28±4.19	77.19±7.13	84.03±9.83
	3 dpi	98.73±1.11	92.80±0.95	96.30±6.42	95.12±2.42	98.71±1.20	93.39±1.62	80.17±1.03	85.90±2.87
	5 dpi	100.00±0.00	99.46±0.93	92.34±7.20	99.24±1.10	100.00±0.00	91.50±2.72	92.23±1.11	97.43±1.53
	14 dpi	99.70±0.52	97.71±0.62	98.04±3.40	98.11±1.55	100.00±0.00	97.28±0.82	87.00±2.76	95.36±2.10
Nrp1	0 dpi (naïve)	0.44±0.76	32.06±6.04	4.14±4.60	54.92±10.61	12.65±3.25	0.25±0.44	1.40±0.65	7.81±7.34
	2 dpi	0.90±0.80	32.99±5.15	1.28±2.22	45.48±9.69	10.75±5.14	0.22±0.37	2.18±0.98	6.43±4.89
	3 dpi	0.95±1.65	41.46±7.27	1.67±2.89	57.70±5.56	19.76±12.25	0.32±0.56	1.97±0.68	6.98±2.44
	5 dpi	0.72±1.26	35.24±4.96	8.96±5.74	55.25±8.27	10.16±5.77	12.33±6.84	16.66±2.06	34.58±15.48
	14 dpi	1.74±0.78	35.94±2.55	0.00	68.53±2.41	25.47±8.05	0.30±0.05	1.86±0.29	9.47±2.81
Ext1	0 dpi (naïve)	22.43±3.37	12.37±1.46	5.25±4.71	8.14±1.62	20.62±6.23	17.74±2.64	6.81±1.25	8.14±1.20
	2 dpi	12.66±5.06	9.73±3.97	9.97±11.29	7.38±3.38	17.89±4.81	11.46±2.73	7.48±1.39	8.75±4.24
	3 dpi	17.70±7.72	12.37±1.85	33.65±20.33	11.76±0.53	35.84±12.53	16.41±2.84	12.86±1.65	10.97±2.54
	5 dpi	19.89±7.84	15.24±5.47	8.36±8.71	9.06±1.21	25.12±0.59	22.93±5.25	27.84±1.82	27.74±4.85
	14 dpi	20.68±3.15	10.34±2.58	7.25±6.34	10.73±2.82	37.87±7.61	10.20±3.68	5.73±1.72	7.75±0.82
SARS-Cov-2	2 dpi	6.01±7.89	6.41±6.55	6.07±5.79	4.95±5.91	4.46±6.70	7.85±6.82	10.24±6.67	9.39±7.33
	3 dpi	10.48±13.31	12.05±6.41	15.50±4.45	9.15±7.70	10.05±6.01	12.20±8.66	11.47±7.40	15.76±11.03
	5 dpi	4.17±7.22	2.76±3.91	7.84±0.79	1.88±1.63	2.14±2.52	24.77±0.31	9.65±2.32	15.74±5.02
	14 dpi	0.00	0.00	0.00	0.00	0.00	0.00	0.00	0.00

[1] Data are represented as mean ± standard deviation. *Ace2*, Angiotensin-converting enzyme 2, *Tmprss2,* transmembrane serine protease 2, *Bsg*, Basigin, *Nrp1*, neuropilin, *Ext1* exostosin glycosyltransferase 1, SARS-CoV-2, severe acute respiratory syndrome coronavirus 2, dpi, days post infection

Supplementary Notes

PCA on bulk transcriptomic data.

A principal component analysis (PCA) of the bulk RNA-sequencing data from lung and blood is shown in Supplementary Note Fig. 1. A key feature of both tissue types is the separation of the 5 days post infection (dpi) samples, which is reflected by strong alterations in gene expression values seen in Supplementary Fig. 4 and 5. As expected, lung samples from naïve and 14 dpi animals on one hand cluster together, and from 2 dpi and 3 dpi on the other hand (Supplementary Note Fig. 1, left). Interestingly however, this was not the case for blood samples (Supplementary Note Fig. 1, right), where, apart from day 5, samples did not cluster per time point. As detailed below, we took sex into account as a covariate in the proteomics PCA, which was however not possible for transcriptomics due to the limited number of samples.

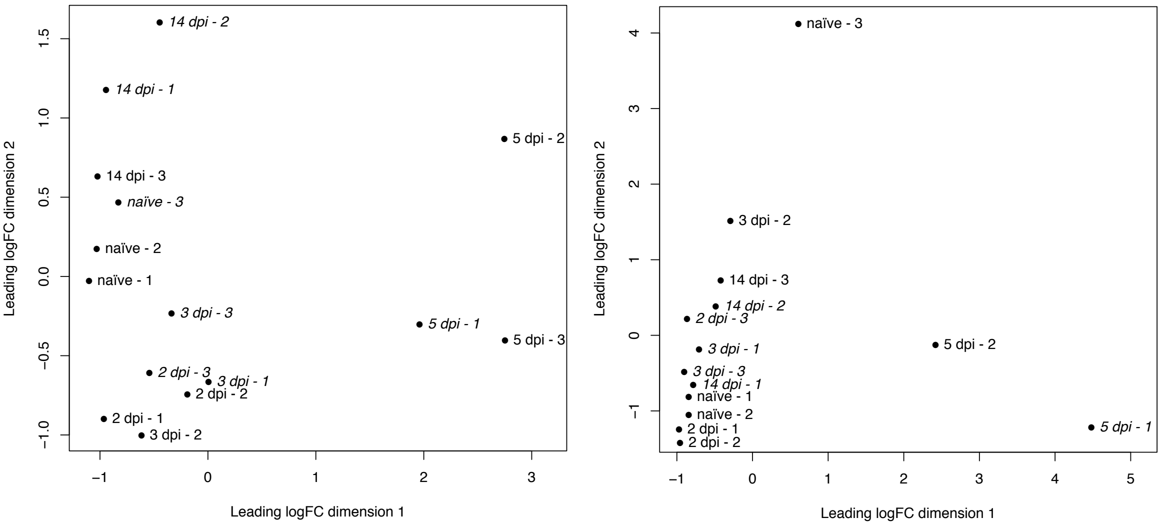

Supplementary Note Fig. 1: Principal component analysis (PCA) of the bulk RNA-sequencing data of lung (left) and blood (right) data using the multi-dimensional scaling function from the edgeR package [6]. Female animals are printed in italics. Sample 5 days post infection (dpi) – n°3 from blood could not be sequenced due to RNA degradation. LogFC: log fold change.

PCA on bulk proteomics data.

Similarly, on the proteome level, the disease effect drives the variance in the dataset. Serum samples from infected animals (2, 3, and 5 dpi) cluster together and are separate from control samples immediately at 2 dpi. In contrast, the response in lung is delayed, peaks at 5 dpi and largely resolves until 14 dpi (see Supplementary Note Fig. 2). The exploratory analysis is in strong agreement with the trends observed for

differentially regulated proteins (Figures 2b, 2c and Supplementary Fig. 4h). Based on the PCA results, gender was included as an additional factor in linear modelling. Post-hoc analysis however revealed only minor influence of gender on disease-regulated protein expression and showed that there is no interaction with disease (see Supplementary Note Fig. 3).

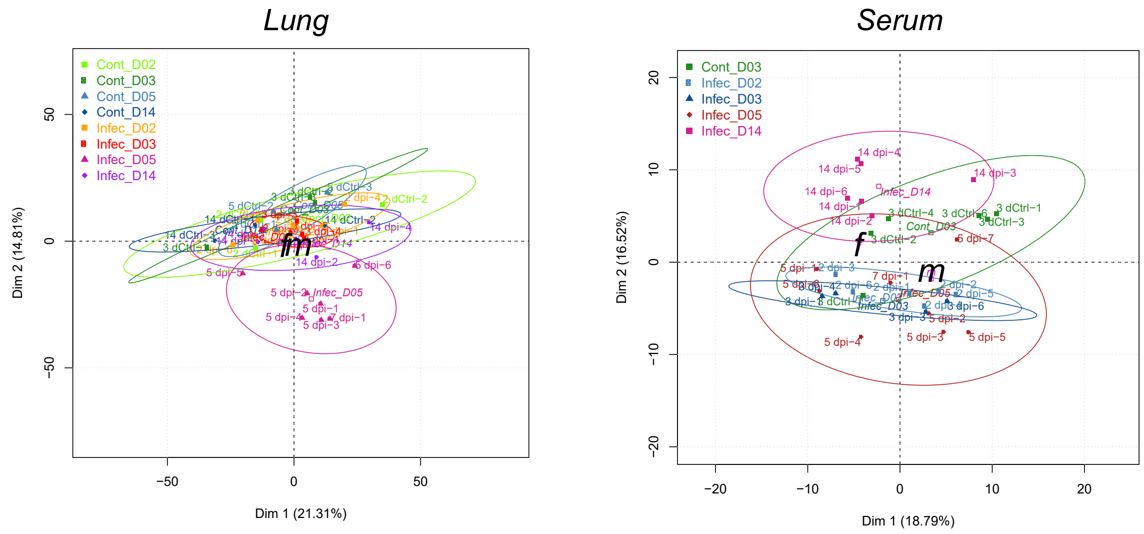

Supplementary Note Fig. 2: Principal component analysis (PCA) scores plot for lung (left) and serum (right) samples. The centers of gravity of females and males (identified by black letters f and m) are close to the coordinates origin and can hardly be distinguished in the lung plot (left), whereas they are clearly separated in the serum plot (right). One can also see that the infected 7 dpi-1 sample (the only one that we had) clusters together with infected samples at 5 dpi. Therefore, it was included in the group of infected 5 dpi samples for linear modeling. Dpi: days post infection.

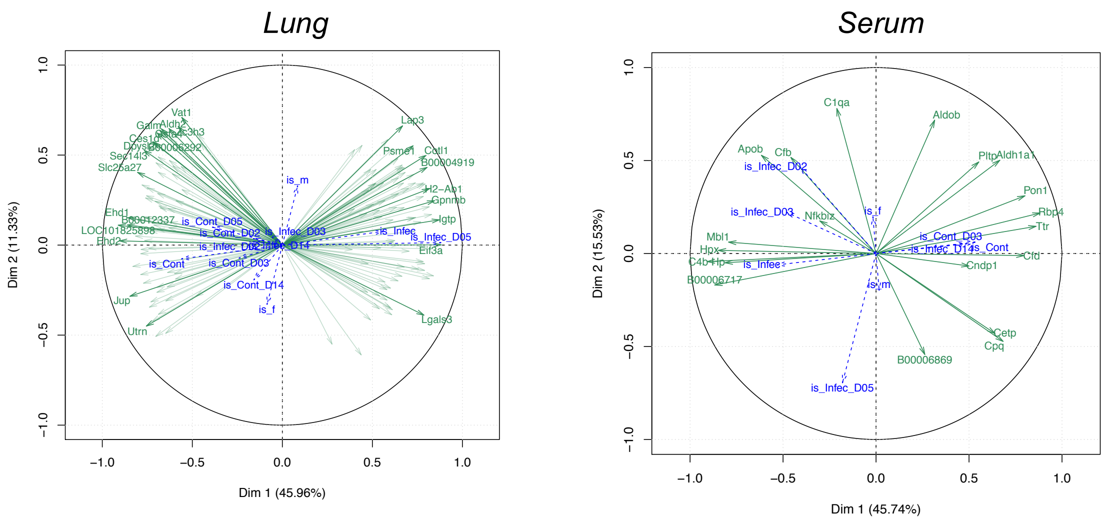

Supplementary Note Fig. 3: Results of post-hoc principal component analysis (PCA) using regulated proteins for contrast infected vs. controls at 5 dpi for lung samples (left panels) and using regulated proteins for contrast infected vs. control at 3 dpi for serum samples (right

panel). PCA loading plots are presented. The gender effect (arrows is_f, is_m) is small and independent from disease effect (arrows is_Infec, is_Cont). Dim: dimensional reduction plot.

Assessing viral replication from sequencing data.

In order to investigate, whether the single-cell sequencing data could provide evidence for viral replication in specific cell types, we applied two previously published approaches [7, 8]. The first one relies on the fact that large numbers of subgenomic viral RNAs are generated in productive replication, and their frequency patterns can therefore be used to distinguish productive from unproductive infection. Although single-cell sequencing captures polyA tails, and thus the sequencing reads are mostly aligning to the 3'-end of the viral RNA, mispriming can happen throughout the viral RNA, and particularly near poly-A stretches. Differences in the read distribution pattern can then be used to provide evidence whether there is viral replication in some cell types[8].

To assess relative expression of viral transcripts, we extracted UMI counts for all viral genes (including the 3'UTR) and assessed the number of cells positive for a specific viral gene per (coarse) cell type and sample. We then plotted the number of cells positive for viral genes relative to the number of cells positive for the 3'UTR (Supplementary Note Fig. 4).

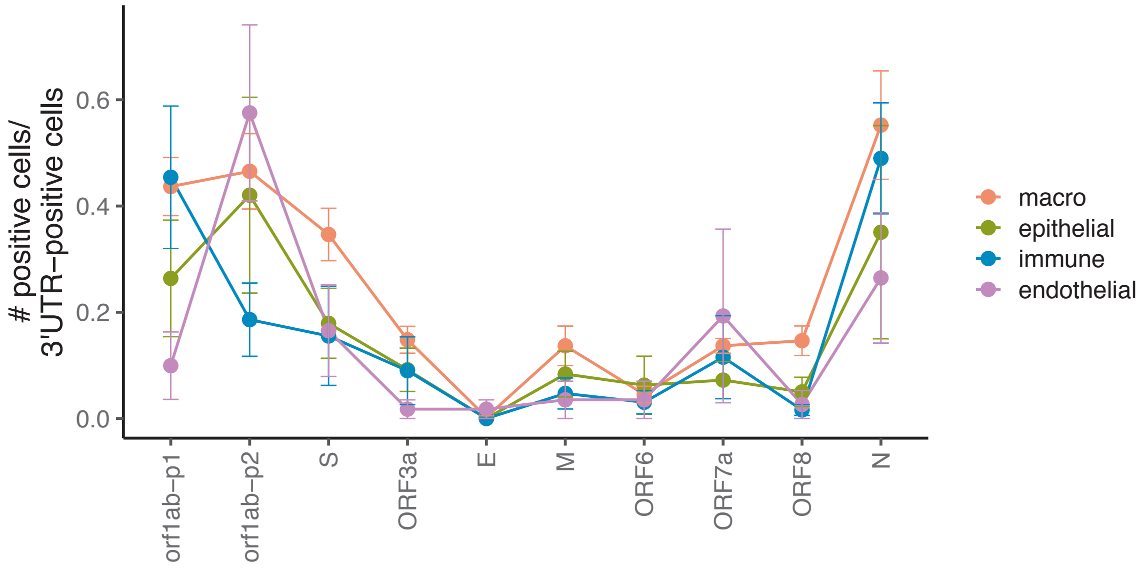

Supplementary Note Fig. 4: Fraction of cells containing reads from various regions of the viral genome in four different cell type classes, normalized by cells positive for the 3' end. Error bars denote mean +/- s.e.m. Since the beginning of Orf1ab contains extended 5' leaders, we divided this region in two halves (p1 and p2). To reduce noise, cells were grouped as following: macro, alveolar/interstitial/monocytic macrophages and *Treml4*+ monocytes; epithelial,

alveolar epithelial cells type 1 and 2 and ciliated cells; immune, B cells and T/NK cells; endothelial, all endothelial cells. Data from n = 3 animals per time point.

Overall, we did not find significant differences between the cell types, indicating that this method is not sensitive enough to distinguish the *bona fide* replication in epithelial cells from other cells, where replication is unlikely, such as B and T cells ("immune" in the figures above).

A second method relies on the presence of minus strand viral RNA, which is also a hallmark of viral replication [7]. To measure the level of antisense reads, we counted the number of reads in sense and antisense direction to a specific gene locus associated with each cell barcode. We then aggregated counts from cells of a certain (coarse) cell type and a specific sample and computed the percentage of antisense reads relative to the combined number of sense and antisense reads. Reads from the viral genome are then compared to ubiquitously and highly expressed genes (*Eef1a1, Eef2, Rps3*) with no known antisense transcripts as controls. The percentage of antisense RNA (or minus-strand RNA for the virus) is plotted for the different cell types and infection days in Supplementary Note Fig. 5.

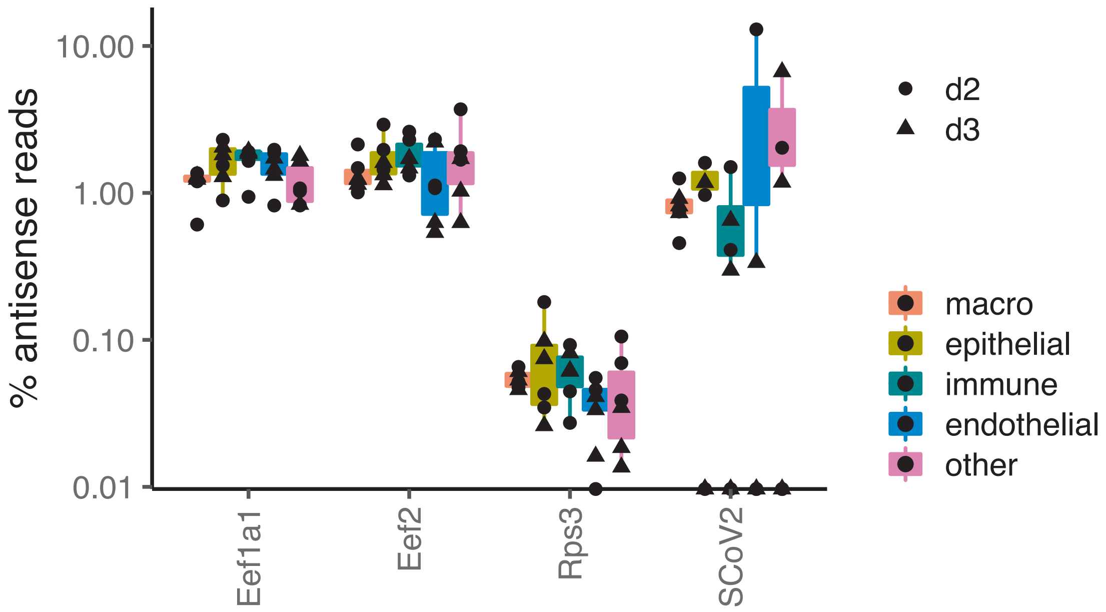

Supplementary Note Fig. 5: Percentage of antisense/minus strand RNA for three host genes and viral RNA. To reduce noise, cells were grouped as following: macro, alveolar/interstitial/monocytic macrophages and *Treml4*$^{+}$ monocytes; epithelial, alveolar epithelial cells type 1 and 2 and ciliated cells; immune, B cells and T/NK cells; endothelial, all endothelial cells. Data from n = 3 animals per time point. For all boxplots, lower and upper hinges correspond to first and third quartiles and whiskers extend to a maximum of 1.5 times

the distance between first and third quartile. Individual data points are shown by circles (2 dpi) and triangles (3 dpi).

In this data, no significant difference was seen between cell types for viral RNA, but also not between viral RNA and *Eef1a1* and *Eef2*. The presence of negative-strand RNA could therefore also be due to a limited specificity of strandedness due to technical limitations of library preparation and sequencing.

In summary, we conclude that determining productive replication based on single-cell RNA-sequencing data from complex samples is not achievable with confidence in our data. However, accumulating literature indicates that replication happens primarily in epithelial cells, and to a much smaller degree in e.g. macrophages (see also [1] for RNA scope analysis in macrophages). All code is available at the dedicated github page (https://github.com/Berlin-Hamster-Single-Cell-Consortium/Single-cell-sequencing-of-COVID-19-pathogenesis-in-golden-Hamsters-).

Elongation of 3'-UTRs in the Ensembl 99 MesAur 1.0 annotation

Upon inspection of the bulk RNA-sequencing alignments, we observed that in the Ensembl annotation for the MesAur 1.0 version of the *M. auratus* genome, the alignments frequently extended the existing 3'-UTR. An example is the coverage of the *Cxcl10* gene in the bulk and single-cell RNA-sequencing (sample: 2 dpi, animal 2) in Supplementary Note Fig. 6.

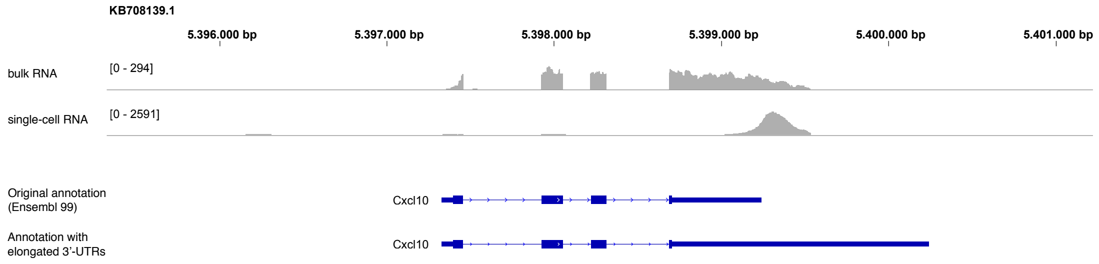

Supplementary Note Fig. 6: Coverage profiles of bulk RNA-sequencing (top) and single-cell RNA-sequencing over the *Cxcl10* gene. Underneath is the annotation shown for the original annotation and the 3'-UTR elongated by 1000 base pairs (bp).

We therefore decided to globally extend all 3'-UTRs by 1000 bp, using the code described in the NCBI GEO entry (https://www.ncbi.nlm.nih.gov/geo/query/acc.cgi?acc=GSM4946639), where the modified annotation file (gtf format) is available.

In order to assess the gain of sensitivity by this extension, we first counted a bulk RNA-seq sample (day 2, animal 2) using FeatureCount [9]:

Original Annotation:

Assigned	5,856,729
Unassigned_MultiMapping	639,605
Unassigned_NoFeatures	3,874,788
Unassigned_Ambiguity	27,205

Annotation with longer 3'-UTRs:

Assigned	6,065,729
Unassigned_MultiMapping	639,605
Unassigned_NoFeatures	3,448,334
Unassigned_Ambiguity	244,659

The elongated 3'-UTRs therefore allowed the assignment of about 210,000 more reads (3.6%) in the bulk RNA-sequencing.

Since, as visible above, the scRNA-seq sequencing reads are particularly prone to not be counted if the 3'-UTR were too short, we plotted, again for 2 dpi, animal 2, the single-cell RNA-seq gene count over all cells for the original annotation and the one with the elongated 3'-UTRs (Supplementary Note Fig. 7). We found about 5,000 genes to have an increase of 10 % or more counts using the modified annotation, with only a few having less counts.

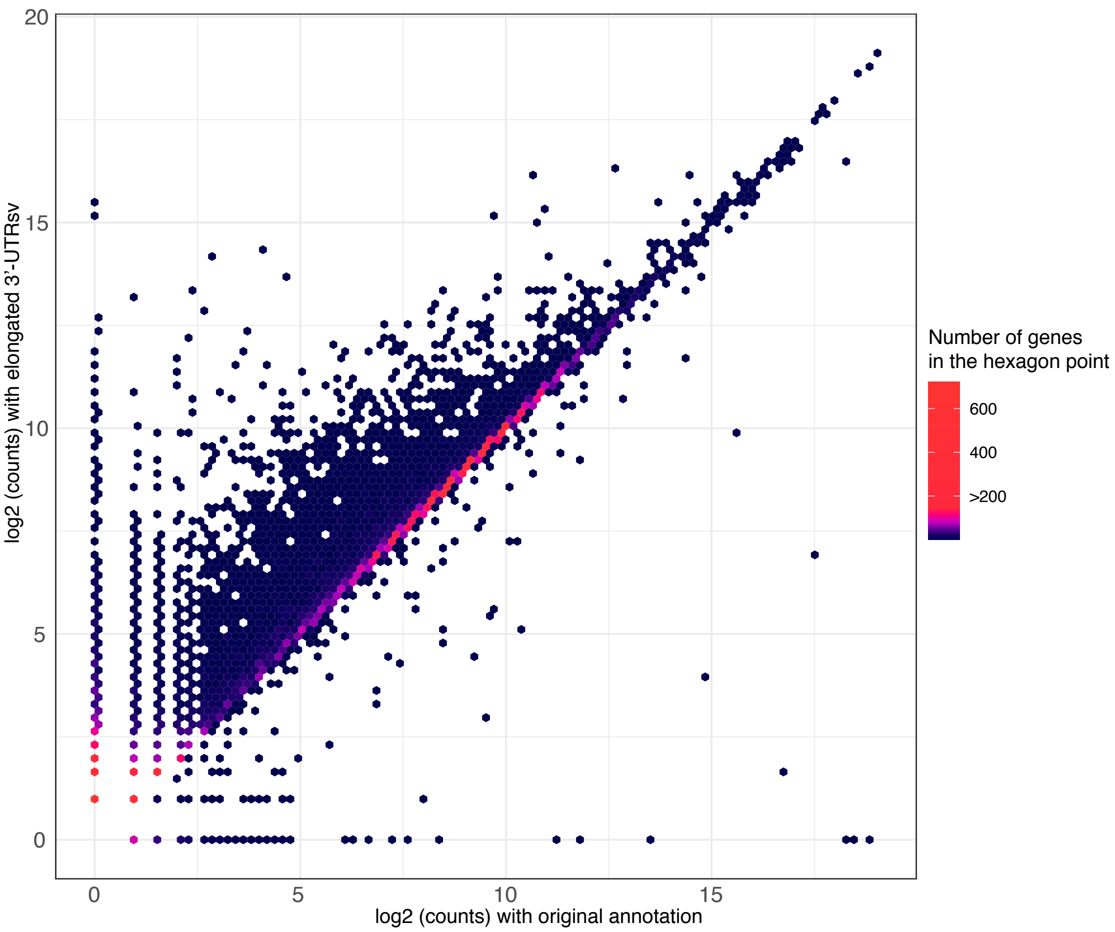

Supplementary Note Fig. 7: Density plot of log(2) transformed gene counts in the scRNA-seq data from day 2, animal 2, using the original annotation (horizontal axis) and the annotation with elongated 3'-UTRs (vertical axis).

Overall, this data indicates that until a more precise annotation is available, the global elongation of all 3'-UTRs by 1,000 bp provides a reasonable proxy for a better gene expression profiling.

Supplementary References

1. Osterrieder N, et al. Age-Dependent Progression of SARS-CoV-2 Infection in Syrian Hamsters. Viruses 12, (2020).
2. Schauer AE, et al. IL-37 Causes Excessive Inflammation and Tissue Damage in Murine Pneumococcal Pneumonia. J Innate Immun 9, 403-418 (2017).

3. Winkler ES, et al. SARS-CoV-2 infection of human ACE2-transgenic mice causes severe lung inflammation and impaired function. Nat Immunol 21, 1327-1335 (2020).
4. Liao M, et al. Single-cell landscape of bronchoalveolar immune cells in patients with COVID-19. Nat Med 26, 842-844 (2020).
5. Chua RL, et al. COVID-19 severity correlates with airway epithelium-immune cell interactions identified by single-cell analysis. Nat Biotechnol 38, 970-979 (2020).
6. Robinson MD, McCarthy DJ, Smyth GK. edgeR: a Bioconductor package for differential expression analysis of digital gene expression data. Bioinformatics 26, 139-140 (2010).
7. Grant RA, et al. Circuits between infected macrophages and T cells in SARS-CoV-2 pneumonia. Nature 590, 635-641 (2021).
8. Speranza E, et al. Single-cell RNA sequencing reveals SARS-CoV-2 infection dynamics in lungs of African green monkeys. Sci Transl Med 13, (2021).
9. Liao Y, Smyth GK, Shi W. featureCounts: an efficient general purpose program for assigning sequence reads to genomic features. Bioinformatics 30, 923-930 (2014).
