# Supplementary material for: Temporal omics analysis in Syrian hamsters unravel cellular effector responses to moderate COVID-19
Source: Nat Commun. 2021 Aug 11;12:4869. doi: 10.1038/s41467-021-25030-7 (PMC8357947; doi:10.1038/s41467-021-25030-7)
Supplement: Supplementary file 2 — Reporting summary [file 41467_2021_25030_MOESM2_ESM.pdf]

nature research

Corresponding author(s): Geraldine Nouailles, Emanuel Wyler, Jakob Trimpert, Martin Witzenrath

Last updated by author(s): Jun 23, 2021

Reporting Summary

Nature Research wishes to improve the reproducibility of the work that we publish. This form provides structure for consistency and transparency in reporting. For further information on Nature Research policies, see our Editorial Policies and the Editorial Policy Checklist.

Statistics

For all statistical analyses, confirm that the following items are present in the figure legend, table legend, main text, or Methods section.

n/a	Confirmed	
☐	☒	The exact sample size (*n*) for each experimental group/condition, given as a discrete number and unit of measurement
☐	☒	A statement on whether measurements were taken from distinct samples or whether the same sample was measured repeatedly
☐	☒	The statistical test(s) used AND whether they are one- or two-sided<br>*Only common tests should be described solely by name; describe more complex techniques in the Methods section.*
☐	☒	A description of all covariates tested
☐	☒	A description of any assumptions or corrections, such as tests of normality and adjustment for multiple comparisons
☐	☒	A full description of the statistical parameters including central tendency (e.g. means) or other basic estimates (e.g. regression coefficient) AND variation (e.g. standard deviation) or associated estimates of uncertainty (e.g. confidence intervals)
☐	☒	For null hypothesis testing, the test statistic (e.g. *F*, *t*, *r*) with confidence intervals, effect sizes, degrees of freedom and *P* value noted<br>*Give P values as exact values whenever suitable.*
☒	☐	For Bayesian analysis, information on the choice of priors and Markov chain Monte Carlo settings
☒	☐	For hierarchical and complex designs, identification of the appropriate level for tests and full reporting of outcomes
☒	☐	Estimates of effect sizes (e.g. Cohen's *d*, Pearson's *r*), indicating how they were calculated

Our web collection on statistics for biologists contains articles on many of the points above.

Software and code

Policy information about availability of computer code

Data collection	bcl2fastq 2.20.0, cellranger 3.1.0
Data analysis	GraphPad Prism 9.1.2, R version 3.6.3, SCIEX Analyst 1.8.1<br>R packages used: ggplot2 3.3.2, gprofiler 0.2.0, Seurat 3, dplyr 1.0.2, quasR 1.18, DESeq2 1.18.1, lme4 1.1-27, LIMMA 3.40.6, and dependencies<br>Code is available at https://github.com/Berlin-Hamster-Single-Cell-Consortium/Single-cell-sequencing-of-COVID-19-pathogenesis-in-golden-Hamsters- or via Zenodo, doi: 10.5281/zenodo.4983546

For manuscripts utilizing custom algorithms or software that are central to the research but not yet described in published literature, software must be made available to editors and reviewers. We strongly encourage code deposition in a community repository (e.g. GitHub). See the Nature Research guidelines for submitting code & software for further information.

Data

Policy information about availability of data

All manuscripts must include a data availability statement. This statement should provide the following information, where applicable:
- Accession codes, unique identifiers, or web links for publicly available datasets
- A list of figures that have associated raw data
- A description of any restrictions on data availability

Raw and processed data is available at the NCBI gene expression omnibus, entry GSE162208. Further supplementary data is available at http://www.mdc-berlin.de/singlecell-SARSCoV2. The mass spectrometry proteomics data have been deposited to the ProteomeXchange Consortium via the PRIDE partner repository 67 with the dataset identifier PXD025164.
DIA-NN 1.7.12 and REVIGO were accessed in April 2021. These public data sets were used: from gene expression omnibus GSE145926, GSM3660650, and doi:

10.6084/m9.figshare.12436517
The Tabula Muris data has been downloaded from https://figshare.com/articles/dataset/Robject_files_for_tissues_processed_by_Seurat/5821263, the human lung cell atlas data from https://www.synapse.org/#!Synapse:syn21041850/wiki/600865

Field-specific reporting

Please select the one below that is the best fit for your research. If you are not sure, read the appropriate sections before making your selection.

☒ Life sciences ☐ Behavioural & social sciences ☐ Ecological, evolutionary & environmental sciences

For a reference copy of the document with all sections, see nature.com/documents/nr-reporting-summary-flat.pdf

Life sciences study design

All studies must disclose on these points even when the disclosure is negative.

Sample size

No sample size calculation was performed. N were chosen based on experience and sample availability. The core data in this manuscript was generated by single-cell-sequencing analysis. For every time point, three different animals were used. A sample size of 3 animals per time-point was chosen as the necessary minimum to perform statistical analysis on results obtained in this study. For analysis of the clinical course and histopathology of SARS-CoV-2 infection in Syrian hamsters, the entire available cohort of N = 6 hamsters per time point was used. N = 6 was deemed appropriate to evaluate the course of infection in an already well described model. Proteome analysis was performed on samples used to analyze single-cell transcriptomes and available samples from the hamster cohort. The analyzed sample size was sufficient for descriptive analysis, following the 3R principle, no further animals were used.

Data exclusions

No data was excluded in the analysis of transcriptomes, histopathology, virology, and clinical parameters. For proteomics analysis, we excluded 4 samples of low technical quality after outlier analysis.

Replication

Variability between replicates (i.e. animals) is displayed in the figures by showing standard deviations.

Randomization

Animals were assigned randomly to experimental groups. No experiments other than those on animals were conducted.

Blinding

All personnel involved in animal experiments and histological analysis of samples was blinded for the duration of the experiment. Single-cell experiments were conducted unblinded, since blinding is not relevant

Reporting for specific materials, systems and methods

We require information from authors about some types of materials, experimental systems and methods used in many studies. Here, indicate whether each material, system or method listed is relevant to your study. If you are not sure if a list item applies to your research, read the appropriate section before selecting a response.

Materials & experimental systems

n/a	Involved in the study	
☒	☐	Antibodies
☐	☒	Eukaryotic cell lines
☒	☐	Palaeontology and archaeology
☐	☒	Animals and other organisms
☒	☐	Human research participants
☒	☐	Clinical data
☒	☐	Dual use research of concern

Methods

n/a	Involved in the study	
☒	☐	ChIP-seq
☒	☐	Flow cytometry
☒	☐	MRI-based neuroimaging

Eukaryotic cell lines

Policy information about cell lines

Cell line source(s)

Vero E6 cells were obtained from ATCC (ATCC CRL-1586).

Authentication

Vero E6 cells were confirmed to support SARS-CoV-2 growth by visual confirmation of cytopathic effects, by immunofluorescent staining against SARS-CoV-2 spike using infected cells and by qPCR analysis of virus gRNA. No other authentification was performed.

Mycoplasma contamination

Vero E6 cells tested negative for mycoplasma DNA by PCR, tests were performed by an independent diagnostic lab (Institut für Mikrobiologie und Tierseuchen, Freie Universität Berlin, diagnostic numbers 2079 and 2080).

Commonly misidentified lines
(See ICLAC register)

Name any commonly misidentified cell lines used in the study and provide a rationale for their use.

Animals and other organisms

Policy information about studies involving animals; ARRIVE guidelines recommended for reporting animal research

Laboratory animals	Mesocricetus auratus, RHJan:Aura (Janvier), 10-12 weeks of age at day of infection. For transcriptome studies, 5 females and 7 males were taken. For proteomics, an additional 16 females and 15 males were used.
Wild animals	The study did not involve wild animals.
Field-collected samples	The study did not contain field-collected samples.
Ethics oversight	Landesamt für Gesundheit und Soziales (LAGeSo), Berlin, Germany<br>Tierschutzbeauftragte, Freie Universität Berlin

Note that full information on the approval of the study protocol must also be provided in the manuscript.
